# The causal relationship between sarcoidosis and autoimmune diseases: a bidirectional Mendelian randomization study in FinnGen

**DOI:** 10.3389/fimmu.2024.1325127

**Published:** 2024-04-22

**Authors:** Di Sun, Ruimin Ma, Jingwei Wang, Yuanying Wang, Qiao Ye

**Affiliations:** Department of Occupational Medicine and Toxicology, Clinical Center for Interstitial Lung Diseases, Beijing Institute of Respiratory Medicine, Beijing Chao-Yang Hospital, Capital Medical University, Beijing, China

**Keywords:** sarcoidosis, autoimmune diseases, Mendelian randomization, FinnGen, causality

## Abstract

**Background:**

Sarcoidosis has been considered to be associated with many autoimmune diseases (ADs), but the cause-and-effect relationship between these two diseases has not been fully explored. Therefore, the objective of this study is to explore the possible genetic association between sarcoidosis and ADs.

**Methods:**

We conducted a bidirectional Mendelian randomization (MR) study using genetic variants associated with ADs and sarcoidosis (4,041 cases and 371,255 controls) from the FinnGen study. The ADs dataset comprised 96,150 cases and 281,127 controls, encompassing 44 distinct types of autoimmune-related diseases. Subsequently, we identified seven diseases within the ADs dataset with a case size exceeding 3,500 and performed subgroup analyses on these specific diseases.

**Results:**

The MR evidence supported the causal association of genetic predictors of ADs with an increased risk of sarcoidosis (OR = 1.79, 95% CI = 1.59 to 2.02, *P* _IVW-FE_ = 1.01 × 10^-21^), and no reverse causation (OR = 1.05, 95% CI 0.99 to 1.12, *P*_IVW-MRE_ = 9.88 × 10^-2^). Furthermore, subgroup analyses indicated that genetic predictors of type 1 diabetes mellitus (T1DM), celiac disease, and inflammatory bowel disease (IBD) were causally linked to an elevated risk of sarcoidosis (All *P* < 6.25 × 10^-3^). Conversely, genetic predictors of sarcoidosis showed causal associations with a higher risk of type 1 diabetes mellitus (*P* < 6.25 × 10^-3^).

**Conclusion:**

The present study established a positive causal relationship between genetic predictors of ADs (e.g. T1DM, celiac disease, and IBD) and the risk of sarcoidosis, with no evidence of reverse causation.

## Introduction

Sarcoidosis is a systemic granulomatous inflammatory disease characterized histologically by non-caseating granulomas in multiple organs, predominantly involving the lungs (1). The pathogenesis of sarcoidosis remains unclear and may involve genetic susceptibility, environmental factors, and immunopathogenic mechanisms (1). While the 5-year mortality rate in patients with sarcoidosis is only 7% (2), the disease is not benign for many patients due to its high burden and excess mortality (2–4). The incidence of sarcoidosis varies significantly by age, sex, and race (5), with the highest rates observed in Finland (28.2 per 100,000) (6), lower in North America and Australia (5–10) (7–9), and Asians have the lowest rates (0.5-1) (6, 10).

Autoimmune diseases (ADs) encompass a diverse range of diseases marked by the loss of self-tolerance and the production of autoantibodies. A robust association exists between these diseases and genetic susceptibility (11). Growing evidence has identified an association between sarcoidosis and ADs, particularly type 1 diabetes mellitus (T1DM), celiac disease, and inflammatory bowel disease (IBD) (12–18). However, all the associations between sarcoidosis and ADs mentioned above were derived from cross-sectional studies, leaving the causal nature of these connections elusive (19). Establishing causal relationships not only deepens the understanding of sarcoidosis and ADs pathogenesis but also has the potential to guide pathogenesis-oriented interventions against sarcoidosis and ADs in clinical settings. Therefore, there is an urgent need to elucidate the causal relationship between sarcoidosis and various types of ADs.

Mendelian randomization (MR) employs genetic markers of an exposure, specifically utilizing single nucleotide polymorphisms (SNPs) as instrumental variables (IVs), to establish causal relationships between an exposure and an outcome in the analysis (20–22). Consequently, genetic variants associated with sarcoidosis and autoimmune diseases (ADs) serve as proxies, enabling the derivation of unconfounded estimates for the associations between sarcoidosis and ADs. Numerous loci contributing to human complex traits, including sarcoidosis and ADs, have been identified through genome-wide association studies (GWAS) (23). These findings provide a significant opportunity to explore potential causal associations between them using an MR approach.

Therefore, in this study, we performed a systematic bidirectional MR analysis to investigate the causal relationship between sarcoidosis and ADs.

## Materials and methods

### Study design

**Figure 1:**
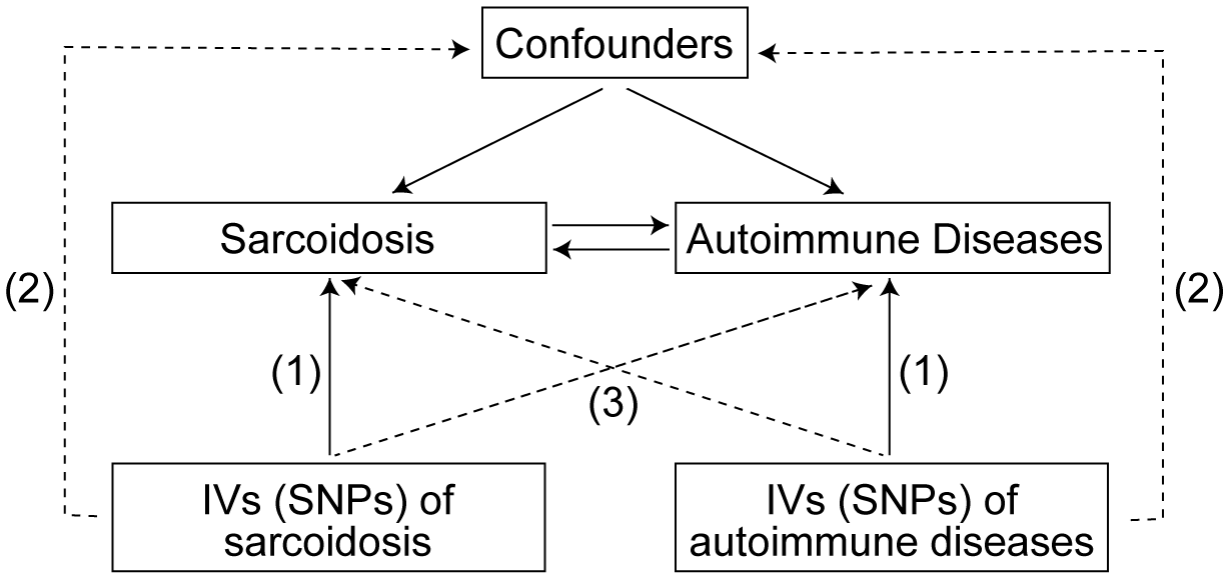
Three assumptions for IVs in MR study. IVs, Instrumental variables; SNPs, Single nucleotide polymorphisms; MR, Mendelian randomization.

The SNPs representing global human genetic variation were selected as IVs in this study. To satisfy the assumptions of MR, these IVs must satisfy three key criteria (24): (1) strong associations with the exposure of interest, (2) lack of association with confounding factors, and (3) absence of direct influence on the outcome apart from the exposure (**Figure 1**). Subsequently, a bidirectional MR analysis was conducted to evaluate the association between sarcoidosis and ADs. The study adheres to the reporting guidelines outlined in the Guidelines for Strengthening the Reporting of Mendelian Randomization Studies (STROBE-MR) checklist (25, 26).

### Data source

Currently, there is no specialized GWAS data specifically dedicated to ADs available globally. Therefore, we selected a dataset consisting of 96,150 cases and 281,127 controls to encompass a wide range of ADs, including 44 different types of autoimmune-related diseases (**Supplementary Table 1**). We identified diseases within the dataset that had a case size exceeding 3,500 cases and conducted subgroup analyses specifically for these diseases. The diseases included in the subgroup analyses, along with their respective case and control sizes, are as follows (**Supplementary Table 2**): rheumatoid arthritis (12,555 cases and 240,862 controls), autoimmune hypothyroidism (40,926 cases and 274,069 controls), T1DM (4,196 cases and 308,252 controls), celiac disease (3,690 cases and 361,055 controls), IBD (7,625 cases and 369,652 controls), psoriasis (9,267 cases and 364,071 controls), and anterior iridocyclitis (6,536 cases and 370,741 controls). The GWAS data for sarcoidosis (4,041 cases and 371,255 controls) and ADs were obtained from the FinnGen biobank (DF9 - May 11 2023) and are available at https://www.finngen.fi/en. All the analyzed data were categorical (qualitative) variables. The FinnGen study is an ongoing nationwide collection of residents in Finland genetic samples that combines genome information with digital healthcare and registry data (23).

### Instrument selection and data harmonization

SNPs with a significance level of *P* < 5 × 10^-8^ were identified and clumped based on linkage disequilibrium (r^2^ < 0.001) within a clumping distance of 10,000 kb. The 1000 Genomes European data was used as the reference panel for this process. In cases where instrumental SNPs for the exposure were not available in the outcome datasets, they were substituted with SNPs showing high linkage disequilibrium (r^2^ > 0.8) whenever possible. To ensure the alignment of beta values with the same alleles for the effects of SNPs on exposures and outcomes, harmonization was performed. The PhenoScanner (27, 28) database was utilized for manual screening and removal of SNPs associated with confounding factors and outcomes (*P*-value = 5 × 10^-8^, r^2^ = 0.8, Proxies = EUR, Build = 37). Additional information can be found in **Supplementary Table 3**. Outliers were identified using the MR-PRESSO method (29), and the data were reanalyzed after removing these outliers. The remaining SNPs were then used to conduct the MR study.

### Testing instrument strength and statistical power

The *F*-statistic for each SNP was calculated using the formula (30): Beta^2^/SE^2^, where Beta represents the estimated genetic effect and SE represents the standard error. Additionally, the proportion of variance (R^2^) explained by each SNP was calculated using the formula (31): 2 × EAF × (1–EAF) × Beta^2^, where EAF represents the effect allele frequency on exposures. The *F*-statistic is a measure of instrument strength, and a value greater than 10 is typically considered indicative of a sufficiently strong instrument (32).

### Statistical analyses

Our estimates are primarily based on the inverse variance weighted (IVW) analysis. The IVW method assumes the absence of horizontal pleiotropy for all SNPs and provides the most accurate assessment under this premise (33). In cases where heterogeneity exists, we employ the multiplicative random-effect IVW (IVW-MRE) model; otherwise, we use the fixed-effect IVW (IVW-FE) model. Additionally, we conducted sensitivity analyses using several other methods, including MR-Egger (34), Weighted median (35), Simple mode (36), and Weighted mode (36). To assess heterogeneity and evaluate the presence of horizontal pleiotropy, we performed various tests, such as the MR-Egger intercept test (37), Cochran’s Q test (38), and leave-one-out analyses (39). Finally, we performed the MR-Steiger directionality test to assess the correct direction of causality between the exposure and outcome variables (40).

All statistical analyses were performed using the “TwoSampleMR” (41) and “MRPRESSO” (29) packages in R (version 4.2.2). All reported *P*-values are bilateral, and a multiple-testing threshold of *P* < 6.25 × 10^-3^ (0.05/8) was applied to declare statistical significance using the Bonferroni method.

## Results

### Instrument statistics

For the bidirectional MR analysis of the relationships between sarcoidosis and ADs, the number of SNPs used as genetic instruments ranged from 3 (sarcoidosis) to 108 (autoimmune hypothyroidism), explaining 4.99 × 10^-4^ to 2.69 × 10^-2^ of the phenotypic variances. *F*-statistics for all diseases are ≥ 30, suggesting the good strength of genetic instruments (**Supplementary Tables 4**, **5**).

### Causal effects of ADs on sarcoidosis risk

**Figure 2:**
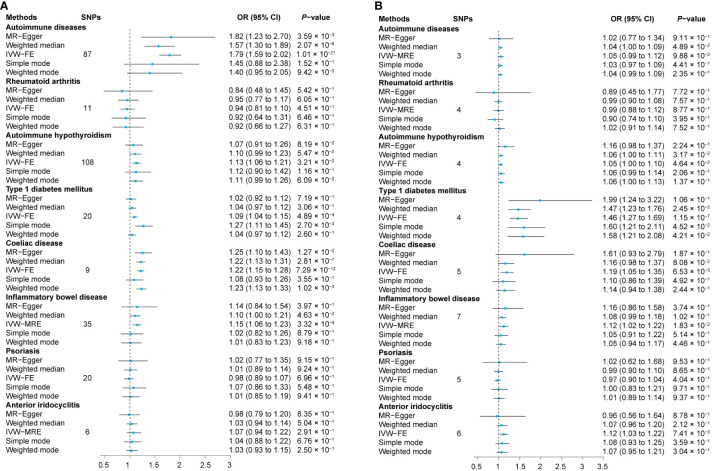
Relationship between exposures and outcomes. The MR analyses were conducted separately with sarcoidosis as both the outcome **(A)** and the exposure **(B)**. The dataset of autoimmune diseases analyzed in this study comprised a total of 44 different types of autoimmune-related diseases. SNPs, Single nucleotide polymorphisms; OR, Odds ratio; CI, Confidence intervals; IVW-MRE, Multiplicative random-effect inverse variance weighted; IVW-FE, Fixed-effect inverse variance weighted; MR, Mendelian randomization.

**Figure 3:**
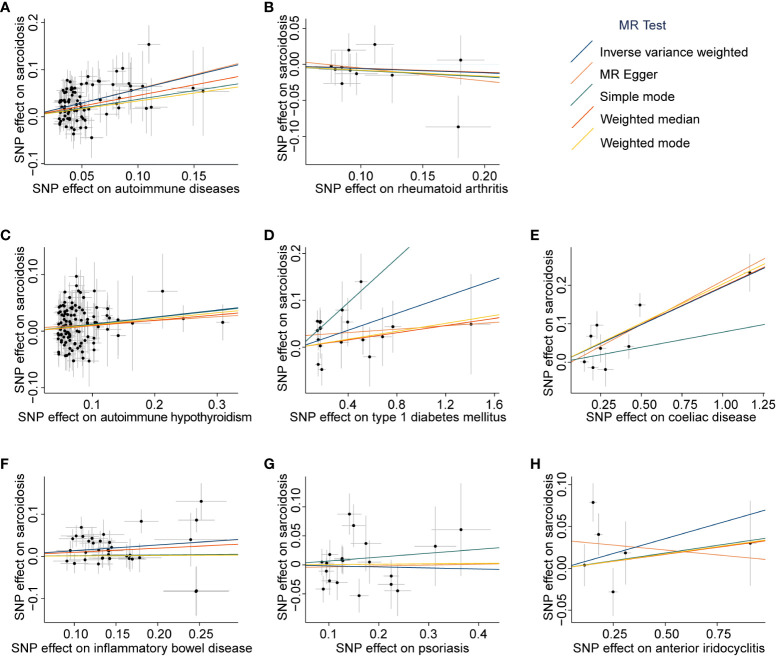
Scatter plots of associations between exposures-associated SNPs and sarcoidosis. The MR analyses were conducted with various exposures, including autoimmune diseases: autoimmune diseases **(A)**, rheumatoid arthritis **(B)**, autoimmune hypothyroidism **(C)**, type 1 diabetes mellitus **(D)**, coeliac disease **(E)**, inflammatory bowel disease **(F)**, psoriasis **(G)**, and anterior iridocyclitis **(H)**. The dataset of autoimmune diseases analyzed in this study comprised a total of 44 different types of autoimmune-related diseases. SNP, Single nucleotide polymorphism; MR, Mendelian randomization.

We first assessed the causal effect of ADs on sarcoidosis, and the results of the IVW-FE method showed that genetic predictors of ADs were significantly associated with a higher risk of sarcoidosis (odds ratio (OR) = 1.79, 95% confidence interval (CI) = 1.59 to 2.02, *P*_IVW-FE_ = 1.01 × 10^-21^). Additionally, the MR-Egger, and Weighted median methods yielded similar results (all *P* < 6.25 × 10^-3^, **Figure 2A**, **Supplementary Table 6**). The scatter plot and forest plot of associations between ADs-associated SNPs and sarcoidosis are presented in **Figure 3A** and **Supplementary Figure 1A**.

Subsequently, we assessed the causal effect of seven different types of autoimmune-related diseases on sarcoidosis. Among them, only genetic predictors of T1DM (OR = 1.09, 95% CI = 1.04 to 1.15, *P*_IVW-FE_ = 4.89 × 10^-4^), celiac disease (OR = 1.22, 95% CI = 1.15 to 1.28, *P*_IVW-FE_ = 7.29 × 10^-12^), and IBD (OR = 1.15, 95% CI = 1.06 to 1.23, *P*_IVW-MRE_ = 3.32 × 10^-4^) were associated with a higher risk of sarcoidosis (**Figure 2A**, **Supplementary Table 6**). Scatter plots are presented in **Figures 3B–H**, and the forest plots are shown in **Supplementary Figures 1B–H**.

Then, we performed sensitivity analyses to assess our results. The results of the MR-Egger regression and MR-PRESSO global test indicated that there was no overall horizontal pleiotropy in all IVs (all *P* > 0.05, **Table 1**). However, there was evidence of heterogeneity among the SNPs of IBD (*P*_MR-Egger_ = 0.049, *P*_IVW_ = 0.062) and anterior iridocyclitis (*P*_MR-Egger_ = 0.043, *P*_IVW_ = 0.023), as shown in **Table 1**. The results of the leave-one-out analysis and funnel plots are shown in **Supplementary Figures 2**, **3**. Finally, we found no evidence of reverse causality across the analyses in the MR Steiger test (all *P* < 0.001, **Supplementary Table 7**).

**Table 1:** Heterogeneity and pleiotropy analysis based on three different statistical methods.

Exposures	Outcomes	Cochran’s Q statistic				MR-PRESSO	MR-Egger intercept analysis
		MR-Egger		IVW			
		Q	*P*-value	Q	*P*-value	*P*-value	*P*-value
Autoimmune diseases	Sarcoidosis	104.783	0.072	104.793	0.082	0.107	0.928
Rheumatoid arthritis		6.826	0.655	7.022	0.723	0.715	0.668
Autoimmune hypothyroidism		128.738	0.066	129.431	0.069	0.106	0.452
Type 1 diabetes mellitus		21.907	0.236	25.856	0.134	0.159	0.088
Coeliac disease		13.867	0.054	14.504	0.070	0.138	0.588
Inflammatory bowel disease		47.529	0.049	47.529	0.062	0.062	0.984
Psoriasis		23.239	0.182	23.318	0.224	0.247	0.808
Anterior iridocyclitis		9.848	0.043	13.032	0.023	0.093	0.319
Sarcoidosis	Autoimmune diseases	5.522	0.019	5.805	0.055	/	0.858
	Rheumatoid arthritis	9.049	0.011	9.483	0.024	0.117	0.786
	Autoimmune hypothyroidism	0.712	0.700	2.270	0.518	0.601	0.338
	Type 1 diabetes mellitus	1.168	0.558	2.913	0.405	0.508	0.317
	Coeliac disease	6.240	0.101	8.933	0.063	0.136	0.338
	Inflammatory bowel disease	11.717	0.039	11.906	0.064	0.111	0.788
	Psoriasis	7.572	0.056	7.670	0.104	0.162	0.856
	Anterior iridocyclitis	8.361	0.079	9.095	0.105	0.134	0.586

The dataset of autoimmune diseases analyzed in this study comprised a total of 44 different types of autoimmune-related diseases. MR, Mendelian randomization; IVW, Inverse variance weighted.

### Causal effects of sarcoidosis on ADs risk

**Figure 4:**
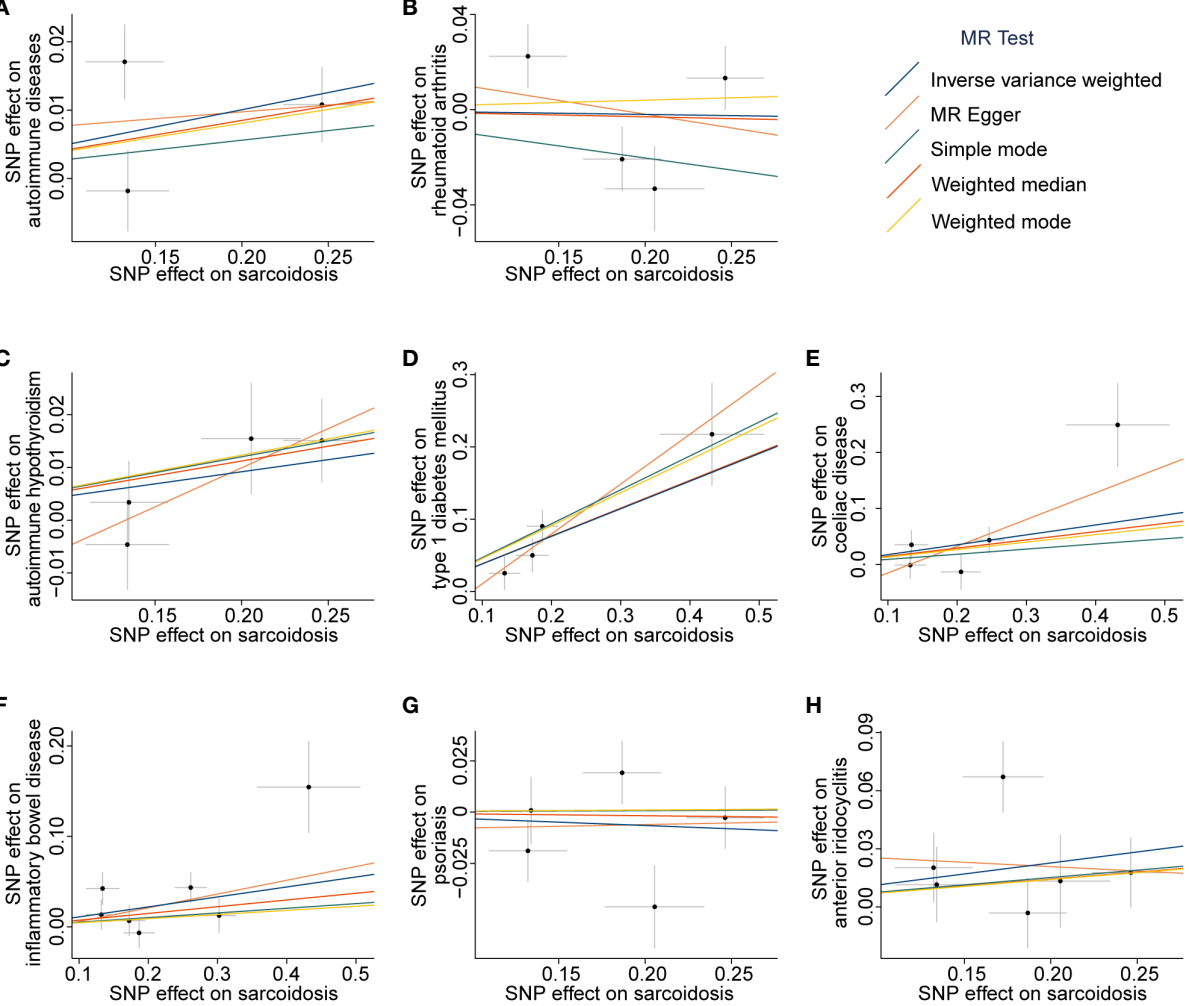
Scatter plots of associations between sarcoidosis-associated SNPs and outcomes. The MR analyses were conducted with the following outcomes: autoimmune diseases **(A)**, rheumatoid arthritis **(B)**, autoimmune hypothyroidism **(C)**, type 1 diabetes mellitus **(D)**, coeliac disease **(E)**, inflammatory bowel disease **(F)**, psoriasis **(G)**, and anterior iridocyclitis **(H)**. The dataset of autoimmune diseases analyzed in this study comprised a total of 44 different types of autoimmune-related diseases. SNP, Single nucleotide polymorphism; MR, Mendelian randomization.

The results of the IVW method showed that there is no causal effect of genetic predictors of sarcoidosis on the risk of ADs (OR = 1.05, 95% CI 0.99 to 1.12, *P*_IVW-MRE_ = 9.88 × 10^-2^), and these results were validated by MR-Egger, weighted median, simple mode, and weighted mode (all *P* > 6.25 × 10^-3^, **Figure 2B** and **Supplementary Table 6**). The scatter plot and forest plot of associations between sarcoidosis-associated SNPs and ADs are presented in **Figure 4A** and **Supplementary Figure 4A**.

Among the tested seven different types of autoimmune-related diseases, genetic predictors of sarcoidosis were only associated with a higher risk of T1DM (OR = 1.46, 95% CI 1.27 to 1.69, *P*_IVW-FE_ = 1.15 × 10^-7^, **Figure 2B** and **Supplementary Table 6**). Scatter plots are presented in **Figures 4B–H**, and the forest plots are shown in **Supplementary Figures 4B–H**.

There was also no overall horizontal pleiotropy in all IVs according to the results of the MR-Egger regression and MR-PRESSO global test (all *P* > 0.05, **Table 1**). However, partial heterogeneity existed among the SNPs, as indicated by Cochran’s Q test (**Table 1**). The results of the leave-one-out analysis and funnel plots are shown in **Supplementary Figures 5**, **6**. Lastly, there is no evidence of reverse causality across the analyses based on the MR Steiger test (all *P* < 0.001, **Supplementary Table 7**).

## Discussion

In this study, we utilized genetic variants as unconfounded proxies for sarcoidosis and ADs to explore their causal relationship in a bidirectional MR study. The results demonstrated that genetic predictors of ADs were associated with an elevated risk of developing sarcoidosis. However, we did not find evidence supporting the notion that genetic predictors of sarcoidosis are linked to an increased risk of ADs. Moreover, the robustness of our findings was confirmed through various sensitivity analyses conducted throughout the study.

Previous studies have reported a significant association between ADs and sarcoidosis, with OR higher than 5 for specific ADs such as chronic active hepatitis, systemic lupus erythematosus, and sjögren syndrome (42–44). This close relationship suggests a potential shared immunopathogenic mechanism between sarcoidosis and ADs. Kaiser et al. (45) proposed the classification of sarcoidosis as an autoimmune spectrum disorder, although the supporting evidence remains predominantly indirect, and the cause-effect relationship between sarcoidosis and ADs is still not fully understood. In our study, we identified 87 common variants associated with ADs (including 44 different types of autoimmune-related diseases) through GWAS, and further confirmed the significant role of ADs in the development of sarcoidosis from a genetic perspective. It should be noted that previous studies have reported a relatively low percentage (11.5%-17.6%) of patients with sarcoidosis also having coexisting ADs (42–44). Therefore, the pathogenesis of sarcoidosis cannot be solely attributed to immune dysregulation, and other factors such as genetic or environmental exposures may also contribute (1). Additionally, ADs themselves may increase the risk of developing sarcoidosis after exposure to environmental triggers.

The etiology of sarcoidosis is considered multifactorial (5). Previous studies have proposed that sarcoidosis pathogenesis involves a dysregulated immune system influenced by both environmental and genetic factors, although the precise mechanisms remain incompletely understood (5). T lymphocytes, especially CD4^+^ T cells, have been implicated in the development of sarcoidosis (45) and other diseases such as gastrointestinal diseases (e.g., celiac disease, IBD), endocrine diseases (e.g., T1DM), liver diseases (e.g., primary biliary cholangitis), neurological diseases (e.g., multiple sclerosis), and cutaneous diseases (e.g., psoriasis) (46–48). This may explain the frequent co-occurrence of ADs with sarcoidosis. In fact, subgroup analyses in our study also revealed that genetic predictors of T1DM, celiac disease, and IBD were causally linked to an elevated risk of sarcoidosis. This indirectly further validates the frequent coexistence of autoimmune diseases and sarcoidosis, shedding light on the reasons behind the association between ADs and sarcoidosis. Recent studies have also highlighted the involvement of other cell types, including dendritic cells, CD8^+^ cytotoxic T cells, B cells, natural killer cells, and NKT cells, in the pathogenesis of sarcoidosis. However, further research is necessary to elucidate the precise mechanisms underlying their actions (45).

In the present study, we did not find an association between genetic predictors of sarcoidosis and an increased risk of ADs. This finding may be explained by the underlying pathogenesis of sarcoidosis. Sarcoidosis is often linked to environmental exposures and has the potential to resolve spontaneously after the cessation of these exposures (1). On the other hand, ADs are characterized by persistent immune responses against self-antigens, resulting in tissue damage (49). Therefore, the cause-effect relationship between sarcoidosis and ADs may be unidirectional, with sarcoidosis not directly contributing to the development of ADs. However, in our MR analysis, we did identify bi-directional positive causal relationships between sarcoidosis and T1DM. This finding suggests potential differences or nuances between various ADs, highlighting the need for further research to validate and comprehend the underlying reasons for these distinctions.

The primary strength of this study lies in the application of MR studies to establish causal relationships, particularly in the investigation of rare diseases such as sarcoidosis. Large prospective studies with multicenter cohorts are often challenging for rare diseases due to limited sample sizes. MR analysis, employing genetic variants as IVs, helps address potential biases encountered in traditional epidemiological studies, including confounding, selection biases, recall biases, and reverse causality (20–22). Furthermore, the use of data from the FinnGen study constitutes a crucial strength in our research. Leveraging data from a single large-scale study helps minimize bias associated with ethnic differences. This approach reduces potential confounding factors related to population diversity, thereby enhancing the reliability and generalizability of our findings.

There are several limitations in this study that should be acknowledged. First, the data used in the study were derived from the FinnGen study, which includes residents in Finland. This may limit the generalizability of the findings to other patient populations (e.g., North Americans, Australians, or Asians), as genetic and environmental factors can vary across different populations. Additionally, there is a potential bias in MR studies due to sample overlap (ranging from 0.51% to 2.71%), which could impact the accuracy of the results. Second, this study is primarily limited by the data source itself, as there was no opportunity to perform external validation using an independent cohort. Third, due to the small sample size of the FinnGen study, it was not possible to investigate the associations between each autoimmune disease and sarcoidosis individually. Instead, a pooled sample of different ADs was used to reduce bias and statistical errors associated with small sample sizes. However, this approach may not capture potential differences or nuances between different ADs. Fourth, we identified a limited number of significantly associated loci for sarcoidosis compared to ADs. The smaller sample size in sarcoidosis may account for the limited number of significant loci, which could explain the negative result in the MR analysis from sarcoidosis to ADs. Lastly, the GWAS used in this study did not account for the diversity of sarcoidosis, such as Löfgren syndrome.

## Conclusion

In conclusion, this study employed a large GWAS to perform a MR investigation into the potential causal relationship between sarcoidosis and ADs. The results provided robust genetic evidence supporting a significant causal effect of genetic predictors of ADs, such as T1DM, celiac disease, and IBD, on the development of sarcoidosis. Conversely, genetic predictors of sarcoidosis were not found to be linked to ADs. These findings provide potential insights into the underlying autoimmune mechanisms of sarcoidosis.

## Data availability statement

The original contributions presented in the study are included in the article/**Supplementary Material**. Further inquiries can be directed to the corresponding author.

## Ethics statement

Ethical approval was not required for the study involving humans in accordance with the local legislation and institutional requirements. Written informed consent to participate in this study was not required from the participants or the participants’ legal guardians/next of kin in accordance with the national legislation and the institutional requirements.

## Author contributions

DS: Data curation, Formal Analysis, Methodology, Writing – original draft. RM: Data curation, Formal Analysis, Writing – review & editing. JW: Data curation, Writing – review & editing. YW: Data curation, Writing – review & editing. QY: Funding acquisition, Methodology, Resources, Supervision, Validation, Writing – review & editing.

## Supplementary material

Supplementary Figure 1 Forest plot for the causal effect of each exposures-associated SNP on sarcoidosis risk. The MR analyses were conducted with various exposures, including autoimmune diseases **(A)**, rheumatoid arthritis **(B)**, autoimmune hypothyroidism **(C)**, type 1 diabetes mellitus **(D)**, coeliac disease **(E)**, inflammatory bowel disease **(F)**, psoriasis **(G)**, and anterior iridocyclitis **(H)**. The dataset of autoimmune diseases analyzed in this study comprised a total of 44 different types of autoimmune-related diseases. SNP, Single nucleotide polymorphism; MR, Mendelian randomization.

Supplementary Figure 2 Leave-one-out analyses for SNPs associated with exposures on sarcoidosis. The MR analyses were conducted with various exposures, including: autoimmune diseases **(A)**, rheumatoid arthritis **(B)**, autoimmune hypothyroidism **(C)**, type 1 diabetes mellitus **(D)**, coeliac disease **(E)**, inflammatory bowel disease **(F)**, psoriasis **(G)**, and anterior iridocyclitis **(H)**. The dataset of autoimmune diseases analyzed in this study comprised a total of 44 different types of autoimmune-related diseases. MR, Mendelian randomization; SNPs, Single nucleotide polymorphisms.

Supplementary Figure 3 Funnel plots for overall heterogeneity in the effect of exposures on sarcoidosis. The MR analyses were conducted with various exposures, including autoimmune diseases **(A)**, rheumatoid arthritis **(B)**, autoimmune hypothyroidism **(C)**, type 1 diabetes mellitus **(D)**, coeliac disease **(E)**, inflammatory bowel disease **(F)**, psoriasis **(G)**, and anterior iridocyclitis **(H)**. The dataset of autoimmune diseases analyzed in this study comprised a total of 44 different types of autoimmune-related diseases. MR, Mendelian randomization; SE, Standard error; IV, Instrumental variable.

Supplementary Figure 4 Forest plots for the causal effect of each sarcoidosis-associated SNP on outcomes risk. The MR analyses were conducted with the following outcomes: autoimmune diseases **(A)**, rheumatoid arthritis **(B)**, autoimmune hypothyroidism **(C)**, type 1 diabetes mellitus **(D)**, coeliac disease **(E)**, inflammatory bowel disease **(F)**, psoriasis **(G)**, and anterior iridocyclitis **(H)**. The dataset of autoimmune diseases analyzed in this study comprised a total of 44 different types of autoimmune-related diseases. SNP, Single nucleotide polymorphism; MR, Mendelian randomization.

Supplementary Figure 5 Leave-one-out analyses for SNPs associated with sarcoidosis on outcomes. The MR analyses were conducted with the following outcomes: autoimmune diseases **(A)**, rheumatoid arthritis **(B)**, autoimmune hypothyroidism **(C)**, type 1 diabetes mellitus **(D)**, coeliac disease **(E)**, inflammatory bowel disease **(F)**, psoriasis **(G)**, and anterior iridocyclitis **(H)**. The dataset of autoimmune diseases analyzed in this study comprised a total of 44 different types of autoimmune-related diseases. MR, Mendelian randomization; SNPs, Single nucleotide polymorphisms.

Supplementary Figure 6 Funnel plots for overall heterogeneity in the effect of sarcoidosis on outcomes. The MR analyses were conducted with the following outcomes: autoimmune diseases **(A)**, rheumatoid arthritis **(B)**, autoimmune hypothyroidism **(C)**, type 1 diabetes mellitus **(D)**, coeliac disease **(E)**, inflammatory bowel disease **(F)**, psoriasis **(G)**, and anterior iridocyclitis **(H)**. The dataset of autoimmune diseases analyzed in this study comprised a total of 44 different types of autoimmune-related diseases. MR, Mendelian randomization; SE, Standard error; IV, Instrumental variable.
