# Supplementary figures and images for: The causal relationship between sarcoidosis and autoimmune diseases: a bidirectional Mendelian randomization study in FinnGen
Source: Front Immunol. 2024 Apr 22;15:1325127. doi: 10.3389/fimmu.2024.1325127 (PMC11070530; doi:10.3389/fimmu.2024.1325127)

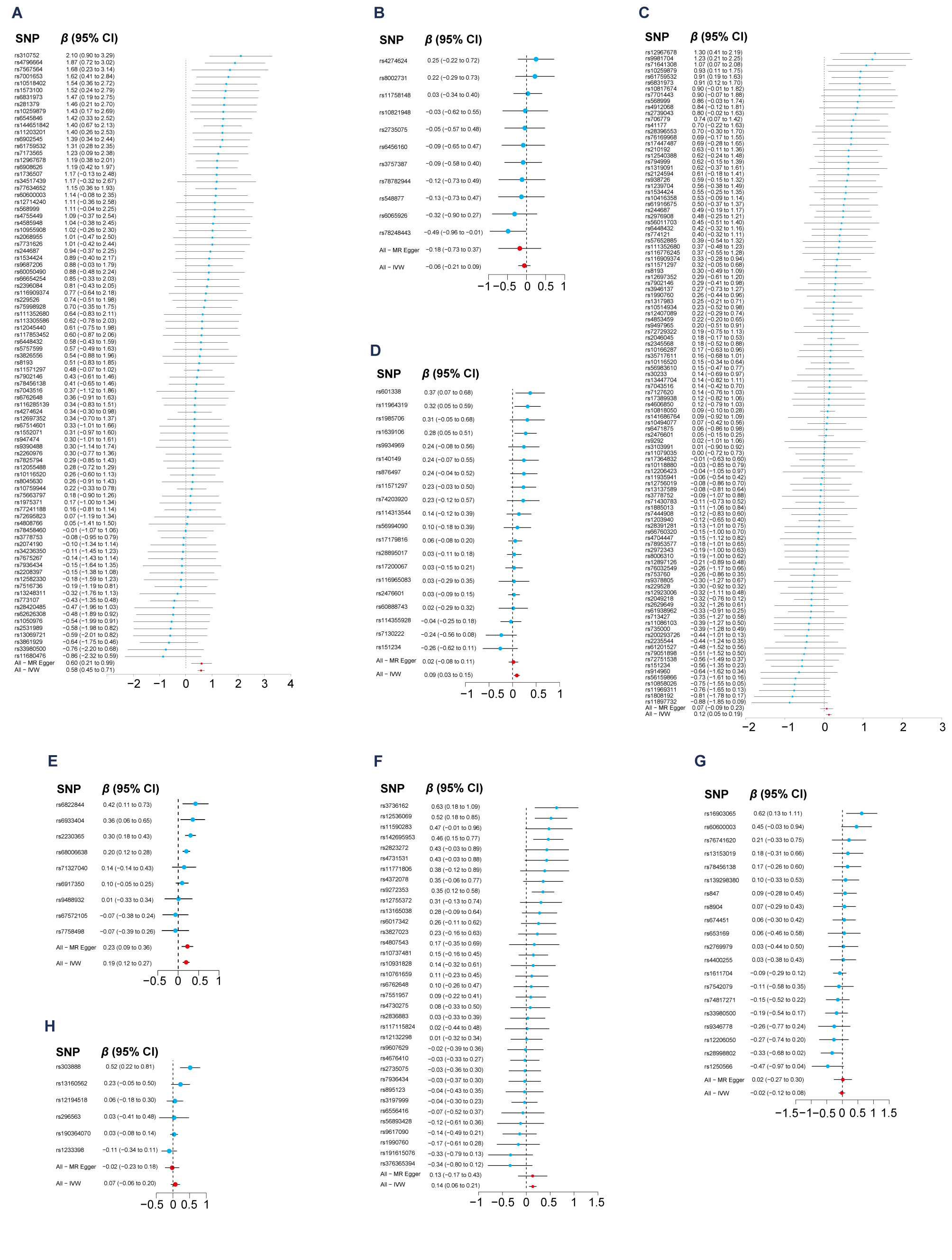

Supplement: Supplementary Figure 1 — Forest plot for the causal effect of each exposures-associated SNP on sarcoidosis risk. The MR analyses were conducted with various exposures, including autoimmune diseases (A), rheumatoid arthritis (B), autoimmune hypothyroidism (C), type 1 diabetes mellitus (D), coeliac disease (E), inflammatory bowel disease (F), psoriasis (G), and anterior iridocyclitis (H). The dataset of autoimmune diseases analyzed in this study comprised a total of 44 different types of autoimmune-related diseases. SNP, Single nucleotide polymorphism; MR, Mendelian randomization. [file Image_1.tif]

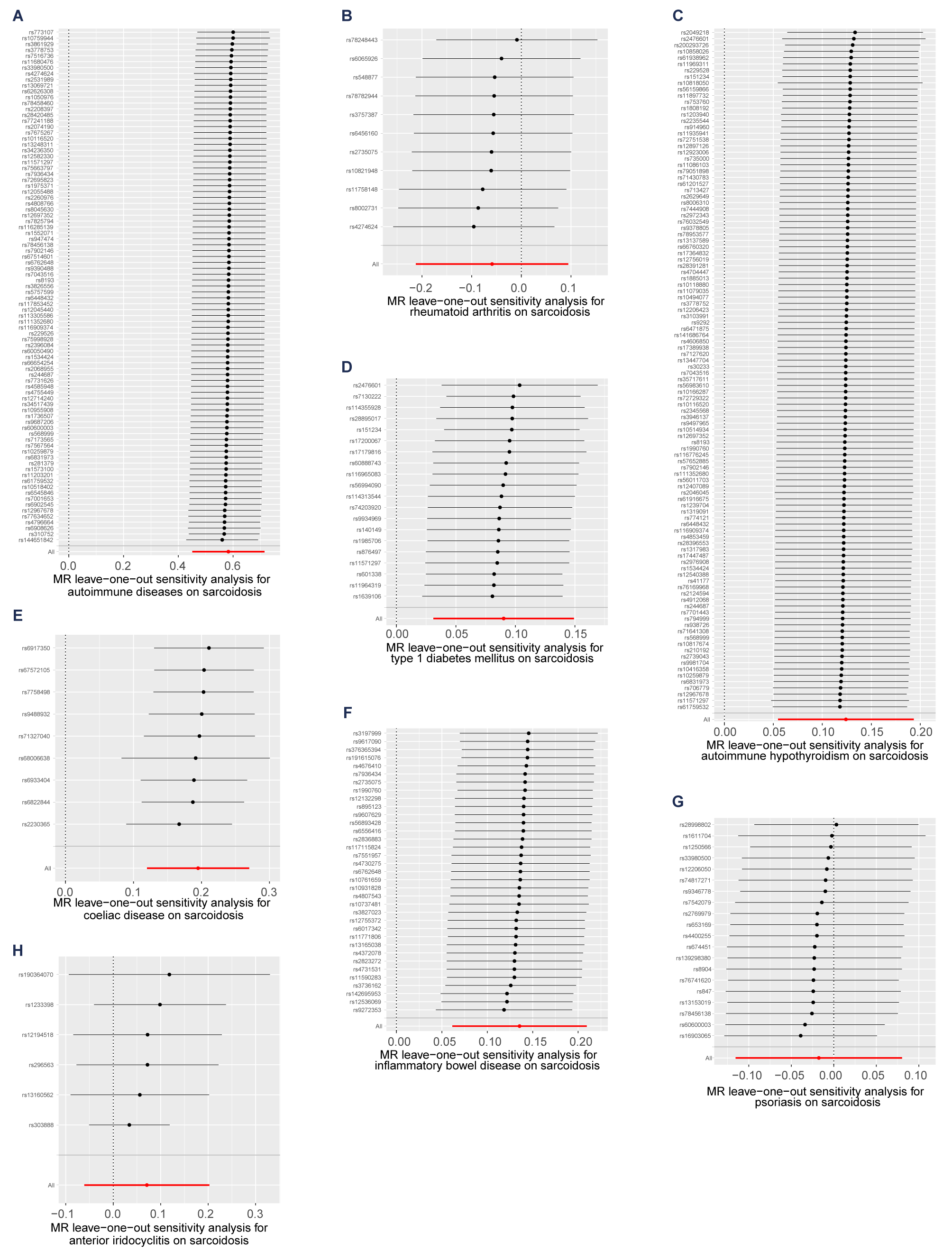

Supplement: Supplementary Figure 2 — Leave-one-out analyses for SNPs associated with exposures on sarcoidosis. The MR analyses were conducted with various exposures, including: autoimmune diseases (A), rheumatoid arthritis (B), autoimmune hypothyroidism (C), type 1 diabetes mellitus (D), coeliac disease (E), inflammatory bowel disease (F), psoriasis (G), and anterior iridocyclitis (H). The dataset of autoimmune diseases analyzed in this study comprised a total of 44 different types of autoimmune-related diseases. MR, Mendelian randomization; SNPs, Single nucleotide polymorphisms. [file Image_2.tif]

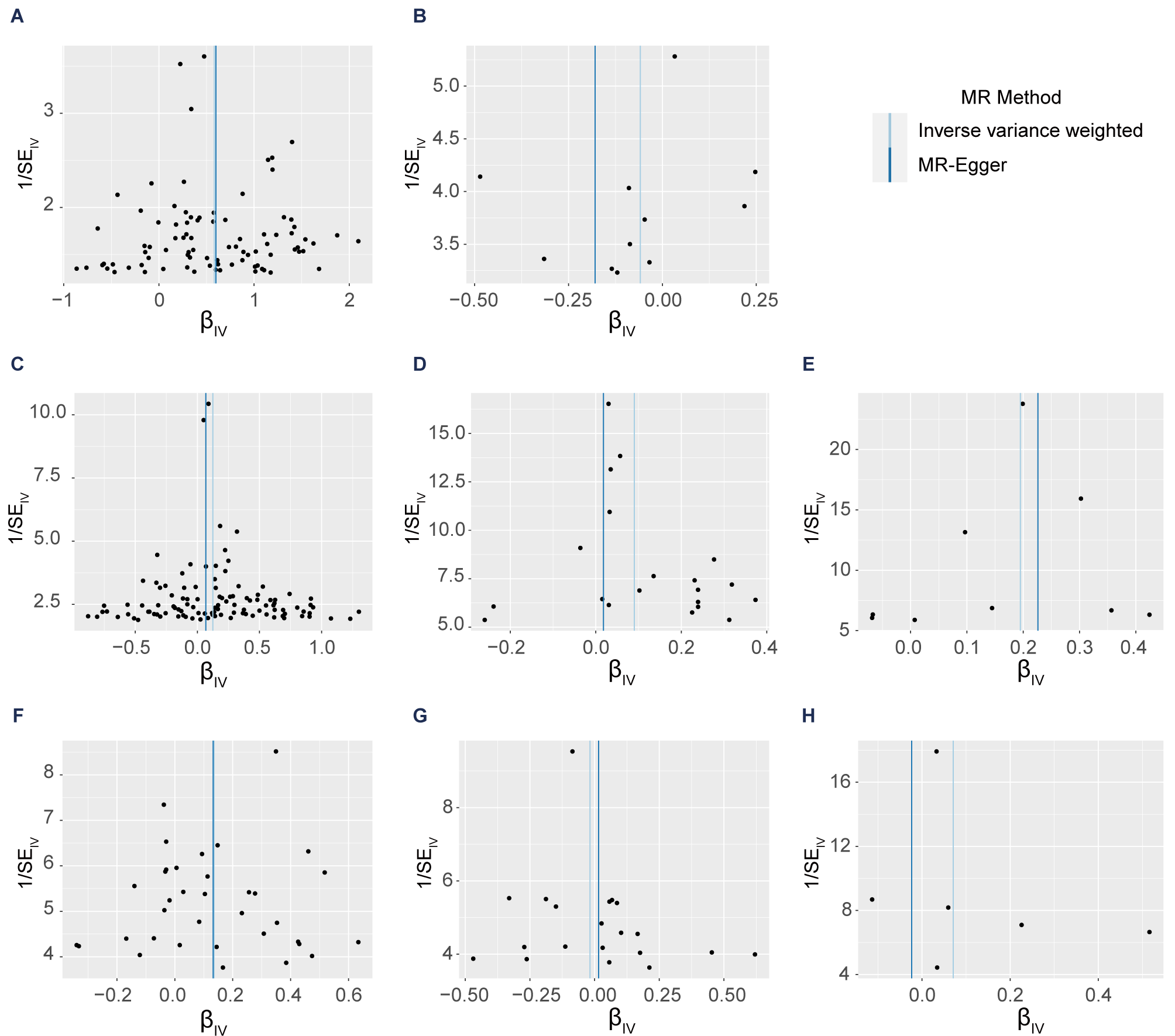

Supplement: Supplementary Figure 3 — Funnel plots for overall heterogeneity in the effect of exposures on sarcoidosis. The MR analyses were conducted with various exposures, including autoimmune diseases (A), rheumatoid arthritis (B), autoimmune hypothyroidism (C), type 1 diabetes mellitus (D), coeliac disease (E), inflammatory bowel disease (F), psoriasis (G), and anterior iridocyclitis (H). The dataset of autoimmune diseases analyzed in this study comprised a total of 44 different types of autoimmune-related diseases. MR, Mendelian randomization; SE, Standard error; IV, Instrumental variable. [file Image_3.tif]

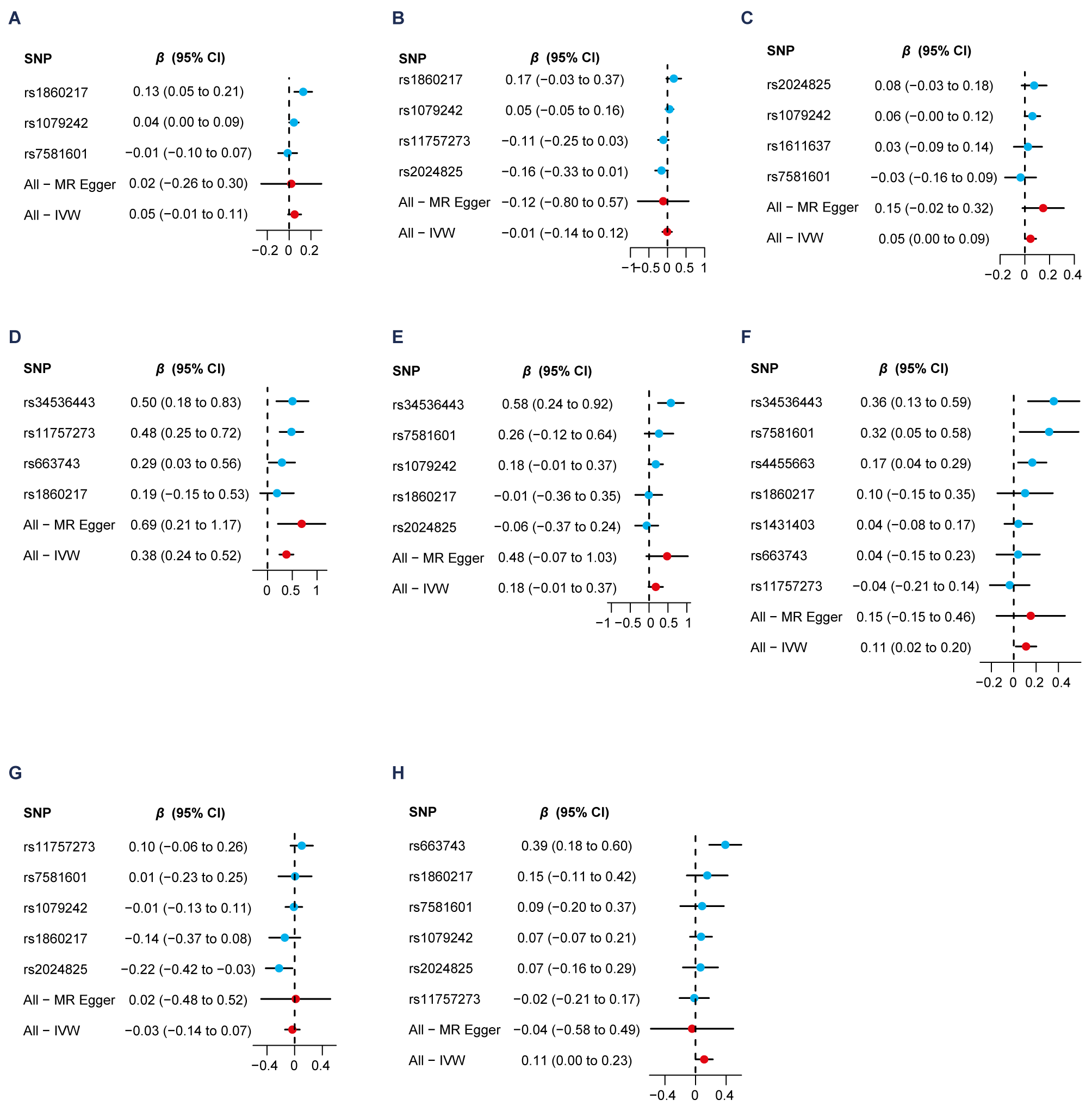

Supplement: Supplementary Figure 4 — Forest plots for the causal effect of each sarcoidosis-associated SNP on outcomes risk. The MR analyses were conducted with the following outcomes: autoimmune diseases (A), rheumatoid arthritis (B), autoimmune hypothyroidism (C), type 1 diabetes mellitus (D), coeliac disease (E), inflammatory bowel disease (F), psoriasis (G), and anterior iridocyclitis (H). The dataset of autoimmune diseases analyzed in this study comprised a total of 44 different types of autoimmune-related diseases. SNP, Single nucleotide polymorphism; MR, Mendelian randomization. [file Image_4.tif]

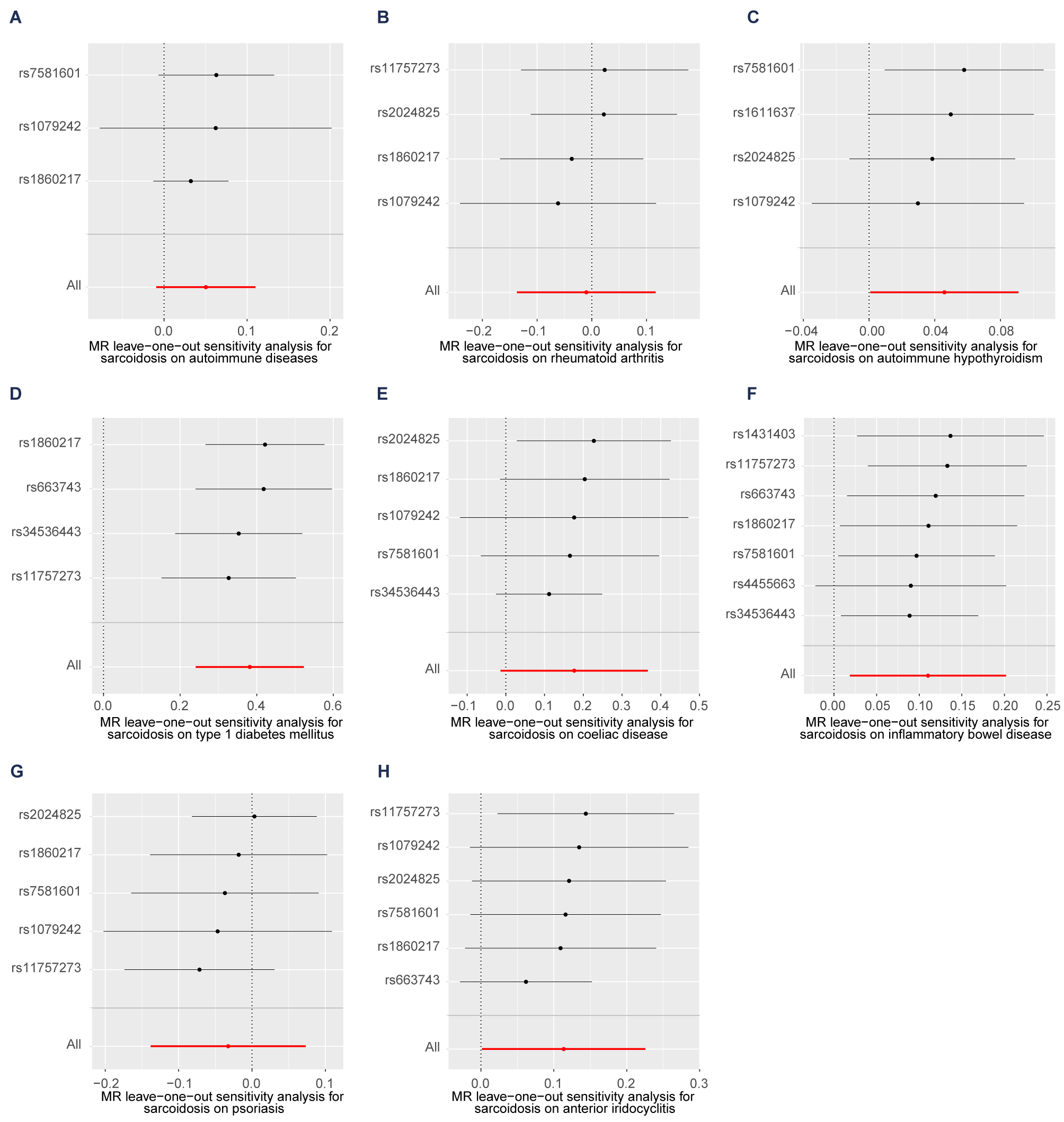

Supplement: Supplementary Figure 5 — Leave-one-out analyses for SNPs associated with sarcoidosis on outcomes. The MR analyses were conducted with the following outcomes: autoimmune diseases (A), rheumatoid arthritis (B), autoimmune hypothyroidism (C), type 1 diabetes mellitus (D), coeliac disease (E), inflammatory bowel disease (F), psoriasis (G), and anterior iridocyclitis (H). The dataset of autoimmune diseases analyzed in this study comprised a total of 44 different types of autoimmune-related diseases. MR, Mendelian randomization; SNPs, Single nucleotide polymorphisms. [file Image_5.tif]

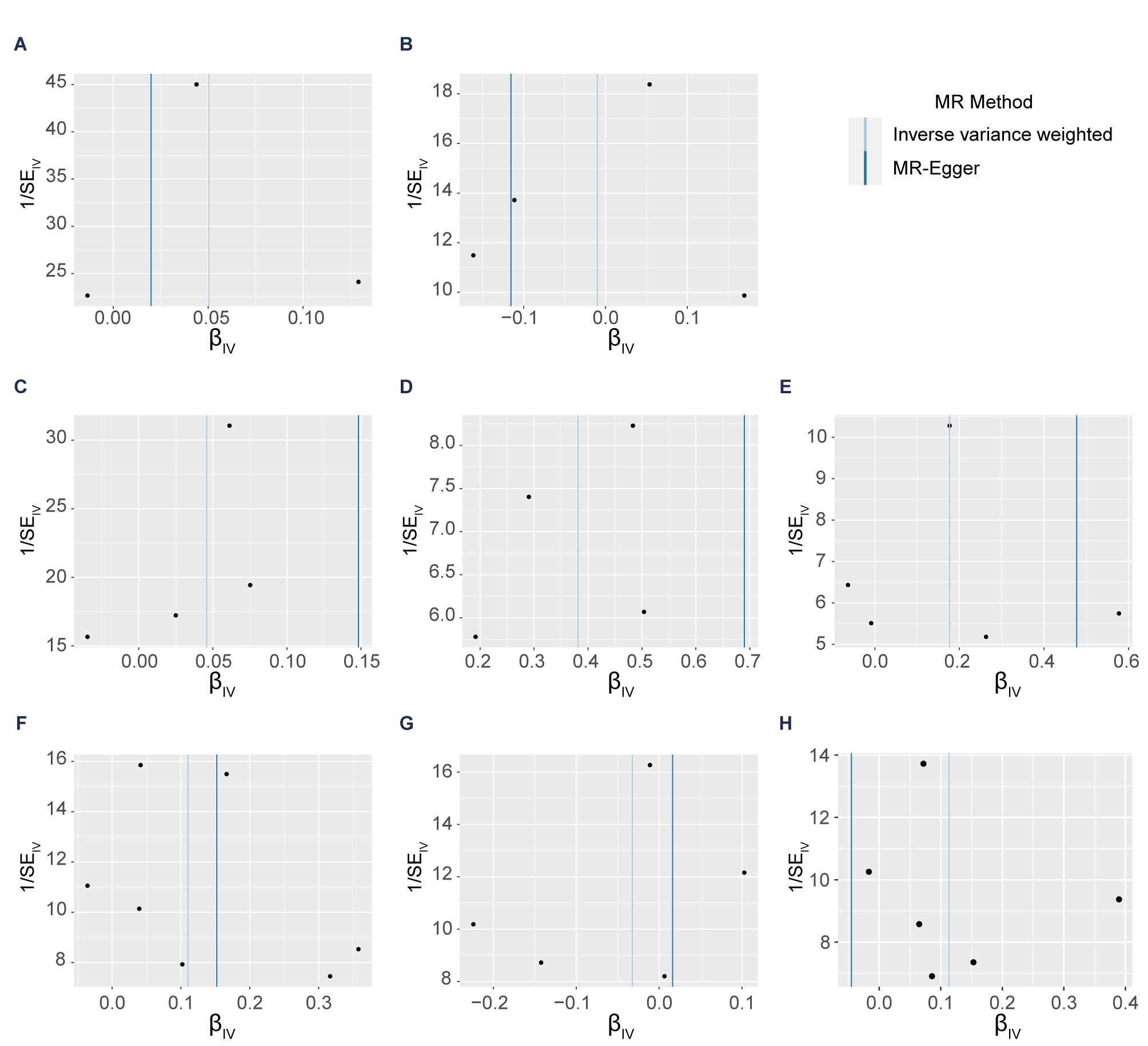

Supplement: Supplementary Figure 6 — Funnel plots for overall heterogeneity in the effect of sarcoidosis on outcomes. The MR analyses were conducted with the following outcomes: autoimmune diseases (A), rheumatoid arthritis (B), autoimmune hypothyroidism (C), type 1 diabetes mellitus (D), coeliac disease (E), inflammatory bowel disease (F), psoriasis (G), and anterior iridocyclitis (H). The dataset of autoimmune diseases analyzed in this study comprised a total of 44 different types of autoimmune-related diseases. MR, Mendelian randomization; SE, Standard error; IV, Instrumental variable. [file Image_6.tif]
