# Supplementary material for: The causal relationship between sarcoidosis and autoimmune diseases: a bidirectional Mendelian randomization study in FinnGen
Source: Front Immunol. 2024 Apr 22;15:1325127. doi: 10.3389/fimmu.2024.1325127 (PMC11070530; doi:10.3389/fimmu.2024.1325127)
Supplement: Supplementary file 7 [file DataSheet_1.pdf]

STROBE-MR checklist of recommended items to address in reports of Mendelian randomization studies[1 2]

Item No.	Section		Checklist item	Page No.	Relevant text from manuscript
1	**TITLE and ABSTRACT**		Indicate Mendelian randomization (MR) as the study's design in the title and/or the abstract if that is a main purpose of the study	01	The causal relationship between sarcoidosis and autoimmune diseases: a bidirectional Mendelian randomization study in FinnGen
	**INTRODUCTION**				
2	**Background**		Explain the scientific background and rationale for the reported study. What is the exposure? Is a potential causal relationship between exposure and outcome plausible? Justify why MR is a helpful method to address the study question	02	However, all the associations between sarcoidosis and ADs mentioned above were derived from cross-sectional studies, leaving the causal nature of these connections elusive. Establishing causal relationships not only deepens the understanding of sarcoidosis and ADs pathogenesis but also has the potential to guide pathogenesis-oriented interventions against sarcoidosis and ADs in clinical settings.
3	**Objectives**		State specific objectives clearly, including pre-specified causal hypotheses (if any). State that MR is a method that, under specific assumptions, intends to estimate causal effects	02	Therefore, in this study, we performed a systematic bidirectional MR analysis to investigate the causal relationship between sarcoidosis and ADs.
	**METHODS**				
4	**Study design and data sources**		Present key elements of the study design early in the article. Consider including a table listing sources of data for all phases of the study. For each data source contributing to the analysis, describe the following:		
		a)	Setting: Describe the study design and the underlying population, if possible. Describe the setting, locations, and relevant dates, including periods of recruitment, exposure, follow-up, and data collection, when available.	02	Study design
		b)	Participants: Give the eligibility criteria, and the sources and methods of selection of participants. Report the sample size, and whether any power or sample size calculations were carried out prior to the main analysis	02	Currently, there is no specialized GWAS data specifically dedicated to ADs available globally. Therefore, we selected a dataset consisting of 96,150 cases and 281,127 controls to encompass a wide range of ADs, including 44 different types of autoimmune-related diseases (Supplementary Table 1). We identified diseases within the dataset that had a case size exceeding 3,500 cases and conducted subgroup analyses specifically for these diseases. The diseases included in the subgroup analyses, along with their respective case and control sizes, are as follows (Supplementary Table

					2): rheumatoid arthritis (12,555 cases and 240,862 controls), autoimmune hypothyroidism (40,926 cases and 274,069 controls), T1DM (4,196 cases and 308,252 controls), celiac disease (3,690 cases and 361,055 controls), IBD (7,625 cases and 369,652 controls), psoriasis (9,267 cases and 364,071 controls), and anterior iridocyclitis (6,536 cases and 370,741 controls). The GWAS data for sarcoidosis (4,041 cases and 371,255 controls) and ADs were obtained from the FinnGen biobank (DF9 - May 11 2023) and are available at https://www.finngen.fi/en.
		c)	Describe measurement, quality control and selection of genetic variants	02	Instrument selection and data harmonization
		d)	For each exposure, outcome, and other relevant variables, describe methods of assessment and diagnostic criteria for diseases	02	We identified diseases within the dataset that had a case size exceeding 3,500 cases and conducted subgroup analyses specifically for these diseases. The diseases included in the subgroup analyses, along with their respective case and control sizes, are as follows (Supplementary Table 2): rheumatoid arthritis (12,555 cases and 240,862 controls), autoimmune hypothyroidism (40,926 cases and 274,069 controls), T1DM (4,196 cases and 308,252 controls), celiac disease (3,690 cases and 361,055 controls), IBD (7,625 cases and 369,652 controls), psoriasis (9,267 cases and 364,071 controls), and anterior iridocyclitis (6,536 cases and 370,741 controls). The GWAS data for sarcoidosis (4,041 cases and 371,255 controls) and ADs were obtained from the FinnGen biobank (DF9 - May 11 2023) and are available at https://www.finngen.fi/en. All the analyzed data were categorical (qualitative) variables. The FinnGen study is an ongoing nationwide collection of residents in Finland genetic samples that combines genome information with digital healthcare and registry data.
		e)	Provide details of ethics committee approval and participant informed consent, if relevant		Not applicable
5	**Assumptions**		Explicitly state the three core IV assumptions for the main analysis (relevance, independence and exclusion restriction) as well assumptions for any additional or sensitivity analysis	02	To satisfy the assumptions of MR, these IVs must satisfy three key criteria: (1) strong associations with the exposure of interest, (2) lack of association with confounding factors, and (3) absence of direct

					influence on the outcome apart from the exposure (Figure 1).
6	**Statistical methods: main analysis**		Describe statistical methods and statistics used		
		a)	Describe how quantitative variables were handled in the analyses (i.e., scale, units, model)	02	All the analyzed data were categorical (qualitative) variables.
		b)	Describe how genetic variants were handled in the analyses and, if applicable, how their weights were selected	02	SNPs with a significance level of $P < 5 \times 10^{-8}$ were identified and clumped based on linkage disequilibrium ($r^2 < 0.001$) within a clumping distance of 10,000 kb. The 1000 Genomes European data was used as the reference panel for this process. In cases where instrumental SNPs for the exposure were not available in the outcome datasets, they were substituted with SNPs showing high linkage disequilibrium ($r^2 > 0.8$) whenever possible. To ensure the alignment of beta values with the same alleles for the effects of SNPs on exposures and outcomes, harmonization was performed. The PhenoScanner database was utilized for manual screening and removal of SNPs associated with confounding factors and outcomes (P-value = 5×10^{-8}, $r^2 = 0.8$, Proxies = EUR, Build = 37).
		c)	Describe the MR estimator (e.g. two-stage least squares, Wald ratio) and related statistics. Detail the included covariates and, in case of two-sample MR, whether the same covariate set was used for adjustment in the two samples	03	Our estimates are primarily based on the inverse variance weighted (IVW) analysis.
		d)	Explain how missing data were addressed	02	In cases where instrumental SNPs for the exposure were not available in the outcome datasets, they were substituted with SNPs showing high linkage disequilibrium ($r^2 > 0.8$) whenever possible.
		e)	If applicable, indicate how multiple testing was addressed		Not applicable
7	**Assessment of assumptions**		Describe any methods or prior knowledge used to assess the assumptions or justify their validity	03	The *F*-statistic for each SNP was calculated using the formula: $Beta^2/SE^2$, where Beta represents the estimated genetic effect and SE represents the standard error. Additionally, the proportion of variance (R^2) explained by each SNP was calculated using the formula: $2 \times EAF \times (1–EAF) \times Beta^2$, where EAF represents the effect allele frequency on exposures. The F-statistic is a measure of instrument strength, and a value

				greater than 10 is typically considered indicative of a sufficiently strong instrument.
8	**Sensitivity analyses and additional analyses**	Describe any sensitivity analyses or additional analyses performed (e.g. comparison of effect estimates from different approaches, independent replication, bias analytic techniques, validation of instruments, simulations)	03	Additionally, we conducted sensitivity analyses using several other methods, including MR-Egger, Weighted median, Simple mode, and Weighted mode.
9	**Software and pre-registration**			
	a)	Name statistical software and package(s), including version and settings used	03	All statistical analyses were performed using the “TwoSampleMR” and “MRPRESSO” packages in R (version 4.2.2).
	b)	State whether the study protocol and details were pre-registered (as well as when and where)		N/A
	**RESULTS**			
10	**Descriptive data**			
	a)	Report the numbers of individuals at each stage of included studies and reasons for exclusion. Consider use of a flow diagram	02	Therefore, we selected a dataset consisting of 96,150 cases and 281,127 controls to encompass a wide range of ADs, including 44 different types of autoimmune-related diseases (Supplementary Table 1). We identified diseases within the dataset that had a case size exceeding 3,500 cases and conducted subgroup analyses specifically for these diseases.
	b)	Report summary statistics for phenotypic exposure(s), outcome(s), and other relevant variables (e.g. means, SDs, proportions)	02	The diseases included in the subgroup analyses, along with their respective case and control sizes, are as follows (Supplementary Table 2)
	c)	If the data sources include meta-analyses of previous studies, provide the assessments of heterogeneity across these studies		Not applicable
	d)	For two-sample MR:<br>i. Provide justification of the similarity of the genetic variant-exposure associations between the exposure and outcome samples<br>ii. Provide information on the number of individuals who overlap between the exposure and outcome studies	02-03	i. For the bidirectional MR analysis of the relationships between sarcoidosis and ADs, the number of SNPs used as genetic instruments ranged from 3 (sarcoidosis) to 108 (autoimmune hypothyroidism), explaining 4.99×10^{-4} to 2.69×10^{-2} of the phenotypic variances. F-statistics for all diseases are ≥ 30, suggesting the good strength of genetic instruments (Supplementary Tables 4-5).<br>ii. Supplementary Table 2

11	**Main results**				
		a)	Report the associations between genetic variant and exposure, and between genetic variant and outcome, preferably on an interpretable scale	03-04	We first assessed the causal effect of ADs on sarcoidosis, and the results of the IVW-FE method showed that genetic predictors of ADs were significantly associated with a higher risk of sarcoidosis
		b)	Report MR estimates of the relationship between exposure and outcome, and the measures of uncertainty from the MR analysis, on an interpretable scale, such as odds ratio or relative risk per SD difference	03-04	odds ratio (OR) = 1.79, 95% confidence interval (CI) = 1.59 to 2.02, PIVW-FE = 1.01×10^{-21}
		c)	If relevant, consider translating estimates of relative risk into absolute risk for a meaningful time period		Not applicable
		d)	Consider plots to visualize results (e.g. forest plot, scatterplot of associations between genetic variants and outcome versus between genetic variants and exposure)	03-04	The scatter plot and forest plot of associations between ADs-associated SNPs and sarcoidosis are presented in Figure 3A and Supplementary Figure 1A.
12	**Assessment of assumptions**				
		a)	Report the assessment of the validity of the assumptions	03-04	The results of the IVW method showed that there is no causal effect of genetic predictors of sarcoidosis on the risk of ADs (OR = 1.05, 95% CI 0.99 to 1.12, $P_{\text{IVW-MRE}} = 9.88 \times 10^{-2}$), and these results were validated by MR-Egger, weighted median, simple mode, and weighted mode (all $P > 6.25 \times 10^{-3}$, Figure 2B and Supplementary Table 6).
		b)	Report any additional statistics (e.g., assessments of heterogeneity across genetic variants, such as I^2, Q statistic or E-value)	03	For the bidirectional MR analysis of the relationships between sarcoidosis and ADs, the number of SNPs used as genetic instruments ranged from 3 (sarcoidosis) to 108 (autoimmune hypothyroidism), explaining 4.99×10^{-4} to 2.69×10^{-2} of the phenotypic variances. F-statistics for all diseases are ≥ 30, suggesting the good strength of genetic instruments (Supplementary Tables 4-5).
13	**Sensitivity analyses and additional analyses**				
		a)	Report any sensitivity analyses to assess the robustness of the main results to violations of the assumptions	03-04	We first assessed the causal effect of ADs on sarcoidosis, and the results of the IVW-FE method showed that genetic predictors of ADs were

				significantly associated with a higher risk of sarcoidosis (odds ratio (OR) = 1.79, 95% confidence interval (CI) = 1.59 to 2.02, PIVW-FE = 1.01×10^{-21}). Additionally, the MR-Egger, and Weighted median methods yielded similar results (all $P < 6.25 \times 10^{-3}$, Figure 2A and Supplementary Table 6).
		b) Report results from other sensitivity analyses or additional analyses	03-04	Then, we performed sensitivity analyses to assess our results. The results of the MR-Egger regression and MR-PRESSO global test indicated that there was no overall horizontal pleiotropy in all IVs (all P > 0.05, Table 1). However, there was evidence of heterogeneity among the SNPs of IBD (PMR-Egger = 0.049, PIVW = 0.062) and anterior iridocyclitis (PMR-Egger = 0.043, PIVW = 0.023), as shown in Table 1.
		c) Report any assessment of direction of causal relationship (e.g., bidirectional MR)	03-04	Finally, we found no evidence of reverse causality across the analyses in the MR Steiger test (all P < 0.001, Supplementary Table 7).
		d) When relevant, report and compare with estimates from non-MR analyses		Not applicable
		e) Consider additional plots to visualize results (e.g., leave-one-out analyses)	03-04	The results of the leave-one-out analysis and funnel plots are shown in Supplementary Figures 2-3.
	**DISCUSSION**			
14	**Key results**	Summarize key results with reference to study objectives	04	The results demonstrated that genetic predictors of ADs were associated with an elevated risk of developing sarcoidosis. However, we did not find evidence supporting the notion that genetic predictors of sarcoidosis are linked to an increased risk of ADs.
15	**Limitations**	Discuss limitations of the study, taking into account the validity of the IV assumptions, other sources of potential bias, and imprecision. Discuss both direction and magnitude of any potential bias and any efforts to address them	07	There are several limitations in this study that should be acknowledged.
16	**Interpretation**			
		a) Meaning: Give a cautious overall interpretation of results in the context of their limitations and in comparison with other studies	04	Previous studies have reported a significant association between ADs and sarcoidosis, with OR higher than 5 for specific ADs such as chronic active hepatitis, systemic lupus erythematosus, and sjögren syndrome. This close relationship suggests

					a potential shared immunopathogenic mechanism between sarcoidosis and ADs. Kaiser et al. proposed the classification of sarcoidosis as an autoimmune spectrum disorder, although the supporting evidence remains predominantly indirect, and the cause-effect relationship between sarcoidosis and ADs is still not fully understood. In 6 our study, we identified 87 common variants associated with ADs (including 44 different types of autoimmune-related diseases) through GWAS, and further confirmed the significant role of ADs in the development of sarcoidosis from a genetic perspective.
		b)	Mechanism: Discuss underlying biological mechanisms that could drive a potential causal relationship between the investigated exposure and the outcome, and whether the gene-environment equivalence assumption is reasonable. Use causal language carefully, clarifying that IV estimates may provide causal effects only under certain assumptions	05-07	The etiology of sarcoidosis is considered multifactorial. Previous studies have proposed that sarcoidosis pathogenesis involves a dysregulated immune system influenced by both environmental and genetic factors, although the precise mechanisms remain incompletely understood. T lymphocytes, especially CD4+ T cells, have been implicated in the development of sarcoidosis and other diseases such as gastrointestinal diseases (e.g., celiac disease, IBD), endocrine diseases (e.g., T1DM), liver diseases (e.g., primary biliary cholangitis), neurological diseases (e.g., multiple sclerosis), and cutaneous diseases (e.g., psoriasis). This may explain the frequent co-occurrence of ADs with sarcoidosis. In fact, subgroup analyses in our study also revealed that genetic predictors of T1DM, celiac disease, and IBD were causally linked to an elevated risk of sarcoidosis.
		c)	Clinical relevance: Discuss whether the results have clinical or public policy relevance, and to what extent they inform effect sizes of possible interventions	07	These findings provide potential insights into the underlying autoimmune mechanisms of sarcoidosis
17	**Generalizability**		Discuss the generalizability of the study results (a) to other populations, (b) across other exposure periods/timings, and (c) across other levels of exposure	07	First, the data used in the study were derived from the FinnGen study, which includes residents in Finland. This may limit the generalizability of the findings to other patient populations (e.g., North Americans, Australians, or Asians), as genetic and environmental factors can vary across different populations.
	**OTHER INFORMATION**				

18	**Funding**	Describe sources of funding and the role of funders in the present study and, if applicable, sources of funding for the databases and original study or studies on which the present study is based	08	Funding
19	**Data and data sharing**	Provide the data used to perform all analyses or report where and how the data can be accessed, and reference these sources in the article. Provide the statistical code needed to reproduce the results in the article, or report whether the code is publicly accessible and if so, where	07	Data availability statement
20	**Conflicts of Interest**	All authors should declare all potential conflicts of interest	08	Conflict of interest

This checklist is copyrighted by the Equator Network under the Creative Commons Attribution 3.0 Unported (CC BY 3.0) license.

1. Skrivankova VW, Richmond RC, Woolf BAR, Yarmolinsky J, Davies NM, Swanson SA, et al. Strengthening the Reporting of Observational Studies in Epidemiology using Mendelian Randomization (STROBE-MR) Statement. JAMA. 2021;under review.

2. Skrivankova VW, Richmond RC, Woolf BAR, Davies NM, Swanson SA, VanderWeele TJ, et al. Strengthening the Reporting of Observational Studies in Epidemiology using Mendelian Randomisation (STROBE-MR): Explanation and Elaboration. BMJ. 2021;375:n2233.
