# Supplementary material for: The causal relationship between sarcoidosis and autoimmune diseases: a bidirectional Mendelian randomization study in FinnGen
Source: Front Immunol. 2024 Apr 22;15:1325127. doi: 10.3389/fimmu.2024.1325127 (PMC11070530; doi:10.3389/fimmu.2024.1325127)
Supplement: Supplementary file 8 [file Table_1.docx]

**Supplementary Table 1 The forty-four different types of auto-immune related diseases in autoimmune diseases**

| **Variables** | **Phenocode** | **Sample size (cases)** | **Proportion (%)** |
| --- | --- | --- | --- |
| Rheumatoid arthritis | M13_RHEUMA | 12,555 | 12.97 |
| Relapsing polychondritis | M13_RELAPSPOLYCHONDR | 50 | 0.05 |
| Systemic lupus erythematosus | SLE_FG | 1,023 | 1.06 |
| Sjögren's syndrome | M13_SJOGREN | 2,495 | 2.58 |
| Systemic sclerosis | M13_SYSTSLCE | 619 | 0.64 |
| Dermatopolymyositis | M13_DERMATOPOLY | 421 | 0.44 |
| Wegener granulomatosis | M13_WEGENER | 413 | 0.43 |
| Microscopic polyangiitis | M13_MICROPOLYANG | 143 | 0.15 |
| Churg-Strauss syndrome | M13_CHURGSTRAUSS | 99 | 0.10 |
| Allergic purpura | D3_ALLERGPURPURA | 856 | 0.89 |
| Rheumatic fever | I9_RHEUFEV | 976 | 1.02 |
| Hypothyroidism, strict autoimmune | E4_HYTHY_AI_STRICT | 40,926 | 42.58 |
| Multiple sclerosis | G6_MS | 2,182 | 2.31 |
| Type 1 diabetes mellitus | E4_DM1 | 10,428 | 10.80 |
| Acute disseminated encephalomyelitis | G6_ADEM | 53 | 0.05 |
| Other acute disseminated demyelination | G6_DISSOTH | 277 | 0.30 |
| Narcolepsy with cataplexy | G6_NARCOCATA | 176 | 0.18 |
| Autoimmune hyperthyroidism | AUTOIMMUNE_HYPERTHYROIDISM | 1,828 | 1.90 |
| Autoimmune thyroiditis | E4_THYROIDITAUTOIM | 489 | 0.50 |
| Autoimmune polyglandular failure | E4_AUTOPOLYFAI | 36 | 0.04 |
| Drug-induced autoimmune haemolytic anaemia | D3_AIHA_DRUG | 38 | 0.04 |
| Other autoimmune haemolytic anaemias | D3_AIHA_OTHER | 280 | 0.29 |
| Myasthenia gravis | G6_MYASTHENIA | 426 | 0.44 |

| **Variables** | **Phenocode** | **Sample size (cases)** | **Proportion (%)** |
| --- | --- | --- | --- |
| Other demyelinating diseases of the central nervous system | G6_OTHDEMYEL | 1,029 | 1.07 |
| Disorders of myoneural junction and muscle in diseases classified elsewhere | G6_MYOMUSCINOTH | 200 | 0.21 |
| Coeliac disease | K11_COELIAC | 3,690 | 3.80 |
| Primary biliary cholangitis | CHIRBIL_PRIM | 557 | 0.58 |
| Guillain-Barre syndrome | G6_GUILBAR | 415 | 0.43 |
| Inflammatory bowel disease | K11_IBD | 11,416 | 11.85 |
| Psoriasis | L12_PSORIASIS | 9,267 | 9.66 |
| Vitiligo | L12_VITILIGO | 260 | 0.28 |
| Alopecia areata | L12_ALOPECAREATA | 682 | 0.72 |
| Idiopathic thrombocytopenic purpura | D3_ITP | 810 | 0.84 |
| Henoch-Schönlein purpura nephritis | N14_HENOCHSCHONLEIN_NEPHRITIS | 94 | 0.10 |
| Adrenocortical insufficiency | E4_ADDISON | 923 | 0.96 |
| Hypersensitivity angiitis | M13_HYPERANG | 135 | 0.14 |
| IgA nephropathy | N14_IGA_NEPHROPATHY | 592 | 0.62 |
| Vitamin B12 deficiency anaemia | D3_ANAEMIA_B12_DEF | 3,351 | 3.49 |
| Anterior Iridocyclitis | H7_IRIDOCYC_ANTER | 6,536 | 6.81 |
| Graves ophthalmopathy | E4_GRAVES_OPHT_STRICT | 561 | 0.59 |
| Behcet's syndrome | M13_BEHCET | 85 | 0.09 |
| Pemphigoid | L12_PEMPHIGOID | 647 | 0.66 |
| Dermatitis herpetiformis | L12_DERMATHERP | 483 | 0.50 |
| Mixed connective tissue disease | M13_MCTD | 1,849 | 1.92 |

**Supplementary Table 2 GWAS summary statistics: source and description**

| **Variables** | **Phenocode** | **Cases (N)** | **Controls (N)** | **Overlap (%)** | **Definitions** |
| --- | --- | --- | --- | --- | --- |
| Sarcoidosis | D3_SARCOIDOSIS | 4,041 | 371,255 | — | An idiopathic systemic inflammatory granulomatous disorder characterized by the presence of epithelioid and multinucleated giant cells, with minimal necrosis. It primarily affects the lungs, leading to fibrosis, and can also involve the lymph nodes, skin, liver, spleen, eyes, phalangeal bones, and parotid glands. |
| Autoimmune diseases | AUTOIMMUNE | 96,150 | 281,127 | 1.62 | A broad category of medical conditions in which the immune system mistakenly attacks and damages healthy cells, tissues, or organs within the body. |
| Rheumatoid arthritis | M13_RHEUMA | 12,555 | 240,862 | 1.26 | A chronic, systemic autoimmune disorder characterized by inflammation in the synovial membranes and articular surfaces. It primarily manifests as symmetric, erosive polyarthritis, sparing the axial skeleton. |
| Autoimmune hypothyroidism | E4_HYTHY_AI_STRICT | 40,926 | 274,069 | 1.42 | Autoimmune hypothyroidism, also known as Hashimoto's thyroiditis or Hashimoto's disease, is a specific type of hypothyroidism caused by an autoimmune reaction against the thyroid gland. |
| Type 1 diabetes mellitus | T1D | 4,196 | 308,252 | 0.51 | An endocrine disorder in which pancreatic β cells stop producing insulin, typically due to autoimmune destruction. |

| **Variables** | **Phenocode** | **Cases (N)** | **Controls (N)** | **Overlap (%)** | **Definitions** |
| --- | --- | --- | --- | --- | --- |
| Coeliac disease | K11_COELIAC | 3,690 | 361,055 | 1.12 | An autoimmune genetic disorder with an unknown pattern of inheritance that primarily affects the digestive tract. It is caused by an intolerance to dietary gluten. The consumption of gluten protein triggers an immune response, damaging the small intestinal villi and preventing the adequate absorption of nutrients. |
| Inflammatory bowel disease | K11_IBD_STRICT | 7,625 | 369,652 | 0.87 | A spectrum of inflammatory diseases affecting the small and large bowel, with an unknown cause. It includes Crohn's disease, ulcerative colitis, and colitis of indeterminate type. |
| Psoriasis | L12_PSORIASIS | 9,267 | 364,071 | 1.01 | An autoimmune condition characterized by red, well-delineated plaques with silvery scales. These plaques typically appear on the extensor surfaces and scalp. |
| Anterior iridocyclitis | H7_IRIDOCYC_ANTER | 6,536 | 370,741 | 2.71 | Anterior iridocyclitis, also known as iritis or acute anterior uveitis, is an inflammation of the iris (the colored part of the eye) and the ciliary body. It is often caused by an autoimmune reaction, where the body's immune system mistakenly targets and attacks the tissues of the eye. |

The dataset of autoimmune diseases analyzed in this study comprised a total of 44 different types of autoimmune-related diseases. Abbreviations: GWAS: Genome-wide association studies.

**Supplementary Table 3** **Potential confounders of exposure SNPs in the PhenoScanner database**

| **Exposure** | **Outcomes** | **Potential confounders** | **Excluded SNPs** | **Trait** |
| --- | --- | --- | --- | --- |
| Autoimmune diseases | Sarcoidosis | Multiple occupations and environmental exposures include exposure to moldy environments, smoking, microbial antigens, agricultural employment, metalworking, firefighting, inorganic dust, and silica dust, age, gender, and family history [1]. | None | — |
| Rheumatoid arthritis |  |  | rs11513729 | Smoking behavior |
|  |  |  | rs2395153 | Sarcoidosis |
| Autoimmune hypothyroidism |  |  | None | — |
| Type 1 diabetes mellitus |  |  | rs3184504 | Smoking behavior |
| Coeliac disease |  |  | rs3184504 | Smoking behavior |
| Inflammatory bowel disease |  |  | None | — |
| Psoriasis |  |  | rs7310615 | Smoking behavior |
| Anterior iridocyclitis |  |  | None | — |
| Sarcoidosis | Rheumatoid arthritis | Smoking, BMI, diet, alcohol, parity, breastfeeding, diet, air pollution, occupational exposures (silica, asbestos and textile-dust), age, gender, and family history [2]. | rs1431403 | Rheumatoid arthritis |
|  |  |  | rs1611637 | Rheumatoid arthritis |
|  |  |  | rs3184504 | Rheumatoid arthritis  Smoking behavior  BMI |
|  |  |  | rs9271406 | Rheumatoid arthritis |
|  |  |  | rs34536443 | Rheumatoid arthritis |
|  | Autoimmune hypothyroidism | Disturbed iodine metabolism, irradiation, thyroidectomy, alcohol, infiltrative diseases, lymphoma, infection, medications, industrial and environmental agents (smoking, inorganic dust, and silica dust), age, gender, and family history [3]. | rs1431403 | Hypothyroidism |
|  |  |  | rs3184504 | Hypothyroidism  Smoking behavior |
|  |  |  | rs34536443 | Hypothyroidism |

| **Exposure** | **Outcomes** | **Potential confounders** | **Excluded SNPs** | **Trait** |
| --- | --- | --- | --- | --- |
| Sarcoidosis | Type 1 diabetes mellitus | Parents with type1 diabetes mellitus, glutamic acid decarboxylase autoantibodies, islet cell tyrosine phosphatase 2 antibodies, zinc transporter 8 antibodies, age, and gender [4]. | rs3184504 | Type1 diabetes mellitus |
|  |  |  | rs9271406 | Diabetes |
|  | Coeliac disease | Gluten proteins, infections, age, gender, and family history [5]. | rs11757273 | Coeliac disease |
|  |  |  | rs1431403 | Coeliac disease |
|  |  |  | rs1611637 | Coeliac disease |
|  |  |  | rs3184504 | Coeliac disease |
|  |  |  | rs4455663 | Coeliac disease |
|  |  |  | rs9271406 | Coeliac disease |
|  | Inflammatory bowel disease | Smoking, microbiota, depression, age, gender, and family history [6]. | rs2024825 | Crohns disease |
|  |  |  | rs3184504 | Smoking behavior  Inflammatory bowel disease |
|  |  |  | rs9271406 | Inflammatory bowel disease |
|  | Psoriasis | Skin trauma, smoking, certain medications (such as lithium and interferon), infections (such as streptococcal), and possibly stress, age, gender, and family history [7]. | rs3184504 | Smoking behavior |
|  |  |  | rs34536443 | Psoriasis |
|  | Anterior iridocyclitis | Rheumatoid arthritis, infections, trauma, multiple sclerosis, age, gender, and family history [8,9]. | rs1431403 | Rheumatoid arthritis |
|  |  |  | rs3184504 | Rheumatoid arthritis |
|  |  |  | rs34536443 | Rheumatoid arthritis |
|  |  |  | rs9271406 | Rheumatoid arthritis |
|  |  |  | rs1611637 | Rheumatoid arthritis |

| **Exposure** | **Outcomes** | **Potential confounders** | **Excluded SNPs** | **Trait** |
| --- | --- | --- | --- | --- |
| Sarcoidosis | Autoimmune diseases | Different types of autoimmune disease and the potential confounders of rheumatoid arthritis, hypothyroidism, type1 diabetes mellitus, coeliac disease, inflammatory bowel disease, psoriasis, and anterior iridocyclitis, age, gender, and family history. | rs11757273 | Coeliac disease |
|  |  |  | rs1431403 | Rheumatoid arthritis  Coeliac disease  Hypothyroidism |
|  |  |  | rs1611637 | Coeliac disease  Rheumatoid arthritis |
|  |  |  | rs2024825 | Crohns disease |
|  |  |  | rs3184504 | Hypothyroidism  Type1 diabetes mellitus  Coeliac disease  Generalized vitiligo  Smoking behavior  Inflammatory bowel disease  Rheumatoid arthritis |
|  |  |  | rs34536443 | Psoriasis  Rheumatoid arthritis  Hypothyroidism |
|  |  |  | rs4455663 | Coeliac disease  Hypothyroidism  Ankylosing spondylitis |
|  |  |  | rs9271406 | Rheumatoid arthritis  Coeliac disease  Hyperthyroidism  Multiple sclerosis |

| **Exposure** | **Outcomes** | **Potential confounders** | **Excluded SNPs** | **Trait** |
| --- | --- | --- | --- | --- |
|  |  |  |  | Inflammatory bowel disease  Asthma  Diabetes |

The dataset of autoimmune diseases analyzed in this study comprised a total of 44 different types of autoimmune-related diseases. Abbreviations: SNPs: Single nucleotide polymorphisms.

**References**

1. Drent M, Crouser ED, Grunewald J. Challenges of Sarcoidosis and Its Management. N Engl J Med 2021; 385(11):1018-1032

2. Finckh A, Gilbert B, Hodkinson B, Bae SC, Thomas R, Deane KD, Alpizar-Rodriguez D, Lauper K. Global epidemiology of rheumatoid arthritis. Nat Rev Rheumatol 2022; 18(10):591-602

3. Chaker L, Razvi S, Bensenor IM, Azizi F, Pearce EN, Peeters RP. Hypothyroidism. Nat Rev Dis Primers 2022; 8(1):30

4. Syed FZ. Type 1 Diabetes Mellitus. Ann Intern Med 2022; 175(3):ITC33-ITC48

5. Catassi C, Verdu EF, Bai JC, Lionetti E. Coeliac disease. Lancet 2022; 399(10344):2413-2426

6. Lamb CA, Kennedy NA, Raine T, Hendy PA, Smith PJ, Limdi JK, Hayee B, Lomer MCE, Parkes GC, Selinger C, Barrett KJ, Davies RJ, Bennett C, Gittens S, Dunlop MG, Faiz O, Fraser A, Garrick V, Johnston PD, Parkes M, Sanderson J, Terry H, group IBDgec, Gaya DR, Iqbal TH, Taylor SA, Smith M, Brookes M, Hansen R, Hawthorne AB. British Society of Gastroenterology consensus guidelines on the management of inflammatory bowel disease in adults. Gut 2019; 68(Suppl 3):s1-s106

7. Walter K. Psoriasis. JAMA 2022; 327(19):1936

8. Lenoch F, Kralik V, Bartos J. Rheumatic iritis and iridocyclitis. Ann Rheum Dis 1959; 18(1):45-48

9. Lim JI, Tessler HH, Goodwin JA. Anterior granulomatous uveitis in patients with multiple sclerosis. Ophthalmology 1991; 98(2):142-145

**Supplementary Table 4** **Summary information on exposure SNPs used as genetic instruments for the MR study**

| **SNPs** | **Effect allele** | **Baseline allele** | **EAF** | **Beta** | **SE** | **Sample size** | ***P*-value** | **R^2^** | ***F*-statistics** |
| --- | --- | --- | --- | --- | --- | --- | --- | --- | --- |
| **87 SNPs for autoimmune diseases on sarcoidosis** | | | | | | | | | |
| rs10116520 | G | A | 0.402 | 0.052 | 0.006 | 377,277 | 3.32 × 10^-21^ | 1.30 × 10^-3^ | 89 |
| rs10259879 | G | A | 0.232 | 0.042 | 0.006 | 377,277 | 9.73 × 10^-11^ | 6.16 × 10^-4^ | 42 |
| rs1050976 | T | C | 0.474 | 0.030 | 0.005 | 377,277 | 1.84 × 10^-8^ | 4.57 × 10^-4^ | 32 |
| rs10518402 | C | T | 0.129 | -0.055 | 0.008 | 377,277 | 9.20 × 10^-12^ | 6.93 × 10^-4^ | 46 |
| rs10759944 | G | A | 0.653 | 0.083 | 0.006 | 377,277 | 4.69 × 10^-48^ | 3.11 × 10^-3^ | 212 |
| rs10955908 | A | C | 0.480 | 0.034 | 0.005 | 377,277 | 2.07 × 10^-10^ | 5.89 × 10^-4^ | 40 |
| rs111352680 | A | G | 0.323 | -0.032 | 0.006 | 377,277 | 3.37 × 10^-8^ | 4.51 × 10^-4^ | 30 |
| rs11203201 | G | C | 0.187 | 0.050 | 0.007 | 377,277 | 7.31 × 10^-13^ | 7.46 × 10^-4^ | 51 |
| rs113305586 | G | A | 0.025 | 0.104 | 0.017 | 377,277 | 2.37 × 10^-9^ | 5.33 × 10^-4^ | 36 |
| rs11571297 | C | T | 0.372 | -0.083 | 0.006 | 377,277 | 2.92 × 10^-50^ | 3.26 × 10^-3^ | 222 |
| rs116285139 | G | A | 0.014 | -0.158 | 0.023 | 377,277 | 1.44 × 10^-11^ | 6.98 × 10^-4^ | 46 |
| rs11680476 | C | T | 0.152 | 0.043 | 0.008 | 377,277 | 1.71 × 10^-8^ | 4.67 × 10^-4^ | 32 |
| rs116909374 | T | C | 0.031 | -0.092 | 0.016 | 377,277 | 7.96 × 10^-9^ | 5.07 × 10^-4^ | 33 |
| rs117853452 | A | G | 0.026 | 0.094 | 0.017 | 377,277 | 1.86 × 10^-8^ | 4.55 × 10^-4^ | 32 |
| rs12045440 | G | T | 0.315 | 0.035 | 0.006 | 377,277 | 2.01 × 10^-9^ | 5.23 × 10^-4^ | 36 |
| rs12055488 | G | A | 0.371 | -0.045 | 0.006 | 377,277 | 5.39 × 10^-16^ | 9.60 × 10^-4^ | 66 |
| rs12582330 | T | G | 0.676 | -0.034 | 0.006 | 377,277 | 7.03 × 10^-9^ | 4.93 × 10^-4^ | 34 |
| rs12697352 | A | G | 0.395 | -0.044 | 0.006 | 377,277 | 3.76 × 10^-15^ | 9.09 × 10^-4^ | 62 |
| rs12714240 | A | G | 0.609 | 0.031 | 0.006 | 377,277 | 3.08 × 10^-8^ | 4.48 × 10^-4^ | 31 |
| rs12967678 | A | G | 0.119 | 0.081 | 0.008 | 377,277 | 1.18 × 10^-22^ | 1.38 × 10^-3^ | 96 |
| rs13069721 | A | C | 0.247 | -0.036 | 0.006 | 377,277 | 9.04 × 10^-9^ | 4.84 × 10^-4^ | 33 |
| rs13248311 | A | C | 0.409 | -0.031 | 0.006 | 377,277 | 1.66 × 10^-8^ | 4.68 × 10^-4^ | 32 |

| **SNPs** | **Effect allele** | **Baseline allele** | **EAF** | **Beta** | **SE** | **Sample size** | ***P*-value** | **R^2^** | ***F*-statistics** |
| --- | --- | --- | --- | --- | --- | --- | --- | --- | --- |
| rs144651842 | A | G | 0.078 | 0.110 | 0.010 | 377,277 | 7.67 × 10^-28^ | 1.73 × 10^-3^ | 120 |
| rs1534424 | C | A | 0.397 | -0.035 | 0.006 | 377,277 | 2.29 × 10^-10^ | 5.88 × 10^-4^ | 40 |
| rs1552071 | A | G | 0.419 | 0.035 | 0.005 | 377,277 | 2.17 × 10^-10^ | 5.91 × 10^-4^ | 40 |
| rs1573100 | C | T | 0.232 | 0.041 | 0.006 | 377,277 | 1.74 × 10^-10^ | 5.94 × 10^-4^ | 41 |
| rs1736507 | A | C | 0.072 | -0.065 | 0.010 | 377,277 | 7.37 × 10^-10^ | 5.60 × 10^-4^ | 38 |
| rs1975371 | T | G | 0.450 | 0.038 | 0.005 | 377,277 | 3.61 × 10^-12^ | 7.05 × 10^-4^ | 48 |
| rs2068955 | G | A | 0.587 | 0.030 | 0.006 | 377,277 | 4.56 × 10^-8^ | 4.41 × 10^-4^ | 30 |
| rs2074190 | G | A | 0.292 | 0.039 | 0.006 | 377,277 | 4.45 × 10^-11^ | 6.30 × 10^-4^ | 43 |
| rs2208397 | G | T | 0.697 | 0.039 | 0.006 | 377,277 | 3.80 × 10^-11^ | 6.42 × 10^-4^ | 44 |
| rs2260976 | G | A | 0.653 | 0.043 | 0.006 | 377,277 | 2.31 × 10^-14^ | 8.53 × 10^-4^ | 58 |
| rs229526 | C | G | 0.179 | 0.046 | 0.007 | 377,277 | 4.99 × 10^-11^ | 6.24 × 10^-4^ | 43 |
| rs2396084 | G | A | 0.676 | 0.038 | 0.006 | 377,277 | 4.58 × 10^-11^ | 6.41 × 10^-4^ | 43 |
| rs244687 | G | A | 0.748 | -0.039 | 0.006 | 377,277 | 5.02 × 10^-10^ | 5.60 × 10^-4^ | 39 |
| rs2531989 | A | G | 0.818 | -0.041 | 0.007 | 377,277 | 4.96 × 10^-9^ | 4.96 × 10^-4^ | 34 |
| rs281379 | A | G | 0.409 | 0.036 | 0.005 | 377,277 | 6.13 × 10^-11^ | 6.22 × 10^-4^ | 43 |
| rs28420485 | C | T | 0.618 | -0.030 | 0.006 | 377,277 | 4.97 × 10^-8^ | 4.32 × 10^-4^ | 30 |
| rs310752 | A | G | 0.472 | -0.037 | 0.005 | 377,277 | 1.01 × 10^-11^ | 6.79 × 10^-4^ | 46 |
| rs33980500 | T | C | 0.072 | 0.059 | 0.010 | 377,277 | 1.22 × 10^-8^ | 4.63 × 10^-4^ | 32 |
| rs34236350 | T | C | 0.267 | 0.037 | 0.006 | 377,277 | 1.10 × 10^-9^ | 5.43 × 10^-4^ | 37 |
| rs34517439 | A | C | 0.128 | 0.044 | 0.008 | 377,277 | 4.97 × 10^-8^ | 4.31 × 10^-4^ | 30 |
| rs3778753 | G | A | 0.441 | 0.051 | 0.005 | 377,277 | 7.97 × 10^-21^ | 1.28 × 10^-3^ | 88 |
| rs3826556 | G | C | 0.058 | 0.066 | 0.011 | 377,277 | 6.06 × 10^-9^ | 4.85 × 10^-4^ | 34 |
| rs3861929 | T | C | 0.692 | 0.043 | 0.006 | 377,277 | 2.01 × 10^-13^ | 7.93 × 10^-4^ | 54 |
| rs4274624 | T | C | 0.769 | -0.081 | 0.006 | 377,277 | 4.56 × 10^-37^ | 2.33 × 10^-3^ | 162 |

| **SNPs** | **Effect allele** | **Baseline allele** | **EAF** | **Beta** | **SE** | **Sample size** | ***P*-value** | **R^2^** | ***F*-statistics** |
| --- | --- | --- | --- | --- | --- | --- | --- | --- | --- |
| rs4585948 | C | T | 0.453 | -0.031 | 0.005 | 377,277 | 9.47 × 10^-4^ | 4.81 × 10^-4^ | 33 |
| rs4755449 | T | C | 0.349 | -0.032 | 0.006 | 377,277 | 2.38 × 10^-8^ | 4.58 × 10^-4^ | 31 |
| rs4796664 | T | C | 0.644 | -0.040 | 0.006 | 377,277 | 2.38 × 10^-12^ | 7.15 × 10^-4^ | 49 |
| rs4808766 | C | G | 0.250 | -0.035 | 0.006 | 377,277 | 2.39 × 10^-8^ | 4.57 × 10^-4^ | 31 |
| rs568999 | C | G | 0.315 | -0.041 | 0.006 | 377,277 | 1.31 × 10^-12^ | 7.36 × 10^-4^ | 50 |
| rs5757599 | C | A | 0.301 | 0.045 | 0.006 | 377,277 | 1.24 × 10^-14^ | 8.59 × 10^-4^ | 59 |
| rs60050490 | T | C | 0.045 | 0.078 | 0.013 | 377,277 | 1.70 × 10^-9^ | 5.16 × 10^-4^ | 36 |
| rs60600003 | G | T | 0.102 | 0.060 | 0.009 | 377,277 | 1.72 × 10^-11^ | 6.51 × 10^-4^ | 45 |
| rs61759532 | T | C | 0.190 | 0.054 | 0.007 | 377,277 | 3.10 × 10^-15^ | 9.02 × 10^-4^ | 62 |
| rs62626308 | A | C | 0.119 | -0.049 | 0.008 | 377,277 | 6.47 × 10^-9^ | 5.00 × 10^-4^ | 34 |
| rs6448432 | A | G | 0.283 | 0.048 | 0.006 | 377,277 | 5.26 × 10^-16^ | 9.51 × 10^-4^ | 66 |
| rs6545846 | G | A | 0.526 | -0.040 | 0.005 | 377,277 | 9.28 × 10^-14^ | 8.14 × 10^-4^ | 56 |
| rs66654254 | A | G | 0.435 | -0.038 | 0.006 | 377,277 | 4.99 × 10^-12^ | 7.10 × 10^-4^ | 48 |
| rs67514601 | G | A | 0.339 | 0.035 | 0.006 | 377,277 | 1.01 × 10^-9^ | 5.45 × 10^-4^ | 37 |
| rs6762648 | A | T | 0.244 | -0.040 | 0.006 | 377,277 | 1.62 × 10^-10^ | 6.02 × 10^-4^ | 41 |
| rs6831973 | C | T | 0.556 | 0.035 | 0.005 | 377,277 | 2.14 × 10^-10^ | 5.89 × 10^-4^ | 40 |
| rs6902545 | A | G | 0.356 | 0.044 | 0.006 | 377,277 | 9.57 × 10^-15^ | 8.74 × 10^-4^ | 60 |
| rs6908626 | T | G | 0.115 | 0.086 | 0.008 | 377,277 | 5.20 × 10^-25^ | 1.52 × 10^-3^ | 107 |
| rs7001653 | A | G | 0.443 | -0.036 | 0.005 | 377,277 | 1.94 × 10^-11^ | 6.57 × 10^-4^ | 45 |
| rs7043516 | C | A | 0.166 | -0.040 | 0.007 | 377,277 | 4.09 × 10^-8^ | 4.47 × 10^-4^ | 30 |
| rs7173565 | C | T | 0.289 | 0.042 | 0.006 | 377,277 | 1.15 × 10^-12^ | 7.33 × 10^-4^ | 51 |
| rs72695823 | T | C | 0.064 | 0.072 | 0.011 | 377,277 | 5.65 × 10^-11^ | 6.21 × 10^-4^ | 43 |
| rs7516736 | G | A | 0.625 | -0.046 | 0.006 | 377,277 | 2.99 × 10^-16^ | 9.75 × 10^-4^ | 67 |
| rs75663797 | T | C | 0.035 | 0.112 | 0.015 | 377,277 | 1.54 × 10^-14^ | 8.38 × 10^-4^ | 59 |

| **SNPs** | **Effect allele** | **Baseline allele** | **EAF** | **Beta** | **SE** | **Sample size** | ***P*-value** | **R^2^** | ***F*-statistics** |
| --- | --- | --- | --- | --- | --- | --- | --- | --- | --- |
| rs7567564 | T | A | 0.293 | -0.033 | 0.006 | 377,277 | 2.41 × 10^-8^ | 4.57 × 10^-4^ | 31 |
| rs75998928 | G | A | 0.056 | -0.093 | 0.012 | 377,277 | 6.05 × 10^-15^ | 9.19 × 10^-4^ | 61 |
| rs7675267 | G | C | 0.124 | -0.053 | 0.008 | 377,277 | 3.12 × 10^-10^ | 6.04 × 10^-4^ | 40 |
| rs77241188 | C | T | 0.046 | 0.107 | 0.013 | 377,277 | 2.08 × 10^-17^ | 1.02 × 10^-4^ | 72 |
| rs773107 | G | A | 0.280 | 0.054 | 0.006 | 377,277 | 6.05 × 10^-19^ | 1.16 × 10^-4^ | 79 |
| rs7731626 | A | G | 0.279 | -0.034 | 0.006 | 377,277 | 1.38 × 10^-8^ | 4.72 × 10^-4^ | 32 |
| rs77634652 | C | G | 0.233 | 0.066 | 0.006 | 377,277 | 4.40 × 10^-25^ | 1.53 × 10^-4^ | 107 |
| rs7825794 | T | C | 0.137 | -0.056 | 0.008 | 377,277 | 1.54 × 10^-12^ | 7.43 × 10^-4^ | 50 |
| rs78456138 | T | C | 0.020 | -0.149 | 0.020 | 377,277 | 2.56 × 10^-14^ | 8.86 × 10^-4^ | 58 |
| rs78458460 | T | G | 0.222 | 0.050 | 0.006 | 377,277 | 1.51 × 10^-14^ | 8.55 × 10^-4^ | 59 |
| rs7902146 | T | C | 0.750 | -0.049 | 0.006 | 377,277 | 2.93 × 10^-15^ | 9.03 × 10^-4^ | 62 |
| rs7936434 | C | G | 0.414 | 0.030 | 0.005 | 377,277 | 4.87 × 10^-8^ | 4.33 × 10^-4^ | 30 |
| rs8045630 | C | T | 0.579 | -0.038 | 0.005 | 377,277 | 3.07 × 10^-12^ | 7.14 × 10^-4^ | 49 |
| rs8193 | T | C | 0.353 | 0.034 | 0.006 | 377,277 | 1.25 × 10^-9^ | 5.39 × 10^-4^ | 37 |
| rs9390488 | C | T | 0.460 | 0.031 | 0.005 | 377,277 | 1.37 × 10^-8^ | 4.71 × 10^-4^ | 32 |
| rs947474 | A | G | 0.819 | 0.044 | 0.007 | 377,277 | 5.76 × 10^-10^ | 5.64 × 10^-4^ | 38 |
| rs9687206 | G | A | 0.467 | 0.048 | 0.005 | 377,277 | 5.25 × 10^-19^ | 1.16 × 10^-3^ | 79 |
| **11 SNPs for rheumatoid arthritis on sarcoidosis** | | | | | | | | | |
| rs10821948 | A | C | 0.426 | 0.076 | 0.013 | 253,417 | 1.34 × 10^-8^ | 2.83 × 10^-3^ | 32 |
| rs11758148 | C | A | 0.125 | 0.181 | 0.019 | 253,417 | 3.72 × 10^-21^ | 7.12 × 10^-3^ | 89 |
| rs2735075 | G | T | 0.424 | -0.085 | 0.013 | 253,417 | 2.34 × 10^-10^ | 3.50 × 10^-3^ | 40 |
| rs3757387 | C | T | 0.425 | 0.091 | 0.013 | 253,417 | 6.23 × 10^-12^ | 4.08 × 10^-3^ | 47 |
| rs4274624 | T | C | 0.769 | -0.111 | 0.015 | 253,417 | 4.84 × 10^-13^ | 4.41 × 10^-3^ | 52 |
| rs548877 | G | A | 0.176 | 0.096 | 0.017 | 253,417 | 1.46 × 10^-8^ | 2.70 × 10^-3^ | 32 |

| **SNPs** | **Effect allele** | **Baseline allele** | **EAF** | **Beta** | **SE** | **Sample size** | ***P*-value** | **R^2^** | ***F*-statistics** |
| --- | --- | --- | --- | --- | --- | --- | --- | --- | --- |
| rs6065926 | G | A | 0.729 | 0.085 | 0.015 | 253,417 | 1.56 × 10^-8^ | 2.84 × 10^-3^ | 32 |
| rs6456160 | C | T | 0.557 | -0.079 | 0.013 | 253,417 | 2.34 × 10^-9^ | 3.10 × 10^-3^ | 36 |
| rs78248443 | T | C | 0.075 | -0.179 | 0.026 | 253,417 | 9.69 × 10^-12^ | 4.44 × 10^-3^ | 46 |
| rs78782944 | T | C | 0.095 | 0.125 | 0.022 | 253,417 | 1.13 × 10^-8^ | 2.71 × 10^-3^ | 33 |
| rs8002731 | C | A | 0.363 | -0.090 | 0.014 | 253,417 | 7.18 × 10^-11^ | 3.77 × 10^-3^ | 42 |
| **108 SNPs for autoimmune hypothyroidism on sarcoidosis** | | | | | | | | | |
| rs10116520 | G | A | 0.401 | 0.092 | 0.008 | 314,995 | 4.99 × 10^-31^ | 4.09 × 10^-3^ | 134 |
| rs10118880 | A | G | 0.724 | -0.060 | 0.009 | 314,995 | 5.89 × 10^-12^ | 1.44 × 10^-3^ | 47 |
| rs10166287 | G | T | 0.103 | -0.092 | 0.013 | 314,995 | 2.39 × 10^-12^ | 1.56 × 10^-3^ | 49 |
| rs10259879 | G | A | 0.232 | 0.064 | 0.009 | 314,995 | 6.65 × 10^-12^ | 1.45 × 10^-3^ | 47 |
| rs10416358 | G | A | 0.286 | 0.079 | 0.009 | 314,995 | 2.31 × 10^-20^ | 2.57 × 10^-3^ | 86 |
| rs10494077 | A | G | 0.214 | 0.109 | 0.009 | 314,995 | 2.92 × 10^-31^ | 4.03 × 10^-3^ | 135 |
| rs10514934 | C | T | 0.151 | -0.082 | 0.011 | 314,995 | 1.20 × 10^-13^ | 1.74 × 10^-3^ | 55 |
| rs10817674 | A | C | 0.404 | 0.049 | 0.008 | 314,995 | 6.71 × 10^-10^ | 1.17 × 10^-3^ | 38 |
| rs10818050 | G | A | 0.655 | 0.246 | 0.008 | 314,995 | 9.33 × 10^-190^ | 2.73 × 10^-2^ | 863 |
| rs10858026 | G | T | 0.807 | 0.070 | 0.010 | 314,995 | 3.78 × 10^-12^ | 1.52 × 10^-3^ | 48 |
| rs11079035 | A | G | 0.203 | 0.076 | 0.010 | 314,995 | 4.20 × 10^-15^ | 1.85 × 10^-3^ | 62 |
| rs11086103 | A | G | 0.582 | 0.050 | 0.008 | 314,995 | 2.47 × 10^-10^ | 1.24 × 10^-3^ | 40 |
| rs111352680 | A | G | 0.324 | -0.055 | 0.008 | 314,995 | 7.13 × 10^-11^ | 1.33 × 10^-3^ | 42 |
| rs11571297 | C | T | 0.373 | -0.125 | 0.008 | 314,995 | 6.10 × 10^-53^ | 7.26 × 10^-3^ | 235 |
| rs116776245 | A | G | 0.083 | 0.087 | 0.014 | 314,995 | 3.50 × 10^-10^ | 1.16 × 10^-3^ | 39 |
| rs116909374 | T | C | 0.031 | -0.213 | 0.024 | 314,995 | 7.53 × 10^-19^ | 2.69 × 10^-3^ | 79 |
| rs11897732 | A | G | 0.582 | 0.046 | 0.008 | 314,995 | 6.29 × 10^-9^ | 1.04 × 10^-3^ | 34 |
| rs11935941 | C | A | 0.123 | -0.141 | 0.012 | 314,995 | 3.59 × 10^-30^ | 4.32 × 10^-3^ | 130 |

| **SNPs** | **Effect allele** | **Baseline allele** | **EAF** | **Beta** | **SE** | **Sample size** | ***P*-value** | **R^2^** | ***F*-statistics** |
| --- | --- | --- | --- | --- | --- | --- | --- | --- | --- |
| rs11969311 | C | A | 0.252 | -0.057 | 0.009 | 314,995 | 2.97 × 10^-10^ | 1.22 × 10^-3^ | 40 |
| rs1203940 | C | T | 0.792 | 0.103 | 0.010 | 314,995 | 1.13 × 10^-25^ | 3.47 × 10^-3^ | 110 |
| rs12206423 | T | C | 0.149 | 0.061 | 0.011 | 314,995 | 1.61 × 10^-8^ | 9.55 × 10^-4^ | 32 |
| rs1239704 | A | G | 0.692 | -0.051 | 0.008 | 314,995 | 1.31 × 10^-9^ | 1.12 × 10^-3^ | 37 |
| rs12407089 | C | G | 0.316 | 0.092 | 0.008 | 314,995 | 2.32 × 10^-28^ | 3.69 × 10^-3^ | 122 |
| rs12540388 | T | A | 0.186 | -0.066 | 0.010 | 314,995 | 1.05 × 10^-10^ | 1.31 × 10^-3^ | 42 |
| rs12697352 | A | G | 0.396 | -0.050 | 0.008 | 314,995 | 5.24 × 10^-10^ | 1.19 × 10^-3^ | 39 |
| rs12756019 | A | G | 0.606 | -0.058 | 0.008 | 314,995 | 6.53 × 10^-13^ | 1.59 × 10^-3^ | 52 |
| rs12897126 | A | T | 0.887 | -0.102 | 0.012 | 314,995 | 5.37 × 10^-17^ | 2.10 × 10^-3^ | 70 |
| rs12923006 | A | G | 0.180 | 0.072 | 0.010 | 314,995 | 9.23 × 10^-13^ | 1.53 × 10^-3^ | 51 |
| rs12967678 | A | G | 0.118 | 0.074 | 0.012 | 314,995 | 5.88 × 10^-10^ | 1.15 × 10^-3^ | 38 |
| rs13137589 | G | A | 0.368 | -0.063 | 0.008 | 314,995 | 1.17 × 10^-14^ | 1.86 × 10^-3^ | 60 |
| rs1317983 | C | T | 0.681 | 0.102 | 0.009 | 314,995 | 2.69 × 10^-33^ | 4.54 × 10^-3^ | 145 |
| rs1319091 | A | C | 0.795 | -0.056 | 0.010 | 314,995 | 7.93 × 10^-9^ | 1.02 × 10^-3^ | 33 |
| rs13447704 | C | T | 0.027 | -0.141 | 0.025 | 314,995 | 1.28 × 10^-8^ | 1.04 × 10^-3^ | 32 |
| rs141686764 | G | A | 0.019 | -0.164 | 0.030 | 314,995 | 3.33 × 10^-8^ | 9.94 × 10^-4^ | 31 |
| rs151234 | C | G | 0.123 | 0.085 | 0.012 | 314,995 | 4.82 × 10^-13^ | 1.57 × 10^-3^ | 52 |
| rs1534424 | C | A | 0.398 | -0.056 | 0.008 | 314,995 | 1.99 × 10^-12^ | 1.52 × 10^-3^ | 49 |
| rs17364832 | G | T | 0.291 | 0.079 | 0.009 | 314,995 | 2.85 × 10^-20^ | 2.56 × 10^-3^ | 85 |
| rs17389938 | C | G | 0.144 | 0.067 | 0.011 | 314,995 | 1.61 × 10^-9^ | 1.09 × 10^-3^ | 36 |
| rs17447487 | T | C | 0.052 | -0.103 | 0.018 | 314,995 | 1.44 × 10^-8^ | 1.05 × 10^-3^ | 32 |
| rs1808192 | G | A | 0.592 | -0.046 | 0.008 | 314,995 | 8.01 × 10^-9^ | 1.02 × 10^-3^ | 33 |
| rs1885013 | A | G | 0.694 | 0.050 | 0.009 | 314,995 | 3.22 × 10^-9^ | 1.08 × 10^-3^ | 35 |
| rs1990760 | T | C | 0.586 | 0.064 | 0.008 | 314,995 | 1.07 × 10^-15^ | 1.98 × 10^-3^ | 64 |

| **SNPs** | **Effect allele** | **Baseline allele** | **EAF** | **Beta** | **SE** | **Sample size** | ***P*-value** | **R^2^** | ***F*-statistics** |
| --- | --- | --- | --- | --- | --- | --- | --- | --- | --- |
| rs200293726 | T | A | 0.305 | -0.084 | 0.009 | 314,995 | 1.89 × 10^-22^ | 2.99 × 10^-3^ | 95 |
| rs2046045 | G | T | 0.414 | 0.127 | 0.008 | 314,995 | 1.81 × 10^-58^ | 7.87 × 10^-3^ | 260 |
| rs2049218 | T | C | 0.386 | -0.103 | 0.008 | 314,995 | 4.19 × 10^-37^ | 5.01 × 10^-3^ | 162 |
| rs210192 | G | A | 0.215 | 0.073 | 0.009 | 314,995 | 1.54 × 10^-14^ | 1.79 × 10^-3^ | 59 |
| rs2124594 | C | T | 0.318 | -0.059 | 0.008 | 314,995 | 2.23 × 10^-12^ | 1.53 × 10^-3^ | 49 |
| rs2235544 | A | C | 0.545 | -0.055 | 0.008 | 314,995 | 1.87 × 10^-12^ | 1.52 × 10^-3^ | 50 |
| rs229528 | T | C | 0.396 | 0.072 | 0.008 | 314,995 | 1.29 × 10^-19^ | 2.49 × 10^-3^ | 82 |
| rs2345568 | G | A | 0.373 | -0.065 | 0.008 | 314,995 | 1.01 × 10^-15^ | 1.98 × 10^-3^ | 64 |
| rs244687 | G | A | 0.748 | -0.074 | 0.009 | 314,995 | 1.08 × 10^-16^ | 2.07 × 10^-3^ | 69 |
| rs2476601 | G | A | 0.855 | -0.309 | 0.011 | 314,995 | 8.69 × 10^-188^ | 2.37 × 10^-2^ | 854 |
| rs2629649 | G | T | 0.313 | 0.051 | 0.008 | 314,995 | 1.47 × 10^-9^ | 1.11 × 10^-3^ | 37 |
| rs2739043 | G | A | 0.633 | -0.055 | 0.008 | 314,995 | 9.56 × 10^-12^ | 1.41 × 10^-3^ | 46 |
| rs28391281 | C | T | 0.546 | -0.050 | 0.008 | 314,995 | 1.31 × 10^-10^ | 1.26 × 10^-3^ | 41 |
| rs28396553 | T | C | 0.650 | -0.046 | 0.008 | 314,995 | 2.05 × 10^-8^ | 9.67 × 10^-4^ | 31 |
| rs2972343 | G | A | 0.106 | -0.088 | 0.013 | 314,995 | 9.64 × 10^-12^ | 1.47 × 10^-3^ | 46 |
| rs2976908 | G | T | 0.484 | -0.061 | 0.008 | 314,995 | 1.72 × 10^-14^ | 1.83 × 10^-3^ | 59 |
| rs30233 | A | G | 0.587 | -0.054 | 0.008 | 314,995 | 1.07 × 10^-11^ | 1.42 × 10^-3^ | 46 |
| rs3103991 | G | A | 0.748 | 0.056 | 0.009 | 314,995 | 7.61 × 10^-10^ | 1.17 × 10^-3^ | 38 |
| rs35717611 | T | C | 0.378 | 0.054 | 0.008 | 314,995 | 2.89 × 10^-11^ | 1.36 × 10^-3^ | 44 |
| rs3778752 | T | G | 0.440 | 0.045 | 0.008 | 314,995 | 9.08 × 10^-9^ | 1.01 × 10^-3^ | 33 |
| rs3946137 | G | A | 0.349 | 0.046 | 0.008 | 314,995 | 1.85 × 10^-8^ | 9.64 × 10^-4^ | 32 |
| rs41177 | A | G | 0.405 | -0.049 | 0.008 | 314,995 | 1.18 × 10^-9^ | 1.14 × 10^-3^ | 37 |
| rs4606850 | T | C | 0.165 | 0.065 | 0.010 | 314,995 | 5.14 × 10^-10^ | 1.17 × 10^-3^ | 39 |
| rs4704447 | G | A | 0.569 | -0.046 | 0.008 | 314,995 | 6.46 × 10^-9^ | 1.04 × 10^-3^ | 34 |

| **SNPs** | **Effect allele** | **Baseline allele** | **EAF** | **Beta** | **SE** | **Sample size** | ***P*-value** | **R^2^** | ***F*-statistics** |
| --- | --- | --- | --- | --- | --- | --- | --- | --- | --- |
| rs4853459 | C | T | 0.770 | -0.123 | 0.009 | 314,995 | 2.03 × 10^-41^ | 5.41 × 10^-3^ | 182 |
| rs4912068 | G | C | 0.089 | -0.081 | 0.014 | 314,995 | 9.51 × 10^-9^ | 1.06 × 10^-3^ | 33 |
| rs56011703 | T | C | 0.081 | 0.084 | 0.014 | 314,995 | 2.95 × 10^-9^ | 1.06 × 10^-3^ | 35 |
| rs56159866 | T | C | 0.232 | -0.059 | 0.009 | 314,995 | 2.58 × 10^-10^ | 1.25 × 10^-3^ | 40 |
| rs568999 | C | G | 0.316 | -0.053 | 0.008 | 314,995 | 2.59 × 10^-10^ | 1.23 × 10^-3^ | 40 |
| rs56983610 | A | C | 0.131 | -0.104 | 0.012 | 314,995 | 1.34 × 10^-18^ | 2.47 × 10^-3^ | 77 |
| rs57652885 | T | C | 0.053 | -0.107 | 0.018 | 314,995 | 2.16 × 10^-9^ | 1.15 × 10^-3^ | 36 |
| rs61201527 | C | A | 0.124 | 0.064 | 0.012 | 314,995 | 5.00 × 10^-8^ | 8.96 × 10^-3^ | 30 |
| rs61759532 | T | C | 0.189 | 0.078 | 0.010 | 314,995 | 4.37 × 10^-15^ | 1.86 × 10^-3^ | 62 |
| rs61916675 | G | A | 0.317 | 0.054 | 0.008 | 314,995 | 9.43 × 10^-11^ | 1.28 × 10^-3^ | 42 |
| rs61938962 | T | C | 0.319 | 0.081 | 0.008 | 314,995 | 4.09 × 10^-22^ | 2.82 × 10^-3^ | 93 |
| rs6448432 | A | G | 0.282 | 0.066 | 0.009 | 314,995 | 2.11 × 10^-14^ | 1.77 × 10^-3^ | 58 |
| rs6471875 | A | C | 0.405 | 0.049 | 0.008 | 314,995 | 7.93 × 10^-10^ | 1.16 × 10^-3^ | 38 |
| rs66760320 | T | C | 0.202 | -0.064 | 0.010 | 314,995 | 7.64 × 10^-11^ | 1.31 × 10^-3^ | 42 |
| rs6831973 | C | T | 0.556 | 0.056 | 0.008 | 314,995 | 1.36 × 10^-12^ | 1.54 × 10^-3^ | 50 |
| rs7043516 | C | A | 0.166 | -0.107 | 0.011 | 314,995 | 2.66 × 10^-23^ | 3.15 × 10^-3^ | 99 |
| rs706779 | C | T | 0.378 | -0.067 | 0.008 | 314,995 | 8.36 × 10^-17^ | 2.14 × 10^-3^ | 69 |
| rs7127620 | G | A | 0.359 | -0.051 | 0.008 | 314,995 | 3.62 × 10^-10^ | 1.22 × 10^-3^ | 39 |
| rs713427 | C | T | 0.222 | 0.057 | 0.009 | 314,995 | 8.88 × 10^-10^ | 1.14 × 10^-3^ | 38 |
| rs71430783 | T | G | 0.180 | 0.093 | 0.010 | 314,995 | 2.43 × 10^-20^ | 2.55 × 10^-3^ | 85 |
| rs71641308 | T | C | 0.096 | 0.075 | 0.013 | 314,995 | 1.46 × 10^-8^ | 9.69 × 10^-4^ | 32 |
| rs72729322 | T | C | 0.210 | 0.057 | 0.010 | 314,995 | 1.85 × 10^-9^ | 1.10 × 10^-3^ | 36 |
| rs72751538 | T | G | 0.066 | 0.095 | 0.016 | 314,995 | 1.11 × 10^-9^ | 1.11 × 10^-3^ | 37 |
| rs735000 | T | C | 0.120 | 0.077 | 0.012 | 314,995 | 1.25 × 10^-10^ | 1.24 × 10^-3^ | 41 |

| **SNPs** | **Effect allele** | **Baseline allele** | **EAF** | **Beta** | **SE** | **Sample size** | ***P*-value** | **R^2^** | ***F*-statistics** |
| --- | --- | --- | --- | --- | --- | --- | --- | --- | --- |
| rs7444908 | G | A | 0.357 | -0.064 | 0.008 | 314,995 | 6.79 × 10^-15^ | 1.87 × 10^-3^ | 61 |
| rs753760 | G | C | 0.262 | -0.082 | 0.009 | 314,995 | 3.74 × 10^-20^ | 2.62 × 10^-3^ | 85 |
| rs76032549 | T | C | 0.170 | 0.064 | 0.010 | 314,995 | 6.07 × 10^-10^ | 1.16 × 10^-3^ | 38 |
| rs76169968 | A | G | 0.099 | -0.086 | 0.013 | 314,995 | 1.14 × 10^-10^ | 1.32 × 10^-3^ | 42 |
| rs7701443 | G | A | 0.381 | -0.047 | 0.008 | 314,995 | 8.30 × 10^-9^ | 1.04 × 10^-3^ | 33 |
| rs774121 | C | T | 0.749 | -0.071 | 0.009 | 314,995 | 2.16 × 10^-15^ | 1.90 × 10^-3^ | 63 |
| rs78953577 | T | G | 0.248 | -0.061 | 0.009 | 314,995 | 1.67 × 10^-11^ | 1.41 × 10^-3^ | 45 |
| rs7902146 | T | C | 0.750 | -0.073 | 0.009 | 314,995 | 3.71 × 10^-16^ | 2.01 × 10^-3^ | 66 |
| rs79051898 | T | C | 0.121 | -0.068 | 0.012 | 314,995 | 2.58 × 10^-8^ | 9.72 × 10^-4^ | 31 |
| rs794999 | G | A | 0.772 | -0.068 | 0.009 | 314,995 | 2.13 × 10^-13^ | 1.63 × 10^-3^ | 54 |
| rs8006310 | G | A | 0.504 | -0.054 | 0.008 | 314,995 | 4.12 × 10^-12^ | 1.47 × 10^-3^ | 48 |
| rs8193 | T | C | 0.353 | 0.058 | 0.008 | 314,995 | 1.10 × 10^-12^ | 1.55 × 10^-3^ | 51 |
| rs914960 | A | T | 0.150 | -0.063 | 0.011 | 314,995 | 1.28 × 10^-8^ | 1.01 × 10^-3^ | 32 |
| rs9292 | G | A | 0.968 | -0.124 | 0.022 | 314,995 | 1.43 × 10^-8^ | 9.45 × 10^-4^ | 32 |
| rs9378805 | C | A | 0.469 | 0.045 | 0.008 | 314,995 | 6.91 × 10^-9^ | 1.03 × 10^-3^ | 34 |
| rs938726 | C | A | 0.731 | -0.068 | 0.009 | 314,995 | 1.24 × 10^-14^ | 1.80 × 10^-3^ | 59 |
| rs9497965 | T | C | 0.337 | 0.066 | 0.008 | 314,995 | 2.29 × 10^-15^ | 1.92 × 10^-3^ | 63 |
| rs9981704 | T | C | 0.179 | 0.056 | 0.010 | 314,995 | 3.13 × 10^-8^ | 9.28 × 10^-4^ | 31 |
| **20 SNPs for type 1 diabetes mellitus on sarcoidosis** | | | | | | | | | |
| rs114313544 | C | A | 0.050 | 0.396 | 0.045 | 312,448 | 1.58 × 10^-18^ | 1.50 × 10^-2^ | 77 |
| rs114355928 | T | G | 0.033 | 0.576 | 0.052 | 312,448 | 4.06 × 10^-28^ | 2.11 × 10^-2^ | 121 |
| rs11571297 | C | T | 0.372 | -0.172 | 0.023 | 312,448 | 2.22 × 10^-13^ | 1.38 × 10^-2^ | 54 |
| rs116965083 | T | C | 0.042 | -0.344 | 0.063 | 312,448 | 3.92 × 10^-8^ | 9.45 × 10^-3^ | 30 |
| rs11964319 | T | C | 0.326 | 0.173 | 0.023 | 312,448 | 1.21 × 10^-13^ | 1.31 × 10^-2^ | 55 |

| **SNPs** | **Effect allele** | **Baseline allele** | **EAF** | **Beta** | **SE** | **Sample size** | ***P*-value** | **R^2^** | ***F*-statistics** |
| --- | --- | --- | --- | --- | --- | --- | --- | --- | --- |
| rs140149 | T | C | 0.348 | 0.148 | 0.023 | 312,448 | 1.67 × 10^-10^ | 9.96 × 10^-3^ | 41 |
| rs151234 | C | G | 0.124 | 0.184 | 0.033 | 312,448 | 1.60 × 10^-8^ | 7.34 × 10^-3^ | 32 |
| rs1639106 | T | C | 0.036 | 0.506 | 0.051 | 312,448 | 7.41 × 10^-23^ | 1.76 × 10^-2^ | 97 |
| rs17179816 | T | C | 0.042 | -0.767 | 0.068 | 312,448 | 9.41 × 10^-30^ | 4.78 × 10^-2^ | 128 |
| rs17200067 | T | C | 0.034 | -0.682 | 0.075 | 312,448 | 1.16 × 10^-19^ | 3.05 × 10^-2^ | 82 |
| rs1985706 | G | A | 0.151 | 0.170 | 0.030 | 312,448 | 2.25 × 10^-8^ | 7.40 × 10^-3^ | 31 |
| rs2476601 | G | A | 0.853 | -0.521 | 0.028 | 312,448 | 1.19 × 10^-78^ | 6.83 × 10^-2^ | 353 |
| rs28895017 | A | C | 0.011 | -1.407 | 0.159 | 312,448 | 9.88 × 10^-19^ | 4.21 × 10^-2^ | 78 |
| rs56994090 | C | T | 0.495 | -0.155 | 0.023 | 312,448 | 5.21 × 10^-12^ | 1.21 × 10^-2^ | 48 |
| rs601338 | A | G | 0.375 | 0.148 | 0.023 | 312,448 | 9.42 × 10^-11^ | 1.03 × 10^-2^ | 42 |
| rs60888743 | G | A | 0.231 | -0.172 | 0.027 | 312,448 | 3.41 × 10^-10^ | 1.05 × 10^-2^ | 39 |
| rs7130222 | G | T | 0.262 | -0.155 | 0.026 | 312,448 | 3.98 × 10^-9^ | 9.23 × 10^-3^ | 35 |
| rs74203920 | T | C | 0.037 | 0.352 | 0.054 | 312,448 | 1.03 × 10^-10^ | 8.76 × 10^-3^ | 42 |
| rs876497 | A | G | 0.280 | 0.173 | 0.024 | 312,448 | 1.81 × 10^-12^ | 1.20 × 10^-2^ | 50 |
| rs9934969 | A | G | 0.304 | -0.148 | 0.025 | 312,448 | 2.68 × 10^-9^ | 9.29 × 10^-3^ | 35 |
| **9 SNPs for coeliac disease on sarcoidosis** | | | | | | | | | |
| rs2230365 | T | C | 0.170 | -0.492 | 0.034 | 364,745 | 2.59 × 10^-47^ | 6.81 × 10^-2^ | 209 |
| rs67572105 | A | G | 0.071 | -0.280 | 0.050 | 364,745 | 1.66 × 10^-8^ | 1.03 × 10^-2^ | 32 |
| rs68006638 | A | G | 0.047 | 1.167 | 0.038 | 364,745 | 1.00 × 10^-200^ | 1.22 × 10^-1^ | 944 |
| rs6822844 | T | G | 0.114 | -0.227 | 0.039 | 364,745 | 6.54 × 10^-9^ | 1.04 × 10^-2^ | 34 |
| rs6917350 | A | G | 0.142 | 0.423 | 0.030 | 364,745 | 1.42 × 10^-44^ | 4.38 × 10^-2^ | 196 |
| rs6933404 | C | T | 0.189 | 0.190 | 0.029 | 364,745 | 2.65 × 10^-11^ | 1.11 × 10^-2^ | 44 |
| rs71327040 | T | G | 0.107 | 0.249 | 0.036 | 364,745 | 3.05 × 10^-12^ | 1.19 × 10^-2^ | 49 |
| rs7758498 | A | G | 0.136 | 0.203 | 0.033 | 364,745 | 1.05 × 10^-9^ | 9.67 × 10^-3^ | 37 |

| **SNPs** | **Effect allele** | **Baseline allele** | **EAF** | **Beta** | **SE** | **Sample size** | ***P*-value** | **R^2^** | ***F*-statistics** |
| --- | --- | --- | --- | --- | --- | --- | --- | --- | --- |
| rs9488932 | T | C | 0.261 | -0.150 | 0.027 | 364,745 | 3.55 × 10^-8^ | 8.67 × 10^-3^ | 30 |
| **35 SNPs for inflammatory bowel disease on sarcoidosis** | | | | | | | | | |
| rs10737481 | G | T | 0.510 | 0.145 | 0.016 | 377,277 | 1.04 × 10^-18^ | 1.05 × 10^-2^ | 78 |
| rs10761659 | G | A | 0.528 | 0.129 | 0.016 | 377,277 | 3.47 × 10^-15^ | 8.33 × 10^-3^ | 62 |
| rs10931828 | T | C | 0.441 | -0.095 | 0.017 | 377,277 | 9.43 × 10^-9^ | 4.47 × 10^-3^ | 33 |
| rs11590283 | A | G | 0.737 | -0.102 | 0.018 | 377,277 | 2.31 × 10^-8^ | 4.06 × 10^-3^ | 31 |
| rs117115824 | T | A | 0.102 | -0.167 | 0.030 | 377,277 | 2.12 × 10^-8^ | 5.11 × 10^-3^ | 31 |
| rs11771806 | T | C | 0.117 | 0.135 | 0.025 | 377,277 | 4.51 × 10^-8^ | 3.79 × 10^-3^ | 30 |
| rs12132298 | C | T | 0.213 | -0.163 | 0.021 | 377,277 | 3.18 × 10^-15^ | 8.89 × 10^-3^ | 62 |
| rs12536069 | C | T | 0.068 | 0.252 | 0.030 | 377,277 | 9.74 × 10^-17^ | 8.10 × 10^-3^ | 69 |
| rs12755372 | C | A | 0.250 | -0.117 | 0.019 | 377,277 | 1.27 × 10^-9^ | 5.17 × 10^-3^ | 37 |
| rs13165038 | C | T | 0.312 | -0.131 | 0.018 | 377,277 | 3.69 × 10^-13^ | 7.39 × 10^-3^ | 53 |
| rs142695953 | A | C | 0.186 | 0.180 | 0.020 | 377,277 | 1.04 × 10^-18^ | 9.85 × 10^-3^ | 78 |
| rs191615076 | A | C | 0.040 | 0.246 | 0.039 | 377,277 | 3.97 × 10^-10^ | 4.70 × 10^-3^ | 39 |
| rs1990760 | T | C | 0.586 | -0.100 | 0.017 | 377,277 | 1.42 × 10^-9^ | 4.88 × 10^-3^ | 37 |
| rs2735075 | G | T | 0.424 | -0.134 | 0.017 | 377,277 | 8.84 × 10^-16^ | 8.78 × 10^-3^ | 65 |
| rs2823272 | A | T | 0.257 | -0.110 | 0.019 | 377,277 | 9.44 × 10^-9^ | 4.65 × 10^-3^ | 33 |
| rs2836883 | A | G | 0.247 | -0.141 | 0.019 | 377,277 | 4.34 × 10^-13^ | 7.40 × 10^-3^ | 52 |
| rs3197999 | A | G | 0.392 | 0.170 | 0.017 | 377,277 | 1.65 × 10^-24^ | 1.37 × 10^-2^ | 104 |
| rs3736162 | C | G | 0.281 | -0.108 | 0.018 | 377,277 | 4.34 × 10^-9^ | 4.75 × 10^-3^ | 34 |
| rs376365394 | T | C | 0.040 | 0.246 | 0.039 | 377,277 | 3.11 × 10^-10^ | 4.68 × 10^-3^ | 40 |
| rs3827023 | T | C | 0.188 | -0.142 | 0.022 | 377,277 | 4.22 × 10^-11^ | 6.19 × 10^-3^ | 44 |
| rs4372078 | G | T | 0.739 | 0.121 | 0.019 | 377,277 | 1.74 × 10^-10^ | 5.67 × 10^-3^ | 41 |
| rs4676410 | A | G | 0.271 | 0.166 | 0.018 | 377,277 | 5.15 × 10^-20^ | 1.08 × 10^-2^ | 84 |

| **SNPs** | **Effect allele** | **Baseline allele** | **EAF** | **Beta** | **SE** | **Sample size** | ***P*-value** | **R^2^** | ***F*-statistics** |
| --- | --- | --- | --- | --- | --- | --- | --- | --- | --- |
| rs4730275 | T | G | 0.334 | -0.114 | 0.018 | 377,277 | 1.10 × 10^-10^ | 5.75 × 10^-3^ | 42 |
| rs4731531 | A | G | 0.443 | 0.098 | 0.016 | 377,277 | 2.67 × 10^-9^ | 4.75 × 10^-3^ | 35 |
| rs4807543 | T | G | 0.032 | 0.239 | 0.043 | 377,277 | 3.64 × 10^-8^ | 3.54 × 10^-3^ | 30 |
| rs56893428 | T | C | 0.451 | 0.091 | 0.016 | 377,277 | 2.97 × 10^-8^ | 4.13 × 10^-3^ | 31 |
| rs6017342 | C | A | 0.559 | 0.123 | 0.017 | 377,277 | 1.48 × 10^-13^ | 7.43 × 10^-3^ | 55 |
| rs6556416 | C | A | 0.743 | 0.113 | 0.019 | 377,277 | 3.89 × 10^-9^ | 4.87 × 10^-3^ | 35 |
| rs6762648 | A | T | 0.244 | -0.140 | 0.020 | 377,277 | 7.19 × 10^-13^ | 7.27 × 10^-3^ | 51 |
| rs7551957 | C | T | 0.446 | -0.141 | 0.017 | 377,277 | 1.58 × 10^-17^ | 9.88 × 10^-3^ | 73 |
| rs7936434 | C | G | 0.414 | 0.134 | 0.016 | 377,277 | 5.45 × 10^-16^ | 8.66 × 10^-3^ | 66 |
| rs895123 | G | C | 0.194 | 0.142 | 0.020 | 377,277 | 1.47 × 10^-12^ | 6.34 × 10^-3^ | 50 |
| rs9272353 | C | G | 0.193 | -0.246 | 0.022 | 377,277 | 6.85 × 10^-30^ | 1.89 × 10^-2^ | 129 |
| rs9607629 | G | A | 0.125 | -0.178 | 0.026 | 377,277 | 7.43 × 10^-12^ | 6.95 × 10^-3^ | 47 |
| rs9617090 | T | C | 0.372 | -0.129 | 0.017 | 377,277 | 5.30 × 10^-14^ | 7.77 × 10^-3^ | 57 |
| **20 SNPs for psoriasis on sarcoidosis** | | | | | | | | | |
| rs12206050 | T | A | 0.32 | 0.101 | 0.016 | 373,338 | 1.50 × 10^-10^ | 4.47 × 10^-3^ | 41 |
| rs1250566 | A | G | 0.408 | -0.089 | 0.015 | 373,338 | 6.64 × 10^-9^ | 3.82 × 10^-3^ | 34 |
| rs13153019 | C | T | 0.273 | 0.102 | 0.016 | 373,338 | 6.67 × 10^-10^ | 4.12 × 10^-3^ | 38 |
| rs139298380 | A | G | 0.028 | 0.313 | 0.042 | 373,338 | 4.59 × 10^-14^ | 5.28 × 10^-3^ | 57 |
| rs1611704 | T | C | 0.345 | 0.225 | 0.015 | 373,338 | 3.43 × 10^-49^ | 2.29 × 10^-2^ | 217 |
| rs16903065 | A | C | 0.116 | -0.141 | 0.024 | 373,338 | 5.01 × 10^-9^ | 4.08 × 10^-3^ | 34 |
| rs2769979 | C | T | 0.605 | -0.096 | 0.015 | 373,338 | 2.64 × 10^-10^ | 4.38 × 10^-3^ | 40 |
| rs28998802 | A | G | 0.184 | 0.161 | 0.019 | 373,338 | 7.83 × 10^-18^ | 7.76 × 10^-3^ | 74 |
| rs33980500 | T | C | 0.072 | 0.238 | 0.027 | 373,338 | 8.26 × 10^-19^ | 7.59 × 10^-3^ | 78 |
| rs4400255 | A | T | 0.099 | 0.181 | 0.024 | 373,338 | 2.14 × 10^-14^ | 5.89 × 10^-3^ | 58 |

| **SNPs** | **Effect allele** | **Baseline allele** | **EAF** | **Beta** | **SE** | **Sample size** | ***P*-value** | **R^2^** | ***F*-statistics** |
| --- | --- | --- | --- | --- | --- | --- | --- | --- | --- |
| rs60600003 | G | T | 0.102 | 0.15 | 0.024 | 373,338 | 2.62 × 10^-10^ | 4.10 × 10^-3^ | 40 |
| rs653169 | G | A | 0.57 | -0.086 | 0.015 | 373,338 | 1.14 × 10^-8^ | 3.64 × 10^-3^ | 33 |
| rs674451 | C | T | 0.344 | 0.128 | 0.015 | 373,338 | 1.29 × 10^-16^ | 7.42 × 10^-3^ | 68 |
| rs74817271 | A | G | 0.074 | 0.225 | 0.027 | 373,338 | 4.12 × 10^-17^ | 6.96 × 10^-3^ | 71 |
| rs7542079 | C | T | 0.562 | 0.095 | 0.015 | 373,338 | 2.87 × 10^-10^ | 4.45 × 10^-3^ | 40 |
| rs76741620 | G | A | 0.058 | 0.174 | 0.03 | 373,338 | 1.06 × 10^-8^ | 3.32 × 10^-3^ | 33 |
| rs78456138 | T | C | 0.02 | -0.365 | 0.059 | 373,338 | 6.61 × 10^-10^ | 5.31 × 10^-3^ | 38 |
| rs847 | C | T | 0.635 | 0.127 | 0.016 | 373,338 | 8.63 × 10^-16^ | 7.43 × 10^-3^ | 65 |
| rs8904 | A | G | 0.388 | -0.126 | 0.015 | 373,338 | 2.83 × 10^-16^ | 7.56 × 10^-3^ | 67 |
| rs9346778 | T | C | 0.164 | -0.117 | 0.021 | 373,338 | 1.55 × 10^-8^ | 3.74 × 10^-3^ | 32 |
| **6 SNPs for anterior iridocyclitis on sarcoidosis** | | | | | | | | | |
| rs12194518 | G | C | 0.101 | 0.309 | 0.027 | 377,277 | 6.23 × 10^-30^ | 1.74 × 10^-2^ | 129 |
| rs1233398 | T | C | 0.190 | 0.249 | 0.021 | 377,277 | 2.00 × 10^-31^ | 1.91 × 10^-2^ | 136 |
| rs13160562 | A | G | 0.272 | -0.179 | 0.020 | 377,277 | 1.46 × 10^-18^ | 1.27 × 10^-2^ | 77 |
| rs190364070 | T | C | 0.054 | 0.910 | 0.030 | 377,277 | 1.00 × 10^-200^ | 8.42 × 10^-2^ | 949 |
| rs296563 | G | T | 0.727 | 0.111 | 0.020 | 377,277 | 3.24 × 10^-8^ | 4.93 × 10^-3^ | 31 |
| rs303888 | G | A | 0.434 | -0.153 | 0.018 | 377,277 | 3.48 × 10^-17^ | 1.14 × 10^-2^ | 71 |
| **3 SNPs for sarcoidosis on autoimmune diseases** | | | | | | | | | |
| rs1079242 | G | A | 0.561 | -0.246 | 0.022 | 375,296 | 2.75 × 10^-28^ | 2.99 × 10^-2^ | 122 |
| rs1860217 | C | G | 0.419 | 0.132 | 0.023 | 375,296 | 4.41 × 10^-9^ | 8.51 × 10^-3^ | 34 |
| rs7581601 | C | A | 0.297 | 0.134 | 0.024 | 375,296 | 2.42 × 10^-8^ | 7.50 × 10^-3^ | 31 |
| **4 SNPs for sarcoidosis on rheumatoid arthritis** | | | | | | | | | |
| rs1079242 | G | A | 0.561 | -0.246 | 0.022 | 375,296 | 2.75 × 10^-28^ | 2.99 × 10^-2^ | 122 |
| rs11757273 | A | C | 0.394 | 0.187 | 0.023 | 375,296 | 1.50 × 10^-16^ | 1.66 × 10^-2^ | 68 |

| **SNPs** | **Effect allele** | **Baseline allele** | **EAF** | **Beta** | **SE** | **Sample size** | ***P*-value** | **R^2^** | ***F*-statistics** |
| --- | --- | --- | --- | --- | --- | --- | --- | --- | --- |
| rs1860217 | C | G | 0.419 | 0.132 | 0.013 | 375,296 | 4.41 × 10^-9^ | 2.99 × 10^-2^ | 122 |
| rs2024825 | C | T | 0.836 | -0.206 | 0.018 | 375,296 | 9.94 × 10^-13^ | 1.66 × 10^-2^ | 68 |
| **4 SNPs for sarcoidosis on hypothyroidism** | | | | | | | | | |
| rs1079242 | G | A | 0.561 | -0.246 | 0.022 | 375,296 | 2.75 × 10^-28^ | 2.99 × 10^-2^ | 122 |
| rs1611637 | G | A | 0.524 | 0.135 | 0.022 | 375,296 | 1.83 × 10^-9^ | 9.08 × 10^-3^ | 36 |
| rs2024825 | C | T | 0.836 | -0.206 | 0.029 | 375,296 | 9.94 × 10^-13^ | 1.16 × 10^-2^ | 51 |
| rs7581601 | C | A | 0.297 | 0.134 | 0.024 | 375,296 | 2.42 × 10^-8^ | 7.50 × 10^-3^ | 31 |
| **4 SNPs for sarcoidosis on type 1 diabetes mellitus** | | | | | | | | | |
| rs11757273 | A | C | 0.394 | 0.187 | 0.023 | 375,296 | 1.50 × 10^-16^ | 1.66 × 10^-2^ | 68 |
| rs1860217 | C | G | 0.419 | 0.132 | 0.023 | 375,296 | 4.41 × 10^-9^ | 8.51 × 10^-2^ | 34 |
| rs34536443 | C | G | 0.030 | -0.432 | 0.075 | 375,296 | 7.75 × 10^-9^ | 1.10 × 10^-2^ | 33 |
| rs663743 | A | G | 0.375 | -0.172 | 0.023 | 375,296 | 1.91 × 10^-13^ | 1.39 × 10^-2^ | 54 |
| **5 SNPs for sarcoidosis on coeliac disease** | | | | | | | | | |
| rs1079242 | G | A | 0.561 | -0.246 | 0.022 | 375,296 | 2.75 × 10^-28^ | 2.99 × 10^-2^ | 122 |
| rs1860217 | C | G | 0.419 | 0.132 | 0.023 | 375,296 | 4.41 × 10^-9^ | 8.51 × 10^-3^ | 34 |
| rs2024825 | C | T | 0.836 | -0.206 | 0.029 | 375,296 | 9.94 × 10^-13^ | 1.16 × 10^-2^ | 51 |
| rs34536443 | C | G | 0.030 | -0.432 | 0.075 | 375,296 | 7.75 × 10^-9^ | 1.10 × 10^-2^ | 33 |
| rs7581601 | C | A | 0.297 | 0.134 | 0.024 | 375,296 | 2.42 × 10^-8^ | 7.50 × 10^-3^ | 31 |
| **7 SNPs for sarcoidosis on inflammatory bowel disease** | | | | | | | | | |
| rs11757273 | A | C | 0.394 | 0.187 | 0.023 | 375,296 | 1.50 × 10^-16^ | 1.66 × 10^-2^ | 68 |
| rs1431403 | C | T | 0.245 | 0.302 | 0.025 | 375,296 | 6.63 × 10^-35^ | 3.38 × 10^-2^ | 152 |
| rs1860217 | C | G | 0.419 | 0.132 | 0.023 | 375,296 | 4.41 × 10^-9^ | 8.51 × 10^-3^ | 34 |
| rs34536443 | C | G | 0.030 | -0.432 | 0.075 | 375,296 | 7.75 × 10^-9^ | 1.10 × 10^-2^ | 33 |
| rs4455663 | C | T | 0.391 | -0.261 | 0.023 | 375,296 | 2.34 × 10^-29^ | 3.25 × 10^-2^ | 127 |

| **SNPs** | **Effect allele** | **Baseline allele** | **EAF** | **Beta** | **SE** | **Sample size** | ***P*-value** | **R^2^** | ***F*-statistics** |
| --- | --- | --- | --- | --- | --- | --- | --- | --- | --- |
| rs663743 | A | G | 0.375 | -0.172 | 0.023 | 375,296 | 1.91 × 10^-13^ | 1.39 × 10^-2^ | 54 |
| rs7581601 | C | A | 0.297 | 0.134 | 0.024 | 375,296 | 2.42 × 10^-8^ | 7.50 × 10^-3^ | 31 |
| **5 SNPs for sarcoidosis on psoriasis** | | | | | | | | | |
| rs1079242 | G | A | 0.561 | -0.246 | 0.022 | 375,296 | 2.75 × 10^-28^ | 2.99 × 10^-2^ | 122 |
| rs11757273 | A | C | 0.394 | 0.187 | 0.023 | 375,296 | 1.50 × 10^-16^ | 1.66 × 10^-2^ | 68 |
| rs1860217 | C | G | 0.419 | 0.132 | 0.023 | 375,296 | 4.41 × 10^-9^ | 8.51 × 10^-3^ | 34 |
| rs2024825 | C | T | 0.836 | -0.206 | 0.029 | 375,296 | 9.94 × 10^-13^ | 1.16 × 10^-2^ | 51 |
| rs7581601 | C | A | 0.297 | 0.134 | 0.024 | 375,296 | 2.42 × 10^-8^ | 7.50 × 10^-3^ | 31 |
| **6 SNPs for sarcoidosis on anterior iridocyclitis** | | | | | | | | | |
| rs1079242 | G | A | 0.561 | -0.246 | 0.022 | 375,296 | 2.75 × 10^-28^ | 2.99 × 10^-2^ | 122 |
| rs11757273 | A | C | 0.394 | 0.187 | 0.023 | 375,296 | 1.50 × 10^-16^ | 1.66 × 10^-2^ | 68 |
| rs1860217 | C | G | 0.419 | 0.132 | 0.023 | 375,296 | 4.41 × 10^-9^ | 8.51 × 10^-3^ | 34 |
| rs2024825 | C | T | 0.836 | -0.206 | 0.029 | 375,296 | 9.94 × 10^-13^ | 1.16 × 10^-2^ | 51 |
| rs663743 | A | G | 0.375 | -0.172 | 0.023 | 375,296 | 1.91 × 10^-13^ | 1.39 × 10^-2^ | 54 |
| rs7581601 | C | A | 0.297 | 0.134 | 0.024 | 375,296 | 2.42 × 10^-8^ | 7.50 × 10^-3^ | 31 |

The dataset of autoimmune diseases analyzed in this study comprised a total of 44 different types of autoimmune-related diseases. A *P*-value < 5 × 10^−8^ was considered genome-wide significant. Abbreviations: MR: Mendelian randomization; SNPs: Single nucleotide polymorphisms; EAF: Effect allele frequency; SE: Standard error.

**Supplementary Table 5 Summary information on outcome SNPs used as genetic instruments for the MR study**

| **SNPs** | **Effect allele** | **Baseline allele** | **EAF** | **Beta** | **SE** | ***P*-value** |
| --- | --- | --- | --- | --- | --- | --- |
| **87 SNPs for autoimmune diseases on sarcoidosis** | | | | | | |
| rs10116520 | G | A | 0.402 | 0.014 | 0.023 | 5.50 × 10^-1^ |
| rs10259879 | G | A | 0.232 | 0.059 | 0.027 | 2.65 × 10^-2^ |
| rs1050976 | T | C | 0.474 | -0.016 | 0.022 | 4.64 × 10^-1^ |
| rs10518402 | C | T | 0.130 | -0.085 | 0.033 | 1.06 × 10^-2^ |
| rs10759944 | G | A | 0.653 | 0.019 | 0.024 | 4.29 × 10^-1^ |
| rs10955908 | A | C | 0.480 | 0.035 | 0.022 | 1.19 × 10^-1^ |
| rs111352680 | A | G | 0.323 | -0.021 | 0.024 | 3.92 × 10^-1^ |
| rs11203201 | G | C | 0.187 | 0.069 | 0.029 | 1.61 × 10^-2^ |
| rs113305586 | G | A | 0.025 | 0.065 | 0.075 | 3.86 × 10^-1^ |
| rs11571297 | C | T | 0.372 | -0.040 | 0.023 | 8.65 × 10^-2^ |
| rs116285139 | G | A | 0.014 | -0.054 | 0.094 | 5.68 × 10^-1^ |
| rs11680476 | C | T | 0.152 | -0.037 | 0.032 | 2.44 × 10^-1^ |
| rs116909374 | T | C | 0.031 | -0.071 | 0.066 | 2.85 × 10^-1^ |
| rs117853452 | A | G | 0.026 | 0.056 | 0.07 | 4.25 × 10^-1^ |
| rs12045440 | G | T | 0.315 | 0.021 | 0.024 | 3.77 × 10^-1^ |
| rs12055488 | G | A | 0.371 | -0.013 | 0.023 | 5.81 × 10^-1^ |
| rs12582330 | T | G | 0.676 | 0.006 | 0.024 | 8.01 × 10^-1^ |
| rs12697352 | A | G | 0.395 | -0.015 | 0.023 | 5.23 × 10^-1^ |
| rs12714240 | A | G | 0.609 | 0.034 | 0.023 | 1.40 × 10^-1^ |
| rs12967678 | A | G | 0.119 | 0.097 | 0.034 | 4.14 × 10^-3^ |
| rs13069721 | A | C | 0.247 | 0.021 | 0.026 | 4.10 × 10^-1^ |
| rs13248311 | A | C | 0.409 | 0.01 | 0.023 | 6.67 × 10^-1^ |
| rs144651842 | A | G | 0.078 | 0.154 | 0.041 | 1.62 × 10^-4^ |
| rs1534424 | C | A | 0.397 | -0.031 | 0.023 | 1.75 × 10^-1^ |
| rs1552071 | A | G | 0.419 | 0.011 | 0.023 | 6.34 × 10^-1^ |
| rs1573100 | C | T | 0.232 | 0.062 | 0.027 | 1.98 × 10^-2^ |
| rs1736507 | A | C | 0.072 | -0.076 | 0.043 | 7.87 × 10^-2^ |
| rs1975371 | T | G | 0.450 | 0.007 | 0.023 | 7.72 × 10^-1^ |
| rs2068955 | G | A | 0.587 | 0.031 | 0.023 | 1.81 × 10^-1^ |
| rs2074190 | G | A | 0.292 | -0.004 | 0.025 | 8.80 × 10^-1^ |
| rs2208397 | G | T | 0.697 | -0.006 | 0.025 | 8.10 × 10^-1^ |
| rs2260976 | G | A | 0.653 | 0.013 | 0.024 | 5.86 × 10^-1^ |
| rs229526 | C | G | 0.179 | 0.034 | 0.029 | 2.45 × 10^-1^ |
| rs2396084 | G | A | 0.676 | 0.031 | 0.024 | 2.00 × 10^-1^ |
| rs244687 | G | A | 0.747 | -0.036 | 0.026 | 1.61 × 10^-1^ |
| rs2531989 | A | G | 0.818 | 0.024 | 0.029 | 4.18 × 10^-1^ |
| rs281379 | A | G | 0.409 | 0.052 | 0.023 | 2.16 × 10^-2^ |
| rs28420485 | C | T | 0.618 | 0.014 | 0.023 | 5.41 × 10^-1^ |
| rs310752 | A | G | 0.472 | -0.077 | 0.023 | 5.83 × 10^-4^ |

| **SNPs** | **Effect allele** | **Baseline allele** | **EAF** | **Beta** | **SE** | ***P*-value** |
| --- | --- | --- | --- | --- | --- | --- |
| rs33980500 | T | C | 0.072 | -0.045 | 0.043 | 3.01 × 10^-1^ |
| rs34236350 | T | C | 0.267 | -0.004 | 0.025 | 8.75 × 10^-1^ |
| rs34517439 | A | C | 0.128 | 0.052 | 0.034 | 1.24 × 10^-1^ |
| rs3778753 | G | A | 0.441 | -0.004 | 0.023 | 8.60 × 10^-1^ |
| rs3826556 | G | C | 0.058 | 0.036 | 0.048 | 4.59 × 10^-1^ |
| rs3861929 | T | C | 0.692 | -0.028 | 0.024 | 2.54 × 10^-1^ |
| rs4274624 | T | C | 0.769 | -0.028 | 0.027 | 3.00 × 10^-1^ |
| rs4585948 | C | T | 0.453 | -0.032 | 0.023 | 1.51 × 10^-1^ |
| rs4755449 | T | C | 0.349 | -0.034 | 0.024 | 1.44 × 10^-1^ |
| rs4796664 | T | C | 0.644 | -0.074 | 0.023 | 1.41 × 10^-3^ |
| rs4808766 | C | G | 0.250 | -0.002 | 0.026 | 9.50 × 10^-1^ |
| rs568999 | C | G | 0.315 | -0.046 | 0.024 | 5.83 × 10^-2^ |
| rs5757599 | C | A | 0.301 | 0.026 | 0.024 | 2.91 × 10^-1^ |
| rs60050490 | T | C | 0.045 | 0.068 | 0.054 | 2.07 × 10^-1^ |
| rs60600003 | G | T | 0.102 | 0.068 | 0.037 | 6.65 × 10^-2^ |
| rs61759532 | T | C | 0.190 | 0.071 | 0.029 | 1.27 × 10^-2^ |
| rs62626308 | A | C | 0.119 | 0.024 | 0.035 | 5.00 × 10^-1^ |
| rs6448432 | A | G | 0.283 | 0.028 | 0.025 | 2.62 × 10^-1^ |
| rs6545846 | G | A | 0.526 | -0.058 | 0.023 | 1.07 × 10^-2^ |
| rs66654254 | A | G | 0.435 | -0.032 | 0.023 | 1.56 × 10^-1^ |
| rs67514601 | G | A | 0.339 | 0.011 | 0.024 | 6.32 × 10^-1^ |
| rs6762648 | A | T | 0.244 | -0.015 | 0.026 | 5.77 × 10^-1^ |
| rs6831973 | C | T | 0.556 | 0.051 | 0.023 | 2.42 × 10^-2^ |
| rs6902545 | A | G | 0.356 | 0.061 | 0.023 | 9.21 × 10^-3^ |
| rs6908626 | T | G | 0.115 | 0.103 | 0.034 | 2.61 × 10^-3^ |
| rs7001653 | A | G | 0.443 | -0.059 | 0.023 | 8.62 × 10^-3^ |
| rs7043516 | C | A | 0.166 | -0.015 | 0.03 | 6.24 × 10^-1^ |
| rs7173565 | C | T | 0.289 | 0.052 | 0.025 | 3.51 × 10^-2^ |
| rs72695823 | T | C | 0.064 | 0.005 | 0.047 | 9.08 × 10^-1^ |
| rs7516736 | G | A | 0.625 | 0.009 | 0.023 | 7.08 × 10^-1^ |
| rs75663797 | T | C | 0.035 | 0.020 | 0.062 | 7.43 × 10^-1^ |
| rs7567564 | T | A | 0.293 | -0.056 | 0.025 | 2.34 × 10^-2^ |
| rs75998928 | G | A | 0.056 | -0.065 | 0.05 | 1.92 × 10^-1^ |
| rs7675267 | G | C | 0.124 | 0.008 | 0.035 | 8.27 × 10^-1^ |
| rs77241188 | C | T | 0.046 | 0.018 | 0.053 | 7.42 × 10^-1^ |
| rs773107 | G | A | 0.280 | -0.023 | 0.025 | 3.53 × 10^-1^ |
| rs7731626 | A | G | 0.279 | -0.035 | 0.025 | 1.66 × 10^-1^ |
| rs77634652 | C | G | 0.233 | 0.075 | 0.026 | 4.05 × 10^-3^ |
| rs7825794 | T | C | 0.137 | -0.016 | 0.033 | 6.20 × 10^-1^ |
| rs78456138 | T | C | 0.020 | -0.061 | 0.08 | 4.49 × 10^-1^ |
| rs78458460 | T | G | 0.222 | 0.000 | 0.027 | 9.92 × 10^-1^ |
| rs7902146 | T | C | 0.750 | -0.021 | 0.026 | 4.20 × 10^-1^ |

| **SNPs** | **Effect allele** | **Baseline allele** | **EAF** | **Beta** | **SE** | ***P*-value** |
| --- | --- | --- | --- | --- | --- | --- |
| rs7936434 | C | G | 0.414 | -0.004 | 0.023 | 8.47 × 10^-1^ |
| rs8045630 | C | T | 0.579 | -0.010 | 0.023 | 6.65 × 10^-1^ |
| rs8193 | T | C | 0.353 | 0.017 | 0.023 | 4.60 × 10^-1^ |
| rs9390488 | C | T | 0.460 | 0.009 | 0.023 | 6.85 × 10^-1^ |
| rs947474 | A | G | 0.819 | 0.013 | 0.029 | 6.50 × 10^-1^ |
| rs9687206 | G | A | 0.467 | 0.042 | 0.023 | 5.92 × 10^-2^ |
| **11 SNPs for rheumatoid arthritis on sarcoidosis** | | | | | | |
| rs10821948 | A | C | 0.425 | -0.003 | 0.023 | 9.09 × 10^-1^ |
| rs11758148 | C | A | 0.125 | 0.006 | 0.034 | 8.63 × 10^-1^ |
| rs2735075 | G | T | 0.424 | 0.004 | 0.023 | 8.60 × 10^-1^ |
| rs3757387 | C | T | 0.425 | -0.008 | 0.023 | 7.19 × 10^-1^ |
| rs4274624 | T | C | 0.769 | -0.028 | 0.027 | 3.00 × 10^-1^ |
| rs548877 | G | A | 0.177 | -0.013 | 0.030 | 6.59 × 10^-1^ |
| rs6065926 | G | A | 0.728 | -0.027 | 0.025 | 2.90 × 10^-1^ |
| rs6456160 | C | T | 0.557 | 0.007 | 0.023 | 7.62 × 10^-1^ |
| rs78248443 | T | C | 0.075 | 0.087 | 0.043 | 4.44 × 10^-2^ |
| rs78782944 | T | C | 0.095 | -0.015 | 0.039 | 6.98 × 10^-1^ |
| rs8002731 | C | A | 0.363 | -0.020 | 0.023 | 3.99 × 10^-1^ |
| **108 SNPs for autoimmune hypothyroidism on sarcoidosis** | | | | | | |
| rs10116520 | G | A | 0.402 | 0.014 | 0.023 | 5.50 × 10^-1^ |
| rs10118880 | A | G | 0.724 | 0.002 | 0.025 | 9.37 × 10^-1^ |
| rs10166287 | G | T | 0.102 | -0.015 | 0.037 | 6.81 × 10^-1^ |
| rs10259879 | G | A | 0.232 | 0.059 | 0.027 | 2.65 × 10^-2^ |
| rs10416358 | G | A | 0.285 | 0.042 | 0.025 | 9.15 × 10^-2^ |
| rs10494077 | A | G | 0.213 | 0.008 | 0.027 | 7.82 × 10^-1^ |
| rs10514934 | C | T | 0.15 | -0.019 | 0.031 | 5.42 × 10^-1^ |
| rs10817674 | A | C | 0.404 | 0.044 | 0.023 | 5.22 × 10^-2^ |
| rs10818050 | G | A | 0.654 | 0.022 | 0.024 | 3.50 × 10^-1^ |
| rs10858026 | G | T | 0.808 | -0.052 | 0.029 | 6.64 × 10^-2^ |
| rs11079035 | A | G | 0.203 | 0.000 | 0.028 | 9.96 × 10^-1^ |
| rs11086103 | A | G | 0.582 | -0.019 | 0.023 | 3.94 × 10^-1^ |
| rs111352680 | A | G | 0.323 | -0.021 | 0.024 | 3.92 × 10^-1^ |
| rs11571297 | C | T | 0.372 | -0.040 | 0.023 | 8.65 × 10^-2^ |
| rs116776245 | A | G | 0.084 | 0.032 | 0.041 | 4.31 × 10^-1^ |
| rs116909374 | T | C | 0.031 | -0.071 | 0.066 | 2.85 × 10^-1^ |
| rs11897732 | A | G | 0.582 | -0.041 | 0.023 | 7.45 × 10^-2^ |
| rs11935941 | C | A | 0.123 | 0.008 | 0.035 | 8.21 × 10^-1^ |
| rs11969311 | C | A | 0.252 | 0.043 | 0.026 | 9.26 × 10^-2^ |
| rs1203940 | C | T | 0.791 | -0.012 | 0.028 | 6.51 × 10^-1^ |
| rs12206423 | T | C | 0.148 | -0.002 | 0.032 | 9.38 × 10^-1^ |
| rs1239704 | A | G | 0.692 | -0.029 | 0.024 | 2.42 × 10^-1^ |
| rs12407089 | C | G | 0.315 | 0.021 | 0.024 | 3.91 × 10^-1^ |

| **SNPs** | **Effect allele** | **Baseline allele** | **EAF** | **Beta** | **SE** | ***P*-value** |
| --- | --- | --- | --- | --- | --- | --- |
| rs12540388 | T | A | 0.186 | -0.041 | 0.029 | 1.56 × 10^-1^ |
| rs12697352 | A | G | 0.395 | -0.015 | 0.023 | 5.23 × 10^-1^ |
| rs12756019 | A | G | 0.604 | 0.005 | 0.023 | 8.40 × 10^-1^ |
| rs12897126 | A | T | 0.887 | 0.021 | 0.036 | 5.57 × 10^-1^ |
| rs12923006 | A | G | 0.18 | -0.023 | 0.029 | 4.37 × 10^-1^ |
| rs12967678 | A | G | 0.119 | 0.097 | 0.034 | 4.14 × 10^-3^ |
| rs13137589 | G | A | 0.368 | 0.005 | 0.023 | 8.27 × 10^-1^ |
| rs1317983 | C | T | 0.680 | 0.026 | 0.024 | 2.92 × 10^-1^ |
| rs1319091 | A | C | 0.795 | -0.035 | 0.028 | 2.20 × 10^-1^ |
| rs13447704 | C | T | 0.027 | -0.02 | 0.069 | 7.73 × 10^-1^ |
| rs141686764 | G | A | 0.019 | -0.014 | 0.084 | 8.68 × 10^-1^ |
| rs151234 | C | G | 0.124 | -0.048 | 0.034 | 1.63 × 10^-1^ |
| rs1534424 | C | A | 0.397 | -0.031 | 0.023 | 1.75 × 10^-1^ |
| rs17364832 | G | T | 0.290 | -0.001 | 0.025 | 9.65 × 10^-1^ |
| rs17389938 | C | G | 0.144 | 0.008 | 0.032 | 7.98 × 10^-1^ |
| rs17447487 | T | C | 0.052 | -0.071 | 0.051 | 1.62 × 10^-1^ |
| rs1808192 | G | A | 0.592 | 0.037 | 0.023 | 1.05 × 10^-1^ |
| rs1885013 | A | G | 0.695 | -0.006 | 0.024 | 8.15 × 10^-1^ |
| rs1990760 | T | C | 0.586 | 0.017 | 0.023 | 4.61 × 10^-1^ |
| rs200293726 | T | A | 0.307 | 0.037 | 0.024 | 1.33 × 10^-1^ |
| rs2046045 | G | T | 0.413 | 0.023 | 0.023 | 3.05 × 10^-1^ |
| rs2049218 | T | C | 0.385 | 0.033 | 0.023 | 1.50 × 10^-1^ |
| rs210192 | G | A | 0.215 | 0.046 | 0.027 | 9.41 × 10^-2^ |
| rs2124594 | C | T | 0.318 | -0.036 | 0.024 | 1.31 × 10^-1^ |
| rs2235544 | A | C | 0.545 | 0.025 | 0.023 | 2.75 × 10^-1^ |
| rs229528 | T | C | 0.397 | -0.022 | 0.023 | 3.42 × 10^-1^ |
| rs2345568 | G | A | 0.371 | -0.012 | 0.023 | 6.16 × 10^-1^ |
| rs244687 | G | A | 0.747 | -0.036 | 0.026 | 1.61 × 10^-1^ |
| rs2476601 | G | A | 0.852 | -0.016 | 0.032 | 6.22 × 10^-1^ |
| rs2629649 | G | T | 0.313 | -0.016 | 0.024 | 4.96 × 10^-1^ |
| rs2739043 | G | A | 0.634 | -0.044 | 0.023 | 5.67 × 10^-2^ |
| rs28391281 | C | T | 0.546 | 0.006 | 0.023 | 7.73 × 10^-1^ |
| rs28396553 | T | C | 0.650 | -0.032 | 0.024 | 1.72 × 10^-1^ |
| rs2972343 | G | A | 0.106 | 0.017 | 0.037 | 6.54 × 10^-1^ |
| rs2976908 | G | T | 0.484 | -0.029 | 0.023 | 1.96 × 10^-1^ |
| rs30233 | A | G | 0.588 | -0.008 | 0.023 | 7.36 × 10^-1^ |
| rs3103991 | G | A | 0.749 | 0.001 | 0.026 | 9.85 × 10^-1^ |
| rs35717611 | T | C | 0.379 | 0.009 | 0.023 | 7.03 × 10^-1^ |
| rs3778752 | T | G | 0.442 | -0.004 | 0.023 | 8.51 × 10^-1^ |
| rs3946137 | G | A | 0.350 | 0.012 | 0.024 | 5.99 × 10^-1^ |
| rs41177 | A | G | 0.404 | -0.034 | 0.023 | 1.34 × 10^-1^ |
| rs4606850 | T | C | 0.165 | 0.008 | 0.03 | 7.99 × 10^-1^ |

| **SNPs** | **Effect allele** | **Baseline allele** | **EAF** | **Beta** | **SE** | ***P*-value** |
| --- | --- | --- | --- | --- | --- | --- |
| rs4704447 | G | A | 0.569 | 0.007 | 0.023 | 7.57 × 10^-1^ |
| rs4853459 | C | T | 0.769 | -0.028 | 0.027 | 3.00 × 10^-1^ |
| rs4912068 | G | C | 0.089 | -0.068 | 0.04 | 8.63 × 10^-2^ |
| rs56011703 | T | C | 0.082 | 0.037 | 0.041 | 3.61 × 10^-1^ |
| rs56159866 | T | C | 0.232 | 0.043 | 0.027 | 1.07 × 10^-1^ |
| rs568999 | C | G | 0.315 | -0.046 | 0.024 | 5.83 × 10^-2^ |
| rs56983610 | A | C | 0.131 | -0.015 | 0.033 | 6.41 × 10^-1^ |
| rs57652885 | T | C | 0.053 | -0.042 | 0.051 | 4.09 × 10^-1^ |
| rs61201527 | C | A | 0.125 | -0.031 | 0.034 | 3.69 × 10^-1^ |
| rs61759532 | T | C | 0.19 | 0.071 | 0.029 | 1.27 × 10^-2^ |
| rs61916675 | G | A | 0.317 | 0.027 | 0.024 | 2.61 × 10^-1^ |
| rs61938962 | T | C | 0.32 | -0.027 | 0.024 | 2.68 × 10^-1^ |
| rs6448432 | A | G | 0.283 | 0.028 | 0.025 | 2.62 × 10^-1^ |
| rs6471875 | A | C | 0.406 | 0.003 | 0.023 | 8.98 × 10^-1^ |
| rs66760320 | T | C | 0.204 | 0.010 | 0.028 | 7.28 × 10^-1^ |
| rs6831973 | C | T | 0.556 | 0.051 | 0.023 | 2.42 × 10^-2^ |
| rs7043516 | C | A | 0.166 | -0.015 | 0.030 | 6.24 × 10^-1^ |
| rs706779 | C | T | 0.376 | -0.050 | 0.023 | 3.07 × 10^-2^ |
| rs7127620 | G | A | 0.359 | -0.007 | 0.023 | 7.67 × 10^-1^ |
| rs713427 | C | T | 0.222 | -0.020 | 0.027 | 4.62 × 10^-1^ |
| rs71430783 | T | G | 0.179 | -0.010 | 0.029 | 7.38 × 10^-1^ |
| rs71641308 | T | C | 0.097 | 0.080 | 0.038 | 3.69 × 10^-2^ |
| rs72729322 | T | C | 0.211 | 0.011 | 0.028 | 6.92 × 10^-1^ |
| rs72751538 | T | G | 0.066 | -0.053 | 0.045 | 2.41 × 10^-1^ |
| rs735000 | T | C | 0.120 | -0.030 | 0.035 | 3.84 × 10^-1^ |
| rs7444908 | G | A | 0.357 | 0.007 | 0.023 | 7.51 × 10^-1^ |
| rs753760 | G | C | 0.262 | 0.021 | 0.025 | 4.06 × 10^-1^ |
| rs76032549 | T | C | 0.170 | -0.016 | 0.030 | 5.83 × 10^-1^ |
| rs76169968 | A | G | 0.099 | -0.059 | 0.038 | 1.16 × 10^-1^ |
| rs7701443 | G | A | 0.380 | -0.042 | 0.023 | 6.90 × 10^-2^ |
| rs774121 | C | T | 0.750 | -0.028 | 0.026 | 2.75 × 10^-1^ |
| rs78953577 | T | G | 0.249 | 0.011 | 0.026 | 6.69 × 10^-1^ |
| rs7902146 | T | C | 0.750 | -0.021 | 0.026 | 4.20 × 10^-1^ |
| rs79051898 | T | C | 0.120 | 0.034 | 0.035 | 3.22 × 10^-1^ |
| rs794999 | G | A | 0.771 | -0.042 | 0.027 | 1.15 × 10^-1^ |
| rs8006310 | G | A | 0.505 | 0.010 | 0.022 | 6.43 × 10^-1^ |
| rs8193 | T | C | 0.353 | 0.017 | 0.023 | 4.60 × 10^-1^ |
| rs914960 | A | T | 0.150 | 0.040 | 0.031 | 2.00 × 10^-1^ |
| rs9292 | G | A | 0.968 | -0.003 | 0.065 | 9.62 × 10^-1^ |
| rs9378805 | C | A | 0.470 | -0.014 | 0.023 | 5.47 × 10^-1^ |
| rs938726 | C | A | 0.731 | -0.040 | 0.025 | 1.17 × 10^-1^ |
| rs9497965 | T | C | 0.337 | 0.013 | 0.024 | 5.86 × 10^-1^ |

| **SNPs** | **Effect allele** | **Baseline allele** | **EAF** | **Beta** | **SE** | ***P*-value** |
| --- | --- | --- | --- | --- | --- | --- |
| rs9981704 | T | C | 0.180 | 0.069 | 0.029 | 1.76 × 10^-2^ |
| **20 SNPs for type 1 diabetes mellitus on sarcoidosis** | | | | | | |
| rs114313544 | C | A | 0.050 | 0.054 | 0.052 | 3.02 × 10^-1^ |
| rs114355928 | T | G | 0.033 | -0.021 | 0.063 | 7.43 × 10^-1^ |
| rs11571297 | C | T | 0.372 | -0.04 | 0.023 | 8.65 × 10^-2^ |
| rs116965083 | T | C | 0.042 | -0.01 | 0.056 | 8.51 × 10^-1^ |
| rs11964319 | T | C | 0.326 | 0.055 | 0.024 | 2.20 × 10^-2^ |
| rs140149 | T | C | 0.348 | 0.035 | 0.024 | 1.32 × 10^-1^ |
| rs151234 | C | G | 0.124 | -0.048 | 0.034 | 1.63 × 10^-1^ |
| rs1639106 | T | C | 0.036 | 0.140 | 0.06 | 1.88 × 10^-2^ |
| rs17179816 | T | C | 0.042 | -0.044 | 0.055 | 4.31 × 10^-1^ |
| rs17200067 | T | C | 0.034 | -0.022 | 0.062 | 7.23 × 10^-1^ |
| rs1985706 | G | A | 0.151 | 0.053 | 0.032 | 9.32 × 10^-2^ |
| rs2476601 | G | A | 0.852 | -0.016 | 0.032 | 6.22 × 10^-1^ |
| rs28895017 | A | C | 0.011 | -0.049 | 0.107 | 6.47 × 10^-1^ |
| rs56994090 | C | T | 0.494 | -0.016 | 0.023 | 4.82 × 10^-1^ |
| rs601338 | A | G | 0.375 | 0.055 | 0.023 | 1.67 × 10^-2^ |
| rs60888743 | G | A | 0.230 | -0.003 | 0.027 | 9.22 × 10^-1^ |
| rs7130222 | G | T | 0.262 | 0.037 | 0.025 | 1.47 × 10^-1^ |
| rs74203920 | T | C | 0.037 | 0.079 | 0.061 | 1.95 × 10^-1^ |
| rs876497 | A | G | 0.280 | 0.041 | 0.025 | 9.77 × 10^-2^ |
| rs9934969 | A | G | 0.303 | -0.035 | 0.025 | 1.48 × 10^-1^ |
| **9 SNPs for coeliac disease on sarcoidosis** | | | | | | |
| rs2230365 | T | C | 0.170 | -0.149 | 0.031 | 1.44 × 10^-6^ |
| rs67572105 | A | G | 0.071 | 0.019 | 0.044 | 6.71 × 10^-1^ |
| rs68006638 | A | G | 0.047 | 0.232 | 0.049 | 2.18 × 10^-6^ |
| rs6822844 | T | G | 0.114 | -0.096 | 0.036 | 7.40 × 10^-3^ |
| rs6917350 | A | G | 0.142 | 0.041 | 0.032 | 2.03 × 10^-1^ |
| rs6933404 | C | T | 0.189 | 0.068 | 0.028 | 1.73 × 10^-2^ |
| rs71327040 | T | G | 0.107 | 0.036 | 0.036 | 3.21 × 10^-1^ |
| rs7758498 | A | G | 0.136 | -0.014 | 0.034 | 6.79 × 10^-1^ |
| rs9488932 | T | C | 0.261 | -0.001 | 0.025 | 9.66 × 10^-1^ |
| **35 SNPs for inflammatory bowel disease on sarcoidosis** | | | | | | |
| rs10737481 | G | T | 0.51 | 0.021 | 0.022 | 3.40 × 10^-1^ |
| rs10761659 | G | A | 0.528 | 0.015 | 0.022 | 5.14 × 10^-1^ |
| rs10931828 | T | C | 0.441 | -0.014 | 0.023 | 5.43 × 10^-1^ |
| rs11590283 | A | G | 0.737 | -0.048 | 0.025 | 5.70 × 10^-2^ |
| rs117115824 | T | A | 0.102 | -0.003 | 0.039 | 9.42 × 10^-1^ |
| rs11771806 | T | C | 0.117 | 0.052 | 0.035 | 1.37 × 10^-1^ |
| rs12132298 | C | T | 0.213 | -0.001 | 0.027 | 9.72 × 10^-1^ |
| rs12536069 | C | T | 0.068 | 0.13 | 0.043 | 2.45 × 10^-3^ |
| rs12755372 | C | A | 0.25 | -0.036 | 0.026 | 1.66 × 10^-1^ |

| **SNPs** | **Effect allele** | **Baseline allele** | **EAF** | **Beta** | **SE** | ***P*-value** |
| --- | --- | --- | --- | --- | --- | --- |
| rs13165038 | C | T | 0.312 | -0.036 | 0.024 | 1.35 × 10^-1^ |
| rs142695953 | A | C | 0.186 | 0.083 | 0.029 | 3.59 × 10^-3^ |
| rs191615076 | A | C | 0.040 | -0.082 | 0.058 | 1.61 × 10^-1^ |
| rs1990760 | T | C | 0.586 | 0.017 | 0.023 | 4.61 × 10^-1^ |
| rs2735075 | G | T | 0.424 | 0.004 | 0.023 | 8.60 × 10^-1^ |
| rs2823272 | A | T | 0.257 | -0.047 | 0.026 | 6.58 × 10^-2^ |
| rs2836883 | A | G | 0.247 | -0.004 | 0.026 | 8.74 × 10^-1^ |
| rs3197999 | A | G | 0.392 | -0.006 | 0.023 | 7.81 × 10^-1^ |
| rs3736162 | C | G | 0.281 | -0.069 | 0.025 | 6.16 × 10^-3^ |
| rs376365394 | T | C | 0.040 | -0.083 | 0.058 | 1.49 × 10^-1^ |
| rs3827023 | T | C | 0.188 | -0.033 | 0.029 | 2.51 × 10^-1^ |
| rs4372078 | G | T | 0.739 | 0.043 | 0.026 | 9.40 × 10^-2^ |
| rs4676410 | A | G | 0.271 | -0.005 | 0.025 | 8.46 × 10^-1^ |
| rs4730275 | T | G | 0.334 | -0.010 | 0.024 | 6.88 × 10^-1^ |
| rs4731531 | A | G | 0.443 | 0.042 | 0.023 | 6.52 × 10^-2^ |
| rs4807543 | T | G | 0.032 | 0.040 | 0.064 | 5.32 × 10^-1^ |
| rs56893428 | T | C | 0.451 | -0.011 | 0.023 | 6.25 × 10^-1^ |
| rs6017342 | C | A | 0.559 | 0.031 | 0.023 | 1.65 × 10^-1^ |
| rs6556416 | C | A | 0.742 | -0.008 | 0.026 | 7.50 × 10^-1^ |
| rs6762648 | A | T | 0.244 | -0.015 | 0.026 | 5.77 × 10^-1^ |
| rs7551957 | C | T | 0.446 | -0.013 | 0.023 | 5.57 × 10^-1^ |
| rs7936434 | C | G | 0.414 | -0.004 | 0.023 | 8.47 × 10^-1^ |
| rs895123 | G | C | 0.194 | -0.005 | 0.028 | 8.56 × 10^-1^ |
| rs9272353 | C | G | 0.193 | -0.086 | 0.029 | 2.94 × 10^-3^ |
| rs9607629 | G | A | 0.125 | 0.003 | 0.034 | 9.24 × 10^-1^ |
| rs9617090 | T | C | 0.372 | 0.018 | 0.023 | 4.38 × 10^-1^ |
| **20 SNPs for psoriasis on sarcoidosis** | | | | | | |
| rs12206050 | T | A | 0.320 | -0.028 | 0.024 | 2.54 × 10^-1^ |
| rs1250566 | A | G | 0.408 | 0.042 | 0.023 | 6.91 × 10^-2^ |
| rs13153019 | C | T | 0.273 | 0.018 | 0.025 | 4.78 × 10^-1^ |
| rs139298380 | A | G | 0.028 | 0.032 | 0.068 | 6.38 × 10^-1^ |
| rs1611704 | T | C | 0.345 | -0.019 | 0.024 | 4.15 × 10^-1^ |
| rs16903065 | A | C | 0.115 | -0.088 | 0.035 | 1.33 × 10^-2^ |
| rs2769979 | C | T | 0.605 | -0.003 | 0.023 | 8.96 × 10^-1^ |
| rs28998802 | A | G | 0.184 | -0.053 | 0.029 | 6.80 × 10^-2^ |
| rs33980500 | T | C | 0.072 | -0.045 | 0.043 | 3.01 × 10^-1^ |
| rs4400255 | A | T | 0.099 | 0.005 | 0.037 | 8.96 × 10^-1^ |
| rs60600003 | G | T | 0.102 | 0.068 | 0.037 | 6.65 × 10^-2^ |
| rs653169 | G | A | 0.570 | -0.005 | 0.023 | 8.30 × 10^-1^ |
| rs674451 | C | T | 0.344 | 0.007 | 0.024 | 7.55 × 10^-1^ |
| rs74817271 | A | G | 0.074 | -0.034 | 0.042 | 4.29 × 10^-1^ |
| rs7542079 | C | T | 0.562 | -0.011 | 0.023 | 6.34 × 10^-1^ |

| **SNPs** | **Effect allele** | **Baseline allele** | **EAF** | **Beta** | **SE** | ***P*-value** |
| --- | --- | --- | --- | --- | --- | --- |
| rs76741620 | G | A | 0.058 | 0.037 | 0.048 | 4.41 × 10^-1^ |
| rs78456138 | T | C | 0.020 | -0.061 | 0.080 | 4.49 × 10^-1^ |
| rs847 | C | T | 0.635 | 0.011 | 0.023 | 6.40 × 10^-1^ |
| rs8904 | A | G | 0.388 | -0.009 | 0.023 | 7.12 × 10^-1^ |
| rs9346778 | T | C | 0.164 | 0.031 | 0.030 | 3.11 × 10^-1^ |
| **6 SNPs for anterior iridocyclitis on sarcoidosis** | | | | | | |
| rs12194518 | G | C | 0.101 | 0.018 | 0.038 | 6.26 × 10^-1^ |
| rs1233398 | T | C | 0.190 | -0.028 | 0.029 | 3.26 × 10^-1^ |
| rs13160562 | A | G | 0.272 | -0.04 | 0.025 | 1.09 × 10^-1^ |
| rs190364070 | T | C | 0.054 | 0.030 | 0.051 | 5.54 × 10^-1^ |
| rs296563 | G | T | 0.727 | 0.004 | 0.025 | 8.79 × 10^-1^ |
| rs303888 | G | A | 0.433 | -0.079 | 0.023 | 5.96 × 10^-4^ |
| **3 SNPs for sarcoidosis on autoimmune diseases** | | | | | | |
| rs1079242 | G | A | 0.561 | -0.011 | 0.005 | 4.82 × 10^-2^ |
| rs1860217 | C | G | 0.419 | 0.017 | 0.005 | 1.83 × 10^-3^ |
| rs7581601 | C | A | 0.297 | -0.002 | 0.006 | 7.58 × 10^-1^ |
| **4 SNPs for sarcoidosis on rheumatoid arthritis** | | | | | | |
| rs1079242 | G | A | 0.561 | -0.013 | 0.013 | 3.22 × 10^-1^ |
| rs11757273 | A | C | 0.393 | -0.021 | 0.014 | 1.27 × 10^-1^ |
| rs1860217 | C | G | 0.419 | 0.022 | 0.013 | 9.42 × 10^-2^ |
| rs2024825 | C | T | 0.836 | 0.033 | 0.018 | 6.35 × 10^-2^ |
| **4 SNPs for sarcoidosis on hypothyroidism** | | | | | | |
| rs1079242 | G | A | 0.561 | -0.015 | 0.008 | 5.71 × 10^-2^ |
| rs1611637 | G | A | 0.519 | 0.003 | 0.008 | 6.67 × 10^-1^ |
| rs2024825 | C | T | 0.836 | -0.015 | 0.011 | 1.44 × 10^-1^ |
| rs7581601 | C | A | 0.298 | -0.005 | 0.009 | 5.88 × 10^-1^ |
| **4 SNPs for sarcoidosis on type 1 diabetes mellitus** | | | | | | |
| rs11757273 | A | C | 0.394 | 0.090 | 0.023 | 6.97 × 10^-5^ |
| rs1860217 | C | G | 0.419 | 0.025 | 0.023 | 2.68 × 10^-1^ |
| rs34536443 | C | G | 0.030 | -0.218 | 0.071 | 2.23 × 10^-3^ |
| rs663743 | A | G | 0.375 | -0.050 | 0.023 | 3.16 × 10^-2^ |
| **5 SNPs for sarcoidosis on coeliac disease** | | | | | | |
| rs1079242 | G | A | 0.561 | -0.044 | 0.024 | 6.93 × 10^-2^ |
| rs1860217 | C | G | 0.419 | -0.001 | 0.024 | 9.61 × 10^-1^ |
| rs2024825 | C | T | 0.836 | 0.013 | 0.032 | 6.83 × 10^-1^ |
| rs34536443 | C | G | 0.030 | -0.249 | 0.075 | 9.08 × 10^-4^ |
| rs7581601 | C | A | 0.298 | 0.035 | 0.026 | 1.73 × 10^-1^ |
| **7 SNPs for sarcoidosis on inflammatory bowel disease** | | | | | | |
| rs11757273 | A | C | 0.394 | -0.007 | 0.017 | 6.93 × 10^-1^ |
| rs1431403 | C | T | 0.245 | 0.013 | 0.019 | 5.11 × 10^-1^ |
| rs1860217 | C | G | 0.419 | 0.013 | 0.017 | 4.19 × 10^-1^ |
| rs34536443 | C | G | 0.030 | -0.155 | 0.051 | 2.26 × 10^-3^ |

| **SNPs** | **Effect allele** | **Baseline allele** | **EAF** | **Beta** | **SE** | ***P*-value** |
| --- | --- | --- | --- | --- | --- | --- |
| rs4455663 | C | T | 0.391 | -0.043 | 0.017 | 9.93 × 10^-3^ |
| rs663743 | A | G | 0.375 | -0.007 | 0.017 | 6.89 × 10^-1^ |
| rs7581601 | C | A | 0.297 | 0.042 | 0.018 | 1.82 × 10^-2^ |
| **5 SNPs for sarcoidosis on psoriasis** | | | | | | |
| rs1079242 | G | A | 0.561 | 0.003 | 0.015 | 8.57 × 10^-1^ |
| rs11757273 | A | C | 0.394 | 0.019 | 0.015 | 2.11 × 10^-1^ |
| rs1860217 | C | G | 0.419 | -0.019 | 0.015 | 2.14 × 10^-1^ |
| rs2024825 | C | T | 0.836 | 0.046 | 0.020 | 2.23 × 10^-2^ |
| rs7581601 | C | A | 0.297 | 0.001 | 0.016 | 9.58 × 10^-1^ |
| **6 SNPs for sarcoidosis on anterior iridocyclitis** | | | | | | |
| rs1079242 | G | A | 0.561 | -0.018 | 0.018 | 3.23 × 10^-1^ |
| rs11757273 | A | C | 0.394 | -0.003 | 0.018 | 8.64 × 10^-1^ |
| rs1860217 | C | G | 0.419 | 0.020 | 0.018 | 2.60 × 10^-1^ |
| rs2024825 | C | T | 0.836 | -0.013 | 0.024 | 5.77 × 10^-1^ |
| rs663743 | A | G | 0.375 | -0.067 | 0.018 | 2.61 × 10^-4^ |
| rs7581601 | C | A | 0.297 | 0.011 | 0.019 | 5.53 × 10^-1^ |

The dataset of autoimmune diseases analyzed in this study comprised a total of 44 different types of autoimmune-related diseases. A *P*-value < 5 × 10^−8^ was considered genome-wide significant and should be excluded to meet the assumption that requires instruments to be associated with the outcome only through exposure. Abbreviations: MR: Mendelian randomization; SNPs: Single nucleotide polymorphisms; EAF: Effect allele frequency; SE: Standard error.

**Supplementary Table 6 MR results for the relationship between autoimmune diseases and sarcoidosis**

| **Methods** | **Number of SNPs** | **OR (95% CI)** | ***P*-value** |
| --- | --- | --- | --- |
| **Autoimmune diseases on sarcoidosis** | | | |
| MR-Egger | 87 | 1.82 (1.23 to 2.70) | 3.59 × 10^-3^ |
| Weighted median |  | 1.57 (1.30 to 1.89) | 2.07 × 10^-6^ |
| IVW-FE |  | 1.79 (1.59 to 2.02) | 1.01 × 10^-21^ |
| Simple mode |  | 1.45 (0.88 to 2.38) | 1.52 × 10^-1^ |
| Weighted mode |  | 1.40 (0.95 to 2.05) | 9.42 × 10^-2^ |
| **Rheumatoid arthritis on sarcoidosis** | | | |
| MR-Egger | 11 | 0.84 (0.48 to 1.45) | 5.42 × 10^-1^ |
| Weighted median |  | 0.95 (0.77 to 1.17) | 6.05 × 10^-1^ |
| IVW-FE |  | 0.94 (0.81 to 1.10) | 4.51 × 10^-1^ |
| Simple mode |  | 0.92 (0.64 to 1.31) | 6.46 × 10^-1^ |
| Weighted mode |  | 0.92 (0.66 to 1.27) | 6.31 × 10^-1^ |
| **Autoimmune hypothyroidism on sarcoidosis** | | | |
| MR-Egger | 108 | 1.07 (0.91 to 1.26) | 8.19 × 10^-2^ |
| Weighted median |  | 1.10 (0.99 to 1.23) | 5.47 × 10^-2^ |
| IVW-FE |  | 1.13 (1.06 to 1.21) | 3.21 × 10^-2^ |
| Simple mode |  | 1.12 (0.90 to 1.42) | 1.16 × 10^-1^ |
| Weighted mode |  | 1.11 (0.99 to 1.26) | 6.09 × 10^-2^ |
| **Type 1 diabetes mellitus on sarcoidosis** | | | |
| MR-Egger | 20 | 1.02 (0.92 to 1.12) | 7.19 × 10^-1^ |
| Weighted median |  | 1.04 (0.97 to 1.12) | 3.06 × 10^-1^ |
| IVW-FE |  | 1.09 (1.04 to 1.15) | 4.89 × 10^-4^ |
| Simple mode |  | 1.27 (1.11 to 1.45) | 2.70 × 10^-3^ |
| Weighted mode |  | 1.04 (0.97 to 1.12) | 2.60 × 10^-1^ |
| **Coeliac disease on sarcoidosis** | | | |
| MR-Egger | 9 | 1.25 (1.10 to 1.43) | 1.27 × 10^-2^ |
| Weighted median |  | 1.22 (1.13 to 1.31) | 2.81 × 10^-7^ |
| IVW-FE |  | 1.22 (1.15 to 1.28) | 7.29 × 10^-12^ |
| Simple mode |  | 1.08 (0.93 to 1.26) | 3.55 × 10^-1^ |
| Weighted mode |  | 1.23 (1.13 to 1.33) | 1.02 × 10^-3^ |
| **Inflammatory bowel disease on sarcoidosis** | | | |
| MR-Egger | 35 | 1.14 (0.84 to 1.54) | 3.97 × 10^-1^ |
| Weighted median |  | 1.10 (1.00 to 1.21) | 4.63 × 10^-2^ |
| IVW-MRE |  | 1.15 (1.06 to 1.23) | 3.32 × 10^-4^ |
| Simple mode |  | 1.02 (0.82 to 1.26) | 8.79 × 10^-1^ |
| Weighted mode |  | 1.01 (0.83 to 1.23) | 9.18 × 10^-1^ |
| **Psoriasis on sarcoidosis** | | | |
| MR-Egger | 20 | 1.02 (0.77 to 1.35) | 9.15 × 10^-1^ |
| Weighted median |  | 1.01 (0.89 to 1.14) | 9.24 × 10^-1^ |
| IVW-FE |  | 0.98 (0.89 to 1.07) | 6.96 × 10^-1^ |

| **Method** | **Number of SNPs** | **OR (95% CI)** | ***P*-value** |
| --- | --- | --- | --- |
| Simple mode |  | 1.07 (0.86 to 1.33) | 5.48 × 10^-1^ |
| Weighted mode |  | 1.01 (0.85 to 1.19) | 9.41 × 10^-1^ |
| **Anterior iridocyclitis on sarcoidosis** | | | |
| MR-Egger | 6 | 0.98 (0.79 to 1.20) | 8.35 × 10^-1^ |
| Weighted median |  | 1.03 (0.94 to 1.14) | 5.04 × 10^-1^ |
| IVW-MRE |  | 1.07 (0.94 to 1.22) | 2.91 × 10^-1^ |
| Simple mode |  | 1.04 (0.88 to 1.22) | 6.76 × 10^-1^ |
| Weighted mode |  | 1.03 (0.93 to 1.15) | 2.50 × 10^-1^ |
| **Sarcoidosis on autoimmune diseases** | | | |
| MR-Egger | 3 | 1.02 (0.77 to 1.34) | 9.11 × 10^-1^ |
| Weighted median |  | 1.04 (1.00 to 1.09) | 4.89 × 10^-2^ |
| IVW-MRE |  | 1.05 (0.99 to 1.12) | 9.88 × 10^-2^ |
| Simple mode |  | 1.03 (0.97 to 1.09) | 4.41 × 10^-1^ |
| Weighted mode |  | 1.04 (0.99 to 1.09) | 2.35 × 10^-1^ |
| **Sarcoidosis on rheumatoid arthritis** | | | |
| MR-Egger | 4 | 0.89 (0.45 to 1.77) | 7.72 × 10^-1^ |
| Weighted median |  | 0.99 (0.90 to 1.08) | 7.57 × 10^-1^ |
| IVW-MRE |  | 0.99 (0.88 to 1.12) | 8.77 × 10^-1^ |
| Simple mode |  | 0.90 (0.74 to 1.10) | 3.95 × 10^-1^ |
| Weighted mode |  | 1.02 (0.91 to 1.14) | 7.52 × 10^-1^ |
| **Sarcoidosis on autoimmune hypothyroidism** | | | |
| MR-Egger | 4 | 1.16 (0.98 to 1.37) | 2.24 × 10^-1^ |
| Weighted median |  | 1.06 (1.00 to 1.11) | 3.17 × 10^-2^ |
| IVW-FE |  | 1.05 (1.00 to 1.10) | 4.64 × 10^-2^ |
| Simple mode |  | 1.06 (0.99 to 1.14) | 2.06 × 10^-1^ |
| Weighted mode |  | 1.06 (1.00 to 1.13) | 1.37 × 10^-1^ |
| **Sarcoidosis on type 1 diabetes mellitus** | | | |
| MR-Egger | 4 | 1.99 (1.24 to 3.22) | 1.06 × 10^-1^ |
| Weighted median |  | 1.47 (1.23 to 1.76) | 2.45 × 10^-5^ |
| IVW-FE |  | 1.46 (1.27 to 1.69) | 1.15 × 10^-7^ |
| Simple mode |  | 1.60 (1.21 to 2.11) | 4.52 × 10^-2^ |
| Weighted mode |  | 1.58 (1.21 to 2.08) | 4.21 × 10^-2^ |
| **Sarcoidosis on coeliac disease** | | | |
| MR-Egger | 5 | 1.61 (0.93 to 2.79) | 1.87 × 10^-1^ |
| Weighted median |  | 1.16 (0.98 to 1.37) | 8.08 × 10^-2^ |
| IVW-FE |  | 1.19 (1.05 to 1.35) | 6.53 × 10^-3^ |
| Simple mode |  | 1.10 (0.86 to 1.39) | 4.92 × 10^-1^ |
| Weighted mode |  | 1.14 (0.94 to 1.38) | 2.44 × 10^-1^ |
| **Sarcoidosis on** **inflammatory bowel disease** | | | |
| MR-Egger | 7 | 1.16 (0.86 to 1.58) | 3.74 × 10^-1^ |
| Weighted median |  | 1.08 (0.99 to 1.18) | 1.02 × 10^-1^ |
| IVW-MRE |  | 1.12 (1.02 to 1.22) | 1.83 × 10^-2^ |

| **Method** | **Number of SNPs** | **OR (95% CI)** | ***P*-value** |
| --- | --- | --- | --- |
| Simple mode |  | 1.05 (0.91 to 1.22) | 5.14 × 10^-1^ |
| Weighted mode |  | 1.05 (0.94 to 1.17) | 4.46 × 10^-1^ |
| **Sarcoidosis on psoriasis** | | | |
| MR-Egger | 5 | 1.02 (0.62 to 1.68) | 9.53 × 10^-1^ |
| Weighted median |  | 0.99 (0.90 to 1.10) | 8.65 × 10^-1^ |
| IVW-FE |  | 0.97 (0.90 to 1.04) | 4.04 × 10^-1^ |
| Simple mode |  | 1.00 (0.83 to 1.21) | 9.71 × 10^-1^ |
| Weighted mode |  | 1.01 (0.89 to 1.14) | 9.37 × 10^-1^ |
| **Sarcoidosis on anterior iridocyclitis** | | | |
| MR-Egger | 6 | 0.96 (0.56 to 1.64) | 8.78 × 10^-1^ |
| Weighted median |  | 1.07 (0.96 to 1.20) | 2.12 × 10^-1^ |
| IVW-FE |  | 1.12 (1.03 to 1.22) | 7.41 × 10^-3^ |
| Simple mode |  | 1.08 (0.93 to 1.25) | 3.59 × 10^-1^ |
| Weighted mode |  | 1.07 (0.95 to 1.21) | 3.04 × 10^-1^ |

The dataset of autoimmune diseases analyzed in this study comprised a total of 44 different types of autoimmune-related diseases. A multiple-testing threshold of *P*-value < 6.25 × 10^-3^ (0.05/8) was adopted to declare a statistical significance using the Bonferroni method. Abbreviations: MR: Mendelian randomization; SNPs: Single nucleotide polymorphisms; OR: Odds ratio; CI: Confidence intervals; IVW-MRE: Multiplicative random-effect inverse variance weighted; IVW-FE: Fixed-effect inverse variance weighted.

**Supplementary Table 7 Results of** **MR Steiger direction test for outcomes on exposures**

| **Exposures** | **Outcomes** | **r^2^ exposure** | **r^2^ outcome** | **Correct causal direction** | ***P*-value** |
| --- | --- | --- | --- | --- | --- |
| Autoimmune diseases | Sarcoidosis | 1.23 × 10^-2^ | 5.24 × 10^-4^ | TRUE | ＜0.001 |
| Rheumatoid arthritis |  | 1.90 × 10^-3^ | 2.02 × 10^-5^ | TRUE | ＜0.001 |
| Autoimmune hypothyroidism |  | 2.69 × 10^-2^ | 4.06 × 10^-4^ | TRUE | ＜0.001 |
| Type 1 diabetes mellitus |  | 4.70 × 10^-3^ | 1.01 × 10^-4^ | TRUE | ＜0.001 |
| Coeliac disease |  | 4.23 × 10^-3^ | 1.64 × 10^-4^ | TRUE | ＜0.001 |
| Inflammatory bowel disease |  | 4.87 × 10^-3^ | 1.75 × 10^-4^ | TRUE | ＜0.001 |
| Psoriasis |  | 3.19 × 10^-3^ | 6.55 × 10^-5^ | TRUE | ＜0.001 |
| Anterior iridocyclitis |  | 3.60 × 10^-3^ | 4.25 × 10^-5^ | TRUE | ＜0.001 |
| Sarcoidosis | Autoimmune diseases | 4.99 × 10^-4^ | 3.63 × 10^-5^ | TRUE | ＜0.001 |
|  | Rheumatoid arthritis | 7.33 × 10^-4^ | 3.77 × 10^-5^ | TRUE | ＜0.001 |
|  | Autoimmune hypothyroidism | 6.39 × 10^-4^ | 1.98 × 10^-5^ | TRUE | ＜0.001 |
|  | Type 1 diabetes mellitus | 5.06 × 10^-4^ | 9.93 × 10^-5^ | TRUE | ＜0.001 |
|  | Coeliac disease | 7.23 × 10^-4^ | 4.48 × 10^-5^ | TRUE | ＜0.001 |
|  | Inflammatory bowel disease | 1.33 × 10^-3^ | 6.08 × 10^-5^ | TRUE | ＜0.001 |
|  | Psoriasis | 8.16 × 10^-4^ | 2.24 × 10^-5^ | TRUE | ＜0.001 |
|  | Anterior iridocyclitis | 9.60 × 10^-4^ | 4.31 × 10^-5^ | TRUE | ＜0.001 |

The dataset of autoimmune diseases analyzed in this study comprised a total of 44 different types of autoimmune-related diseases. Abbreviations: MR: Mendelian randomization.
